# Multivariate phenomenological models for real-time short-term forecasts of hospital capacity for COVID-19 in Belgium from March to June 2020

**DOI:** 10.1017/S0950268821002491

**Published:** 2021-12-17

**Authors:** M. H. Nguyen, T. Braeye, N. Hens, C. Faes

**Affiliations:** 1 Data Science Institute (DSI), I-BioStat, Universiteit Hasselt, BE-3500 Hasselt, Belgium; 2 Department of Epidemiology and Public Health, Sciensano, BE-1050 Brussels, Belgium; 3 Centre for Health Economics Research and Modelling of Infectious Diseases (CHERMID), Vaccine & Infectious Disease Institute (VAXINFECTIO), University of Antwerp, BE-2000 Antwerp, Belgium

**Keywords:** COVID-19, modelling, public health emerging infections, statistics

## Abstract

Phenomenological models are popular for describing the epidemic curve. We present how they can be used at different phases in the epidemic, by modelling the daily number of new hospitalisations (or cases). As real-time prediction of the hospital capacity is important, a joint model of the new hospitalisations, number of patients in hospital and in intensive care unit (ICU) is proposed. This model allows estimation of the length of stay in hospital and ICU, even if no (or limited) individual level information on length of stay is available. Estimation is done in a Bayesian framework. In this framework, real-time alarms, defined as the probability of exceeding hospital capacity, can be easily derived. The methods are illustrated using data from the COVID-19 pandemic in March–June 2020 in Belgium, but are widely applicable.

## Introduction

Since the first outbreak of corona virus disease 2019 (COVID-19) in China in December 2020, many countries are struggling to get the ongoing outbreak under control. Worldwide, researchers are using epidemic models to generate short- and long-term forecasts as these are crucial for public health-care decision makers. Indeed, it is of paramount importance to understand how the outbreak is evolving and to make predictions, such that health authorities can plan the response to the outbreak. One of the major issues with COVID-19 is the high hospitalisation rate. In many countries, including Belgium, a large number of hospitalisations of COVID-19 patients has forced hospitals to postpone regular care of non-COVID-19 patients. The authors in [1, 2] indicate that a timely intervention is needed to preserve the hospital capacity. It is therefore crucial to track and predict also the total number of patients in the hospital and in the intensive care units (ICUs).

Mathematical and statistical models are commonly used to describe the epidemic, and derive epidemiological parameters of the outbreak. Important parameters describing the evolution of the outbreak are the growth rate, the number of hospitalisations at the peak of the outbreak, the turning point, the final size of the epidemic and length of the epidemic wave. The analysis of the outbreak in real time is challenging because of different phases in the epidemic curve, in which often only limited information is available. At the start, the epidemic is described by exponential growth, followed by a slowing down of the growth due to intervention measures. If control measures are successful, a turning point can be observed followed by a decline in the number of new cases and hospitalisations. The prediction model should adapt to these different phases. Chowell discusses some phenomenological models to characterise and forecast the cumulative number of cases and connects these models to ordinary differential equations describing the dynamics of the epidemic [3, 4]. Commonly used phenomenological models are the exponential model, the three-parameter logistic [5] and the Richard model [6–9], which have been applied for epidemics of severe acute respiratory syndrome coronavirus 2 (SARS-CoV-1) [7], Ebola [10], Zika [11] and SARS-CoV-2 [12]. Sebrango-Rodriquez *et al*. use model averaging based on different phenomenological models to predict final size and turning point of the epidemic in real-time while taking into account model uncertainty [11].

An outdated strategy is to fit the growth model to cumulative case counts using least squares for model fitting or likelihood estimation. King *et al*. have shown via simulations that with such methods the confidence in parameter estimates and forecasts can be far overestimated [13]. The problem with fitting the model to the cumulative case counts is due to the underlying assumption of independence of the sequential measurement errors, which is violated when observations are accumulated through time [13–16]. In this paper, the focus is therefore on modelling the daily observed new hospitalisations with parameter estimation using Bayesian Markov Chain Monte Carlo (MCMC) methods. One of the advantages of the Bayesian framework is the flexibility in which nonlinear models can be estimated, prediction intervals (PIs) of derived parameters can be obtained and fit complex models on multiple endpoints. Starting from a growth model for the daily new hospitalisations, we propose a method to forecast the required hospital capacity in terms of hospital beds and ICU beds, and calculate the risk to exceed certain thresholds in hospital capacity.

We first discuss the different phases in real-time short-term prediction based on the number of new hospitalisations, describing each phase with a different phenomenological model, and providing guidance on the use of simple phenomenological models. Then, the focus is on methods for prediction of the number of beds required in the hospital and in the ICU, and measuring exceedance probabilities of the bed capacity. Both methods in which information on the length of stay in hospital is available (univariate method) and is unavailable (multivariate analysis) are presented. Analyses are illustrated using the first wave of the COVID-19 pandemic in Belgium.

## Data

**Fig. 1.**
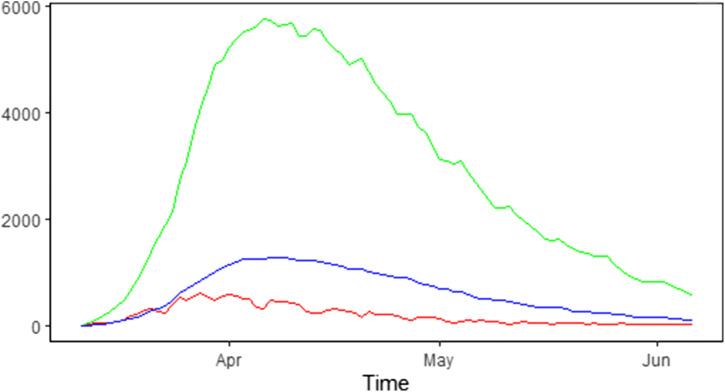
Epidemic curve in Belgium: number of new COVID-19 hospitalisations (red line), number of COVID-19 patients in the hospital (green line) and in the ICU (blue line).

The number of new hospitalisations, number of patients in hospital (denoted as the number of hospital beds) and number of patients in ICUs are collected and made publicly available by Sciensano. Data are collected daily via a hospital surge capacity survey, a mandatory survey with daily questionnaire sent to all Belgian hospitals. These numbers are aggregated to the national and provincial levels and are made available since 11 March 2020 [17]. Table 1 summarises the daily number of new hospitalisations and the daily number of beds occupied by COVID-19 patients in the hospital and ICU during the first wave of the outbreak from 11 March 2020 until 6 June 2020. The corresponding time trends are presented in Figure 1.

**Table 1.** Summary characteristics of hospital load during the first wave of the COVID-19 pandemic in Belgium from 11 March 2020 until 6 Jun 2020

Notation	Description	Mean	Range	IQR
*Y*(*t*)	Number of new COVID hospitalisations	199.1	7–629	248.7
*H*(*t*)	Total number of beds used in hospital by COVID patients	2831.8	14–5759	3537.2
*ICU*(*t*)	Total number of beds used in ICU	613.3	2–1285	776.5

## Methodology

In this section, we first present the growth curve models during four phases: (1) the exponential growth model during the initial epidemic growth phase, (2) the logistic growth model during the phase of growth to stabilisation, (3) the logistic distribution model when the turning point is reached and (4) the Richards model during the descending phase of the epidemic. We then provide some details on the estimation of the models in the Bayesian framework. Finally, we present a method to predict the hospital load, using either (1) the estimated growth curve model from the new hospitalisation in case information on the length of stay in hospital and prevalence of ICU care is available and (2) a joint model of new hospitalisations, hospital beds and ICU beds, in case information on length of stay is not readily available.

### Growth curve models

Let *Y*(*t*) denote the number of new hospitalisations on day *t* (*t* = 0, 1, …, *T*). The number of hospitalisations is a count variable and can be modelled by a Poisson distribution *Y*(*t*) ~ Poisson(*μ*(*t*)) or, to account for the heterogeneity which is typically observed during an outbreak, by a negative binomial distribution *Y*(*t*) ~ NegBin(*μ*(*t*), *θ*), with *μ*(*t*) the mean trajectory of the outbreak (mean number of new hospitalisations) and *θ* an overdispersion parameter (when *θ* → ∞, the negative binomial distribution reduces to a Poisson distribution). An important property of the Poisson distribution is that the mean and variance of *Y*(*t*) are equal (equidispersion). In real life, the equidispersion assumption does not usually hold, and the use of the negative binomial distribution which allows the variability of the data to be greater than that predicted by the Poisson model (overdispersion) is recommended in the context of epidemic data [18]. We further assume that conditional on the mean epidemic trend, the number of new cases is independent.

In a real-time data analysis, we model the number of hospitalisations using four different phases, depending on how far the epidemic curve has evolved in time. We review growth models that are useful in each of these phases and model the observed number of new hospitalisations directly (instead of modelling the cumulative number of cases *C*(*t*)). The growth models are formulated as differential equations in continuous time, although epidemic data in real life is observed in discrete time intervals. Therefore, a discrete approximation of the derivative is used, such as replacing the derivative of cumulative number of cases by the number of new cases during one day, and resulting in a discrete-time growth model.

#### Phase 1: initial epidemic growth

At the start of an outbreak, the number of new cases/hospitalisations typically grows exponentially. The rate of change in the expected number of new hospitalisations, *μ*^′^(*t*), can then be written as
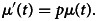


**Fig. 2.**
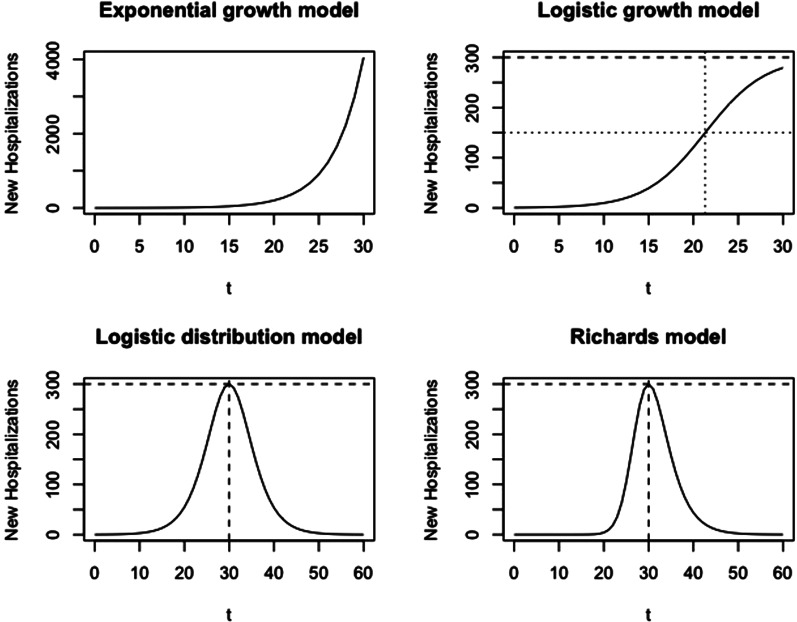
Visualisation of growth models at different phases. Phase 1: exponential growth model; phase 2: logistic growth model; phase 3: logistic distribution model; phase 4: Richards model.

This assumption is similar as in the initial phase of the mathematical SIR model. The parameter *p* represents the growth rate during this initial epidemic phase, with a corresponding doubling time of the number of new hospitalisations equal to ln(2)‒/*p*. The expected number of new hospitalisations can be derived analytically from this model and can be used to describe the early trajectory of the outbreak:
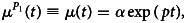
where *α* is the number of cases at time *t* = 0 and *P*_1_ refers to the phase 1 model. This model produces a J-shaped curve, as presented in Figure 2 (top left).

#### Phase 2: from growth to stabilisation

Sometime after intervention measures take place or individuals start to change social behaviour, the growth rate of new hospitalisations starts to diminish until it reaches a plateau. At the plateau, there is no more growth of the number of new hospitalisations and the number of new hospitalisations becomes constant (i.e. stabilisation). As a result, the initial exponential growth is not sustained in phase 2. In this case, the time course of the number of new hospitalisations is better described by the logistic growth model, as proposed by Verhulst [19], with the rate of change in the number of new hospitalisations described by
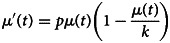
which can be expressed in terms of the expected number of new hospitalisations as
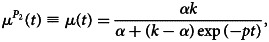
with parameters *p*, *k* and *α*, and *P*_2_ refers to the phase 2 model. This model produces an S-shaped curve (Fig. 2, top right), which increases to a maximal daily number of new hospitalisations *k* (horizontal dashed line). When *k* = ∞, this model reduces to the exponential model as described in phase 1. The parameter *p* represents the initial growth rate, which increases until time *t*_*p*_ = ln((*k* − *α*)/*α*)/*p*, at which there are *k*/2 new hospitalisations and growth rate is *pk*/4 (dotted lines), after which the growth rate decreases until the number of new hospitalisations reaches a plateau equal to *k*. The parameter *α* is the number of new hospitalisations at time *t* = 0.

#### Phase 3: turning point reached

When the outbreak is in a later phase, we might observe again a decrease in the number of new hospitalisations. In this phase, the time course of the cumulative number of hospitalisations *C*(*t*) (instead of the number of new hospitalisations) is better described by the logistic model:
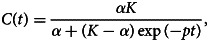
which can be re-formulated into a model for the new hospitalisations (*μ*(*t*) = *C*^′^(*t*))
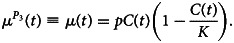


This curve is a symmetric bell-shaped curve, as presented in Figure 2 (bottom left). The curve increases to the maximal daily number of new hospitalisations *k* = *pK*/4, which is reached on the turning point *t*_*m*_ = ln((*K* − *α*)/*α*)/*p* (dashed lines), and then declines. The parameter *p* is the initial growth rate and *K* is the final size of the epidemic wave (or sometimes called the carrying capacity). As the logistic curve is symmetric, there are a total of *K*/2 hospitalisations before and after the turning point.

#### Phase 4: descending phase

When more data become available in the descending phase of the epidemic curve, we might observe that the decline in the number of new cases is slower or faster as compared to the increase. In such a case, the Richards model [6], allowing for an asymmetric epidemic curve, is better used (Fig. 2, bottom right). The Richards model is an extension of the Verhulst model with one additional parameter, and has been used in the context of real-time prediction of outbreak of diseases [7, 20], though for specifying the cumulative number of cases. The cumulative number of cases based on the model is given by
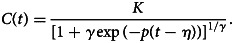


A model for the number of new hospitalisations can be derived from this by taking the derivative of this function (*μ*(*t*) = *C*^′^(*t*)), leading to
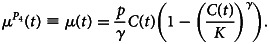


Wang *et al*. describe the connection of the Richards model with a simple epidemic SIR model. In this model, *K* is the final size of the epidemic, *p*/*γ* is the initial growth rate and *η* is the turning point of the epidemic [21]. The maximal number of new hospitalisations at the peak is *k* = *pK*/(1 + *γ*)^1+1/*γ*^ (horizontal dashed line). At this time point, there have been a total of *K*/(*γ* + 1)^1/*γ*^ hospitalisations, with 1/(*γ* + 1)^1/*γ*^ the fraction of hospitalisations during the epidemic wave that occur before the turning point. If *γ* = 1, the model reduces to the symmetric phase 3 logistic model.

### Estimation

While it would be tempting to use e.g. the Richards model in the first phase of the outbreak already, to predict the peak of the outbreak and the final size; this would lead to unreliable estimates, as no information is yet available about later phases of the outbreak; and indeed multiple models could results in the same fit to the observed cases but with major differences of the peak estimation. Therefore, the use of different models at different phases of the outbreaks is recommended, as well as using only short-term predictions (e.g. up to 5–10 days) based on these growth models.

The following process is proposed for model selection and to move from one epidemic phase to the next epidemic phase. First, it is possible only to move successively through the different phases, e.g. it is possible to move from phase 2 to phase 3, but not to move back from phase 3 to phase 2. Second, the current epidemic phase model is always compared with the next epidemic phase model using Wakaike's information criterion (WAIC). Three situation can occur when comparing the WAIC of the current model (phase *k*) with the next model (phase *k* + 1): (1) if WAIC(phase *k* + 1) − WAIC(phase *k*) < 0, use model phase *k*; (2) if 0 < WAIC(phase *k* + 1) − WAIC(phase *k*) < 2, then the phase *k* and *k* + 1 models are equally likely, and they provide best and worst case scenario predictions and (3) if WAIC(phase *k* + 1) − WAIC(phase *k*) > 2, use model phase *k* + 1. Note that this is similar to the proposal by [22] to switch from one phase to the next phase. In Section ‘Real-time analysis’, we will investigate the predictive performance of the models using different measures of predictive performance.

In order to take into account the uncertainty in the data, a Bayesian estimation method is used. Weakly informative priors were used for all parameters, namely a *N*(0, 0.01) prior for *α*_0_ and log (*p*) and a log *N*(0, 0.1) for 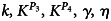. Based on samples from the posterior distribution of the parameters, the predictive distribution for the number of new cases 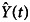 can be easily derived. Implementation of the models is done using NIMBLE (2020). NIMBLE is a system for building computationally intensive Bayesian statistical models in R, but compiling them using C++ for speed [23]. The code is available at https://github.com/ChristelFaes/GrowthModels.

### Prediction of hospital load

The hospital load with respect to the number of hospital beds occupied by COVID-19 patients and the number of COVID-19 patients in ICUs depends on the number of new hospitalisations, the length of stay in the hospital and ICU and the probability to require intensive care.

Let 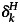 and 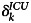 denote, respectively, the probability that a patient is on day *k* after hospitalisation still in the hospital or in the ICU. The number of required hospital beds (*H*(*t*)) can then be estimated as
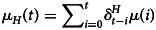
while the number of ICU beds (ICU(*t*)) is calculated as
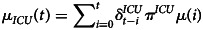
with *π*^*ICU*^ the probability that a hospitalised patient needs intensive care (which is assumed to be constant) and *μ*(.) the mean number of new hospitalisations as specified in Section ‘Growth curve models’. If the distribution of length of stay (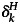 and 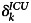) and the probability to require intensive care (*π*^*ICU*^) are known, the number of hospital beds and ICU beds can be directly obtained from the posterior and predictive distribution on the number of new hospitalisations, from which both point estimate and uncertainty bands can be obtained. Availability of the whole predictive distribution allows us to compute the probability of exceeding certain hospital loads, which is of importance for policy makers. For common and well-known diseases, the hospital length of stay is indeed well known, allowing to directly compute the hospital load. For the COVID-19 pandemic however, only very limited information was available in the first half of 2020, requiring additional analyses.

When information on hospital care is limited, a joint model of the number of new hospitalisations (*Y*(*t*)), total number of patients in the hospital (*H*(*t*)) and number of patients in the intensive care (*ICU*(*t*)) can be used to estimate these parameters. This multivariate model can be formulated as
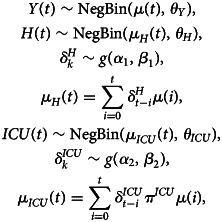
where *μ*(*t*) is described by one of the growth models as given in Section ‘Growth curve models’ and *g*(.) is a distribution for a time to event outcome, such as a gamma, Weibull or lognormal distribution with parameters *α* and *β*. The parameters *θ*_*Y*_, *θ*_*H*_ and *θ*_*ICU*_ are overdispersion parameters corresponding to each of these processes. The different components of the model share the mean growth rate function *μ*(*t*). The advantage of the shared function is that the growth function is less impacted by the heterogeneity in the individual time series, and both the length of stay and probability of requiring intensive care are jointly estimated. The intensive care patients in hospitals is actually modelled as a thinned Poisson negative binomial process of the newly hospitalised patients.

Estimation is done in the Bayesian framework, with the use of vague priors for each of the parameters in the model. The same priors are used as in the univariate model, with the addition of the following priors: a *U*(0, 50) for the overdispersion parameters *θ*_*Y*_, *θ*_*H*_ and *θ*_*ICU*_, a *N*(2.069, 1) and *N*(2.101, 1) for *α*_1_ and *α*_2_ (in line with the information from the individual hospital survey [24]) and *U*(0, 10) for the parameters *β*_1_ and *β*_2_. A sensitivity analysis of the priors was performed to investigate the impact of the priors.

## Real-time analysis

In this section, an illustration is given of the real-time forecast of the number of new hospitalisations and derived hospital load. Also important characteristics of the epidemic such as the turning point and final size are derived. Section ‘Forecasting new hospitalisations at different phases in the epidemic’ focuses on the growth models of new hospitalisations using the univariate models described in Section ‘Growth curve models’. Section ‘Forecasting the hospital load’ incorporates the hospital load using the multivariate model as described in Section ‘Prediction of hospital load’.

### Forecasting new hospitalisations at different phases in the epidemic

We first illustrate the use of the different growth phase models at different phases of the epidemic. We present the forecasts based on the historical data. We perform a 5-day, 7-day and 10-day ahead forecast at different time points, in which data are used from the start of the epidemic until the time point of prediction, and compare the forecasts with the real data obtained for the forecasting period. To assess the forecasting performance, we visually assess the forecast, and calculate several measures of the predictive performance: the root mean squared error (RMSE), the mean absolute percentage error (MAPE), the symmetric mean absolute percentage error (sMAPE), the 95% coverage of the PI and the mean interval score (MIS). The measures for the number of new hospitalisations are defined as
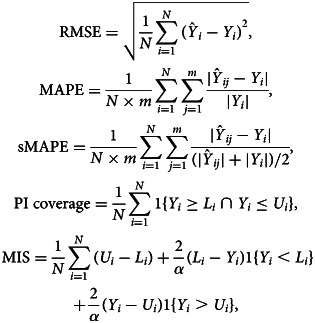
with *Y*_*i*_ the observed number of hospitalisations and 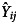 the *j*th MCMC sample of the posterior predictive number. *m* is the number of samples and *N* is the prediction sample size. *L*_*i*_ and *U*_*i*_ are, respectively, the lower and upper limits of the 95% PI, *α* = 0.05 is the significance level, 1 is an indicator with value 1 if the condition is satisfied or 0 otherwise. These are defined similarly for the number of hospital beds and ICU beds. While the RMSE is commonly used to assess the predictive performance, it has the disadvantage that it depends on the size of the epidemic, i.e. larger cases will tend to result in larger RMSE [25]. The MAPE and sMAPE are independent from the scale of the epidemic. The sMAPE has an attractive interpretation with values between 0 and 2, with smaller values corresponding to more accurate prediction. With the same PI coverage, the model with the smaller MIS has less uncertainty [12]. In addition, goodness-of-fit of models are compared using WAIC, with smaller values (difference larger than 5) corresponding to a better fitting model [26, 27].

**Fig. 3.**
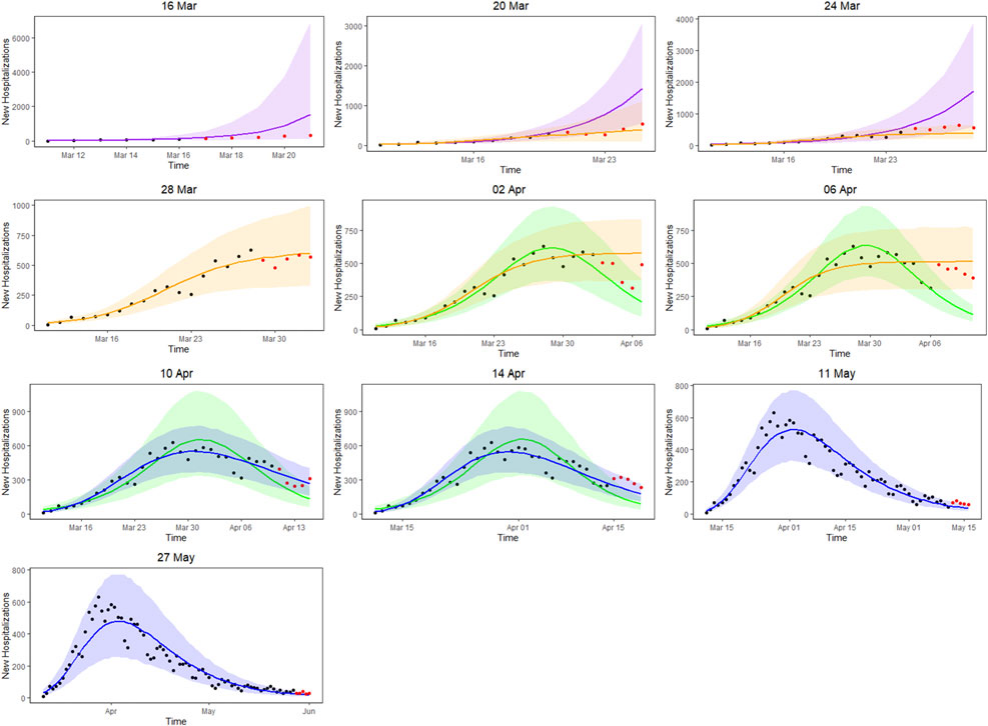
5-day ahead prediction for the number of new COVID-19 hospitalisations. The dots are observed data, where black and red ones correspond to calibration and prediction period, respectively. The line and envelope are posterior mean and 95% CI for models from phase 1 (purple), phase 2 (orange), phase 3 (green) and phase 4 (blue).

**Fig. 4.**
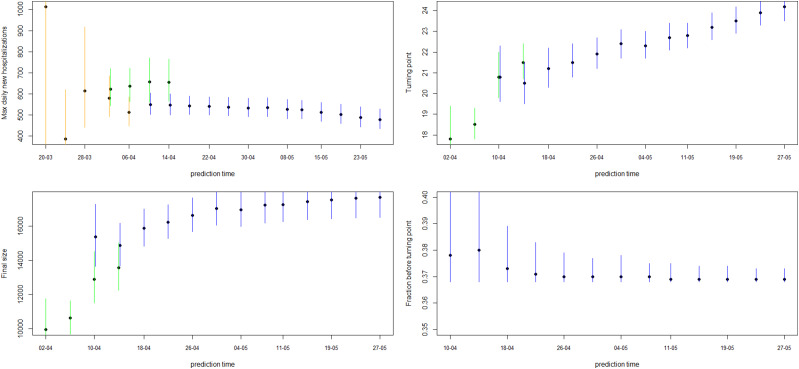
Model estimates for maximum daily new hospitalisations, turning point, final size and fraction before turning point. The dot and line are posterior mean and 95% CI for models from phase 2 (orange), phase 3 (green) and phase 4 (blue).

Figure 3 presents the 5-day ahead predictions for the number of new hospitalisations at different phases of the epidemic. The dots are observed data, where black and red ones correspond to calibration and prediction period, respectively. The line and envelope are posterior mean and 95% confidence interval (CI) for models from phase 1 (exponential growth model, purple), phase 2 (logistic growth model, orange), phase 3 (logistic distribution model, green) and phase 4 (Richards model, blue). Table 2 summarises the overall goodness of fit and prediction performance of 5-, 7- and 10-day ahead prediction using WAIC and sMAPE. The other measures of predictive performance are provided in Appendix Tables 13–16. The parameter estimates of the models are available in Appendix B and visualised in Figure 4.

**Table 2.** Model goodness of fit and prediction performance via sMAPE for the COVID pandemic in Belgium from March to June 2020

			sMAPE (new hosp)			sMAPE (patients in hosp)			sMAPE (patients in ICU)		
Date	Phase	WAIC	5-day	7-day	10-day	5-day	7-day	10-day	5-day	7-day	10-day
16 Mar	*P*_1_	60.1	0.67	0.83	0.99	0.43	0.55	0.72	0.50	0.62	0.79
20 Mar	*P*_1_	101.5	0.70	0.82	1.02	0.31	0.42	0.60	0.41	0.51	0.70
	*P*_2_	103.4	0.44	0.49	0.54	0.26	0.32	0.39	0.21	0.26	0.33
24 Mar	*P*_1_	152.8	0.60	0.78	0.97	0.24	0.34	0.53	0.31	0.42	0.60
	*P*_2_	145.2	0.45	0.43	0.43	0.35	0.38	0.41	0.24	0.27	0.30
28 Mar	*P*_2_	195.4	0.21	0.22	0.29	0.17	0.16	0.15	0.10	0.10	0.11
2 Apr	*P*_2_	250.4	0.32	0.29	0.34	0.10	0.09	0.09	0.07	0.08	0.11
	*P*_3_	258.3	0.42	0.59	0.78	0.21	0.25	0.32	0.13	0.17	0.23
6 Apr	*P*_2_	304.3	0.22	0.34	0.39	0.09	0.08	0.08	0.07	0.08	0.11
	*P*_3_	301.5	0.88	0.92	1.08	0.28	0.34	0.42	0.18	0.23	0.30
10 Apr	*P*_3_	367.8	0.50	0.66	0.80	0.20	0.23	0.30	0.10	0.12	0.17
	*P*_4_	341.2	0.22	0.24	0.25	0.16	0.17	0.19	0.05	0.06	0.07
14 Apr	*P*_3_	413.6	0.84	0.91	1.04	0.26	0.31	0.37	0.12	0.16	0.21
	*P*_4_	383.6	0.33	0.34	0.41	0.22	0.24	0.27	0.08	0.10	0.12
11 May	*P*_4_	658.7	0.51	0.49	0.59	0.26	0.28	0.31	0.05	0.05	0.05
27 May	*P*_4_	823.1	0.48	0.51	0.58	0.23	0.26	0.28	0.12	0.11	0.10

As a starting point, the Poisson and negative binomial distributions were compared in the first phase of the pandemic. The negative binomial model consistently showed a better goodness of fit as compared to the Poisson model (results in Appendix A). This is to be expected as the number of new hospitalisations shows a considerable amount of variation. Therefore, all models presented are based on the negative binomial distribution.

In the initial phase of the outbreak (phase 1), the exponential model was considered. This is illustrated for predictions early in the epidemic on 16 and 20 March (mean curve presented by purple line, PI by purple shaded area). At this initial stage of the epidemic, only 6 and 10 data points are available respectively, and the main question of interest is to get an estimation of the doubling time. It was estimated as 1.967 (1.042–4.068) on 16 March and 2.386 (1.884–3.025) on 20 March. While the estimation of epidemic is consistent with the data, the prediction of the number of future cases at this point is still highly uncertain, leading to wide prediction bands. With an increased amount of data in the calibration phase, the prediction bands become narrower, but the overestimation in the prediction becomes more obvious. This is to be expected as multiple restriction measures (closing of schools, cafes and restaurants) went into effect after 13 March (and were further enforced on 23 March), resulting in a major decrease of the number of social contacts. A comparison with the logistic model from phase 2 is therefore made (orange curves), resulting in a best and worst case scenario prediction.

During the transition from phase 1 to phase 2, goodness-of-fit for both models are similar (as observed by WAIC on 20 March), although prediction is better based on the phase 2 model (as observed by the sMAPE). The same conclusion can be made based on RMSE and MAPE. Note that the RMSE varies a lot from day to day, due to it being impacted by the size of the epidemic at the time of prediction. The prediction bands show good coverage for the selected model (Table 15 in the appendix) and the smallest MIS (Table 16 in the appendix). Moving further in time (as from 24 March), the phase 2 model outperforms the exponential phase 1 model also in WAIC.

Once the stabilisation of new hospitalisations was reached, the phase 2 model was compared to the phase 3 model (green curve), also allowing to estimate the peak of the epidemic. The transition phase is again first marked by the similarity amongst WAIC values, with a worst and best case scenario prediction, and followed by improved predictions in phase 3 in terms of sMAPE on 10 April. The same is concluded from the PI coverage and MIS, indicating that the phase 3 model is outperforming the phase 2 model as from 10 April. As the epidemic evolves, more data points become available allowing for more flexible models. It becomes more and more apparent that the decline is slower as compared to the epidemic increase. The Richards model fits the data well and provides good forecasts for a short-term period.

Overall, the 5-day ahead predictions have better predictive performances as compared to the 7- and 10-day predictions, indicating that short prediction periods are to be recommended. However, for the Richards model, the predictive performance of the 5-, 7- and 10-day forecast are very alike, indicating that at this point somewhat longer forecasts can safely be performed. Longer forecasting horizons were not investigated in this setting, as these models assume that there are no behavioural changes that would impact the growth of the epidemic.

Figure 4 shows how the peak (maximal daily number of new hospitalisations and the turning point), the final size and the fraction before (or after) turning point are estimated through time. The point estimate and credible interval, estimated at different time points and using different models, are presented by the dots and vertical lines. It is clear that estimation of the peak, before the peak is reached, is very difficult, leading to unstable estimates. But, fairly stable estimates are obtained after the turning point. The final size of the first wave is already stable as from the end of April, as well as the amount of new hospitalisations to come after the turning point.

### Forecasting the hospital load

In this section, we estimate the hospital load based on the different growth models. As explained before, two methods can be used for this: (1) by derivation of hospital load based on the univariate model of the new hospitalisations and knowledge about the length of stay in hospital and (2) by joint modelling of the number of new hospitalisations, total hospital beds and total patients in intensive care. Especially in the beginning of the COVID-19 epidemic, no detailed information was available on the length of stay in hospital. Therefore, we present the joint model in this section. Note that during the epidemic, information was collected on the length of stay in hospital and in the Appendix, we use this information, in retrospect, to illustrate the univariate model for a setting in which information would be available. Table 2 presents the sMAPE for the number of patients hospitalised and patients in ICU based on this univariate model. The other predictive measures are given in the Appendix. Predictions of the number of patients in the hospital and in the ICU, based on the univariate model, are provided in Appendix B.

**Fig. 5.**
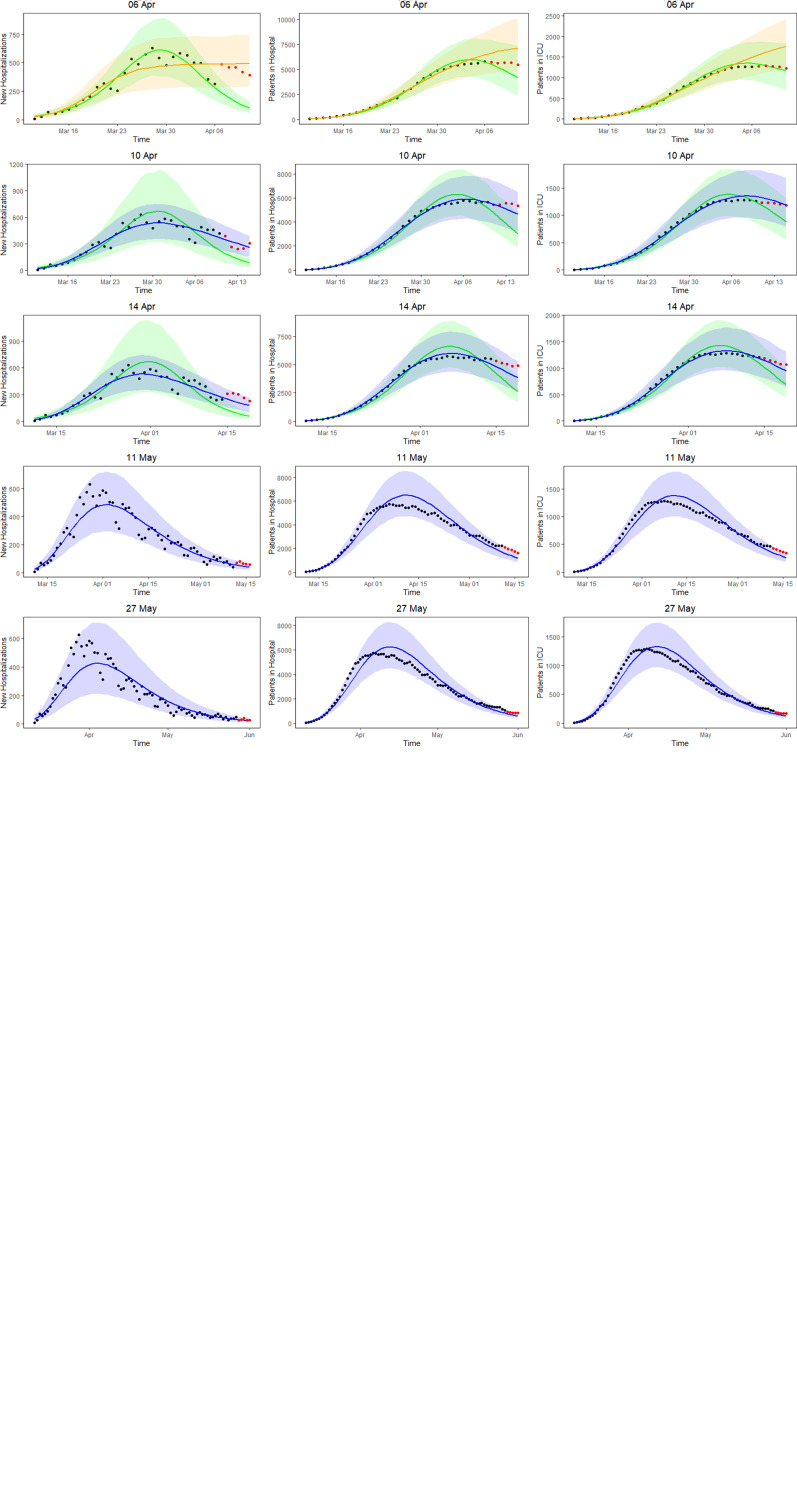
5-day ahead prediction for the number of new COVID-19 hospitalisations, patients in the hospital and patients in the ICU from the joint process. The dots are observed data, where black and red ones are corresponding to calibration and prediction period, respectively. The line and envelope are model fitted line and 95% CI from phase 1 (purple), phase 2 (orange), phase 3 (green) and phase 4 (blue). Column correspond to new hospitalisations (left), total number of patients in the hospital (middle) and number of patients in the ICU (right). Rows correspond to different prediction dates during the epidemic.

Figure 5 shows the 5-day ahead prediction for the number of new COVID-19 hospitalisations, patients in the hospital and patients in the ICU, based on the joint model, using lognormal distributions for the length of stay in the hospital and in the ICU. The dots are observed data, where black and red ones correspond to calibration and prediction period, respectively. The line and envelope are posterior mean and 95% CI for models from phase 1 (purple), phase 2 (orange), phase 3 (green) and phase 4 (blue). Columns correspond to new hospitalisations (left), total number of patients in the hospital (middle) and number of patients in the ICU (right). Rows correspond to different prediction dates during the epidemic.

Similar conclusions with respect to the use of the different models at different time points can be made as based on the univariate model in Section ‘Forecasting new hospitalisations at different phases in the epidemic’. Comparing the left column of Figure 5 (prediction of new hospitalisation based on the joint model) with Figure 3 (prediction of new hospitalisation based on the univariate model), we observe that results are very much alike. Depending on the amount of information available about the ongoing epidemic, different models are better suited to fit the data at hand and to make short-term predictions. When models perform similar in terms of WAIC, they are best used as best and worst case scenario's that can occur. When the WAIC is smaller for the next phase, it is best to switch to the next phase model. This is also confirmed by the sMAPE (and equivalently by RMSE and MAPE) and the length and coverage of PI (PIcov and MIS) of the new hospitalisations. This indicates that the WAIC can indeed be used as an early indicator for the predictive behaviour, while the predictive measures can in a practical setting be calculated only after the predictive interval has past.

An advantage of the joint model is that it also provides estimates and predictions for the number of patients in the hospital (middle column of Fig. 5) and number of patients in the ICU (right column of Fig. 5). The models clearly perform very well in predicting the hospital load. The sMAPE of the 5-, 7- and 10-day predictions of patients in the hospital and in the ICU are also presented in Table 3.

**Table 3.** Model goodness of fit and prediction performance via sMAPE for COVID pandemic in Belgium from March to June 2020 from the joint process

			sMAPE (new hosp)			sMAPE (patients in hosp)			sMAPE (patients in ICU)		
Date	Phase	WAIC	5-day	7-day	10-day	5-day	7-day	10-day	5-day	7-day	10-day
16 Mar	*P*_1_	153.57	0.75	0.93	1.11	0.36	0.48	0.69	0.45	0.59	0.77
20 Mar	*P*_1_	273.50	0.66	0.78	0.98	0.30	0.39	0.56	0.33	0.41	0.59
	*P*_2_	272.60	0.38	0.42	0.46	0.25	0.31	0.40	0.25	0.30	0.38
24 Mar	*P*_1_	423.30	0.62	0.79	1.00	0.31	0.42	0.61	0.31	0.42	0.61
	*P*_2_	407.81	0.43	0.41	0.40	0.25	0.28	0.31	0.25	0.28	0.31
28 Mar	*P*_2_	561.09	0.20	0.21	0.28	0.15	0.16	0.19	0.15	0.16	0.18
2 Apr	*P*_2_	755.53	0.29	0.27	0.32	0.21	0.24	0.27	0.21	0.24	0.29
	*P*_3_	767.70	0.49	0.66	0.85	0.17	0.18	0.23	0.16	0.16	0.17
6 Apr	*P*_2_	924.65	0.21	0.32	0.37	0.22	0.23	0.25	0.27	0.30	0.34
	*P*_3_	924.90	0.95	1.00	1.14	0.25	0.32	0.46	0.18	0.21	0.26
10 Apr	*P*_3_	1115.37	0.77	0.92	1.07	0.38	0.48	0.64	0.23	0.28	0.39
	*P*_4_	1067.19	0.22	0.25	0.25	0.16	0.18	0.22	0.15	0.15	0.16
14 Apr	*P*_3_	1293.40	1.06	1.13	1.25	0.42	0.53	0.68	0.31	0.39	0.52
	*P*_4_	1220.31	0.31	0.33	0.39	0.20	0.24	0.30	0.15	0.17	0.21
11 May	*P*_4_	2251.76	0.40	0.37	0.47	0.31	0.34	0.38	0.26	0.29	0.32
27 May	*P*_4_	2837.38	0.35	0.36	0.41	0.26	0.29	0.31	0.23	0.27	0.27

**Fig. 6.**
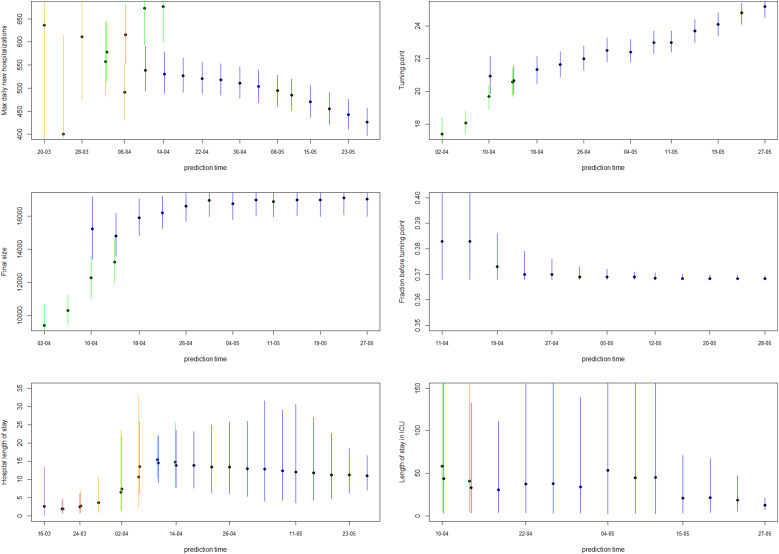
5-day ahead prediction for the number of new COVID-19 hospitalisations, patients in the hospital and patients in the ICU from the joint process. The dots are observed data, where black and red ones are corresponding to calibration and prediction period, respectively. The line and envelope are model fitted line and 95% CI from phase 1 (purple), phase 2 (orange), phase 3 (green) and phase 4 (blue). Column correspond to new hospitalisations (left), total number of patients in the hospital (middle) and number of patients in the ICU (right). Rows correspond to different prediction dates during the epidemic. Model estimates from the joint process for maximum daily new hospitalisations, turning point, final size, fraction before turning point and length of stay in the hospital and ICU. The dots and lines are posterior means and 95% CI for models from phase 1 (purple), phase 2 (orange), phase 3 (green) and phase 4 (blue), respectively.

Estimates of the maximal number of new hospitalisations, the turning point, the final size, the fraction of hospitalisations before the turning point and the length of stay in the hospital and ICU over time are visualised in Figure 6 (and summarised in Appendix C). The maximal number of daily hospitalisations is estimated around 500. This peak is very difficult to predict very early in the epidemic, as can be seen by the large amount of uncertainty and variability at the beginning of the epidemic on these parameters. It is stably estimated as from end of March, which is close to the turning point of the epidemic. This confirms that early prediction of the turning point is not feasible early in the epidemic using phenomenological models. The final size converges to around 17 245 hospitalisations. This is very close to the actual number, as there have been a total of 17 388 hospitalisations between 11 March and 1 June. Already on 10 April, just after the peak, the fraction of hospitalisations before the turning points was estimated as 38% (37–41%). This estimate is very stable, and gives an early prediction of the amount of hospitalisations to still follow after the peak. The distribution of length of stay in the hospital and ICU was estimated in this joint model using a lognormal distribution. The figures show the estimated mean length of stay, together with the 2.5% and 97.5% quantiles of the length of stay distribution. The mean hospital length of stay is estimated to be around 15 days, while the average ICU length of stay is estimated to be higher at around 30 days. However, a very large amount of patient variability is observed for the ICU length of stay. As a sensitivity analysis, a gamma distribution was assumed as well, which provided very similar results (results are given in Appendix D).

**Fig. 7.**
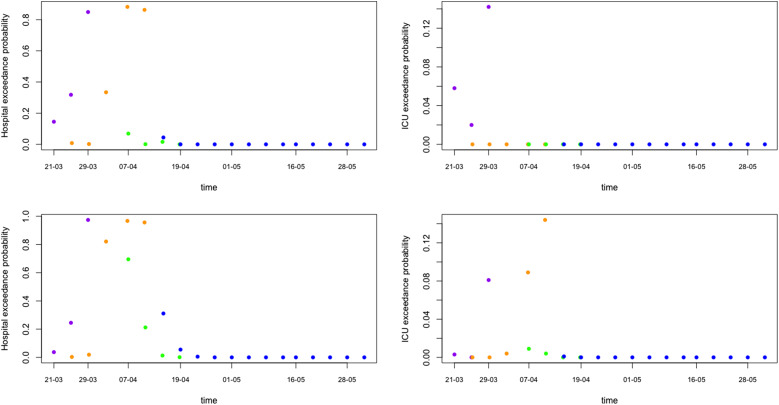
Model prediction for hospital and ICU exceedance probability on day 5 ahead from univariate process (top) and joint process (bottom). The dots are posterior means for models from phase 1 (purple), phase 2 (orange), phase 3 (green) and phase 4 (blue), respectively.

Of major importance for health authorities is to know whether the required hospital capacity will be above the available hospital capacity. In Belgium, initial interest was whether or not the number of patients in hospital would exceed 5000 and in ICU exceed 2100. Such threshold probabilities can be easily obtained from simulations from the predictive distributions (Fig. 7 shows the exceedance probability in 5 days from the prediction date). This is an important tool that can be used as an early warning tool.

## Discussion

During the COVID-19 pandemic, the use of phenomenological models for short-term prediction of the epidemic curve and/or derivation of characteristics of the epidemic wave has become very popular [12, 25, 28, 29]. In this paper, we illustrate how simple phenomenological models such as the exponential model, logistic growth model and Richards model can be used to fit the daily number of new hospitalisations from epidemic outbreaks in a Bayesian framework. Recent developments in MCMC algorithms facilitate the implementation of these Bayesian analyses. The advantage of the Bayesian framework is the ease in which non-linear models are fitted to the data, the simplicity in getting credible intervals and predictive intervals of derived parameters, as well as the possibility to derive the exceedance probabilities. A joint model of the number of new hospitalisations, number of patients in the hospital and in the ICU allows prediction of the hospital load from the epidemic growth model of the new hospitalisations, even without knowledge of the length of stay in the hospital and proportion of patients requiring intensive care. These models enable us to provide short-term predictions, provide worst and best case scenarios, estimate turning points and final sizes of the outbreak, and forecast hospital load. The proposed modelling procedure provides insights into ongoing outbreaks, and uses the available information at different phases of the epidemic. The proposed method facilitates real-time public health responses when faced with infectious disease outbreaks such as COVID-19.

Several alternative phenomenological models exist for the proposed phases of an epidemic outbreak. Chowell *et al*. introduced the generalised logistic and generalised Richards growth model, which includes an additional deceleration of growth parameter and which allows for sub-exponential growth [3]. Li *et al*. show that also other alternative growth curve models can be used for short-term forecasting of COVID-19 cases and that these models can be cast into a Bayesian framework, including the generalised logistic and generalised Richards model, the von Bertalanffy model, Gompertz model and the generalised growth curve model [25]. While some of these models are best suited for phase 3 of the epidemic (due to the property of symmetry), the other models are more suitable for modelling in the phase 4 of the outbreak. In future research, it should be investigated how alternative growth models could be combined within each phase (e.g. using ensemble modelling), to improve the predictive behaviour [11, 25, 30]. This model does not take into account covariates, which can be a constraint. Especially when the epidemic shows an erratic behaviour, due to changes in policy and intervention measures, inclusion of covariates is important. While it is feasible to include covariates in this phenomenological model, alternative methods such as generalised additive models can be useful. Note that the missing covariates can cause extra heterogeneity in the counts, which is taken into account by use of the negative binomial distribution instead of a Poisson distribution. Another approach is to model 3-day or 7-day smoothing averages of the time series [32]. In addition, while in this paper the focus is on modelling data of a single epidemic wave, the development of phenomenological models that allow for multiple epidemic waves is important. Models that deal with successive waves have been proposed [20], and it would be interesting to investigate how such method can be unified with the approach proposed in this paper.

An important assumption in the joint modelling framework when information on the length of stay in the hospital or ICU is not available is that the proportion of patients that receive intensive care is constant. While this assumption is limiting, it is a necessary assumption to allow all parameters to be estimable. Especially when longer time frames are considered, this assumption is probably incorrect; though in such a case information on length of stay might become available. In addition, an important assumption of phenomenological models is that there are no behavioural changes that would impact the growth of the epidemic. Therefore, it is recommended to use these models only for short-term forecasting horizons. The use of individual-based modelling approaches and stochastic compartmental models are recommended for studying the impact policy measures and longer forecasting horizons [2, 31].

## Data Availability

All data used in this paper are available at https://epistat.wiv-isp.be/covid/.

## Supplementary material

For supplementary material accompanying this paper visit https://doi.org/10.1017/S0950268821002491. click here to view supplementary material
