# Supplementary material for: Multivariate phenomenological models for real-time short-term forecasts of hospital capacity for COVID-19 in Belgium from March to June 2020
Source: Epidemiol Infect. 2021 Dec 17;150:e12. doi: 10.1017/S0950268821002491 (PMC8755551; doi:10.1017/S0950268821002491)
Supplement: Supplementary file 1 [file S0950268821002491sup001.zip › Final Version/Appendix.pdf]

Epidemiology and Infection
Multivariate phenomenological models for real-time short-term forecasts of hospital capacity for covid-19 in Belgium from March to June 2020 (*Nguyen, M.H., Braeye, T., Hens, N., Faes, C.*)

Supplementary Material

Appendix A: Poisson versus NegBinomial distribution

For phase 1 starting from 11 March 2020, exponential models with Poisson and Negative Binomial distribution were fitted to the data ending on two different dates. The estimated parameters and model goodness of fit are summarized in Table 1.

Table 1: Model estimates for Phase 1 of COVID19 outbreak in Belgium

Model	End date	$WAIC$	Initial growth	Doubling time
Exponential Poisson	16 Mar	79.5	0.309(0.251-0.373)	2.271(1.859-2.762)
	20 Mar	107.1	0.277(0.252-0.301)	2.511(2.305-2.755)
	24 Mar	274.7	0.187(0.175-0.199)	3.718(3.488-3.958)
Exponential NB	16 Mar	60.1	0.387(0.162-0.647)	1.967(1.042-4.068)
	20 Mar	101.5	0.295(0.229-0.368)	2.386(1.884-3.025)
	24 Mar	152.8	0.225(0.175-0.279)	3.123(2.483-3.951)

The parameter estimates obtained from the exponential Poisson model and the exponential negative binomial model were similar, even though the model with Poisson structure tended to give a lower estimate for the growth rate and hence a higher estimate for the doubling time compared to the model employing negative binomial distribution. With more calibrating data, both model provided a lower estimate for the initial growth rate.

Appendix B: univariate model

In this appendix, we first present results at several additional time points based on the univariate model.

Table 2: *Model prediction performance via SMAPE for the COVID pandemic in Belgium from March to June 2020*

		SMAPE (new hosp)			SMAPE (patients in hosp)			SMAPE (patients in ICU)		
Date	Phase	5-day	7-day	10-day	5-day	7-day	10-day	5-day	7-day	10-day
18 Apr	P_4	0.27	0.32	0.32	0.21	0.21	0.23	0.06	0.07	0.08
22 Apr	P_4	0.27	0.34	0.35	0.21	0.22	0.23	0.08	0.08	0.08
26 Apr	P_4	0.35	0.32	0.35	0.23	0.24	0.26	0.05	0.05	0.05
30 Apr	P_4	0.31	0.34	0.33	0.20	0.21	0.22	0.05	0.05	0.05
04 May	P_4	0.42	0.36	0.44	0.24	0.26	0.28	0.04	0.05	0.06
08 May	P_4	0.36	0.41	0.43	0.23	0.24	0.26	0.05	0.05	0.05
15 May	P_4	0.51	0.52	0.53	0.24	0.26	0.31	0.05	0.05	0.07
19 May	P_4	0.52	0.54	0.58	0.34	0.37	0.37	0.06	0.07	0.06
23 May	P_4	0.48	0.52	0.55	0.33	0.31	0.33	0.05	0.06	0.07

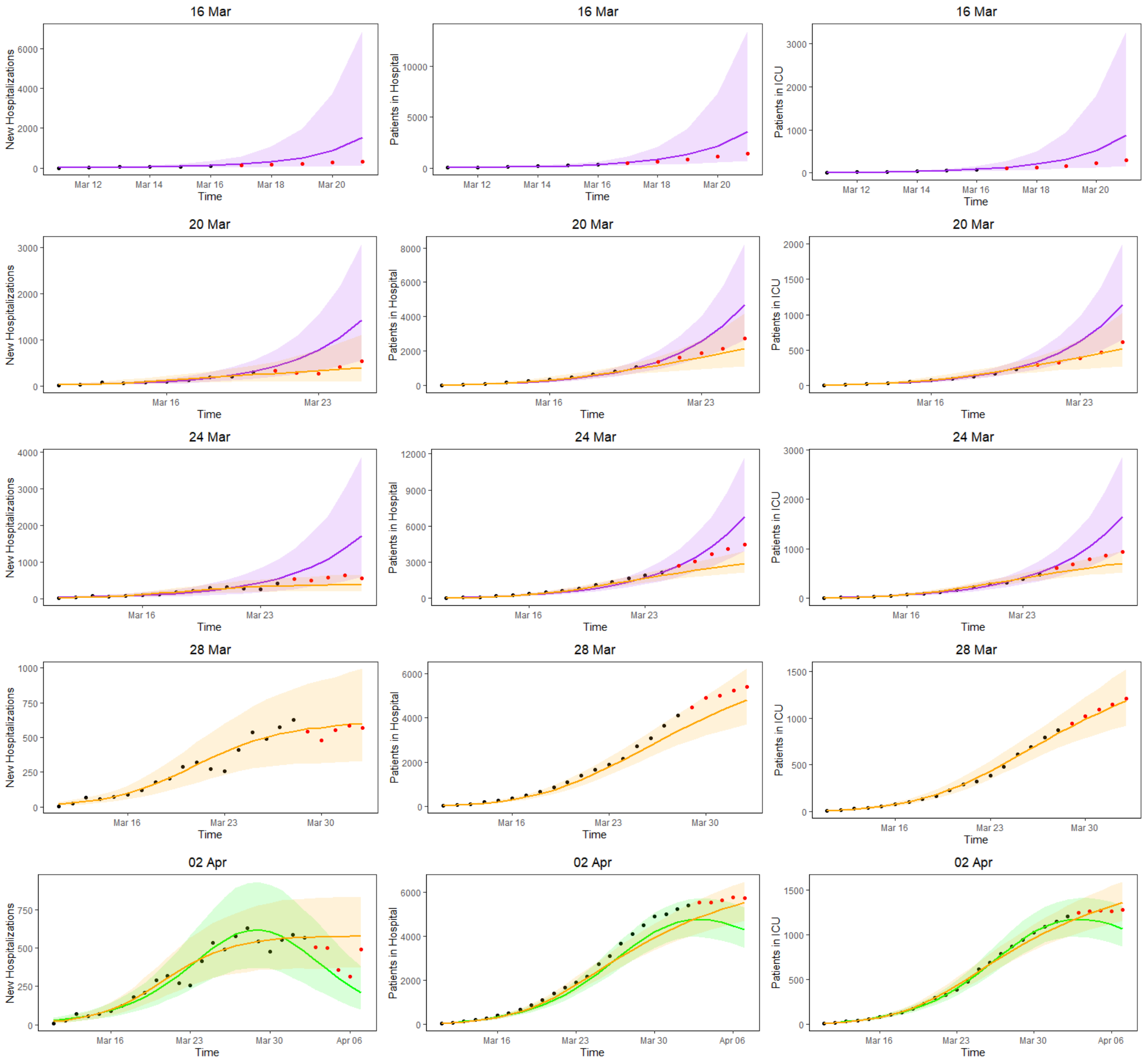

Figure 1: *5-day ahead prediction for number of new covid-19 hospitalization, patients in hospital and patients in ICU. The dots are observed data, where black and red ones correspond to calibration and prediction period, respectively. The line and envelope are posterior mean and 95% CI for models from Phase 1 (purple), Phase 2 (orange), Phase 3 (green) and Phase 4 (blue). Column correspond to new hospitalizations (left), total number of patients in hospital (middle) and number of patients in ICU (right). Rows correspond to different prediction dates during the epidemic.*

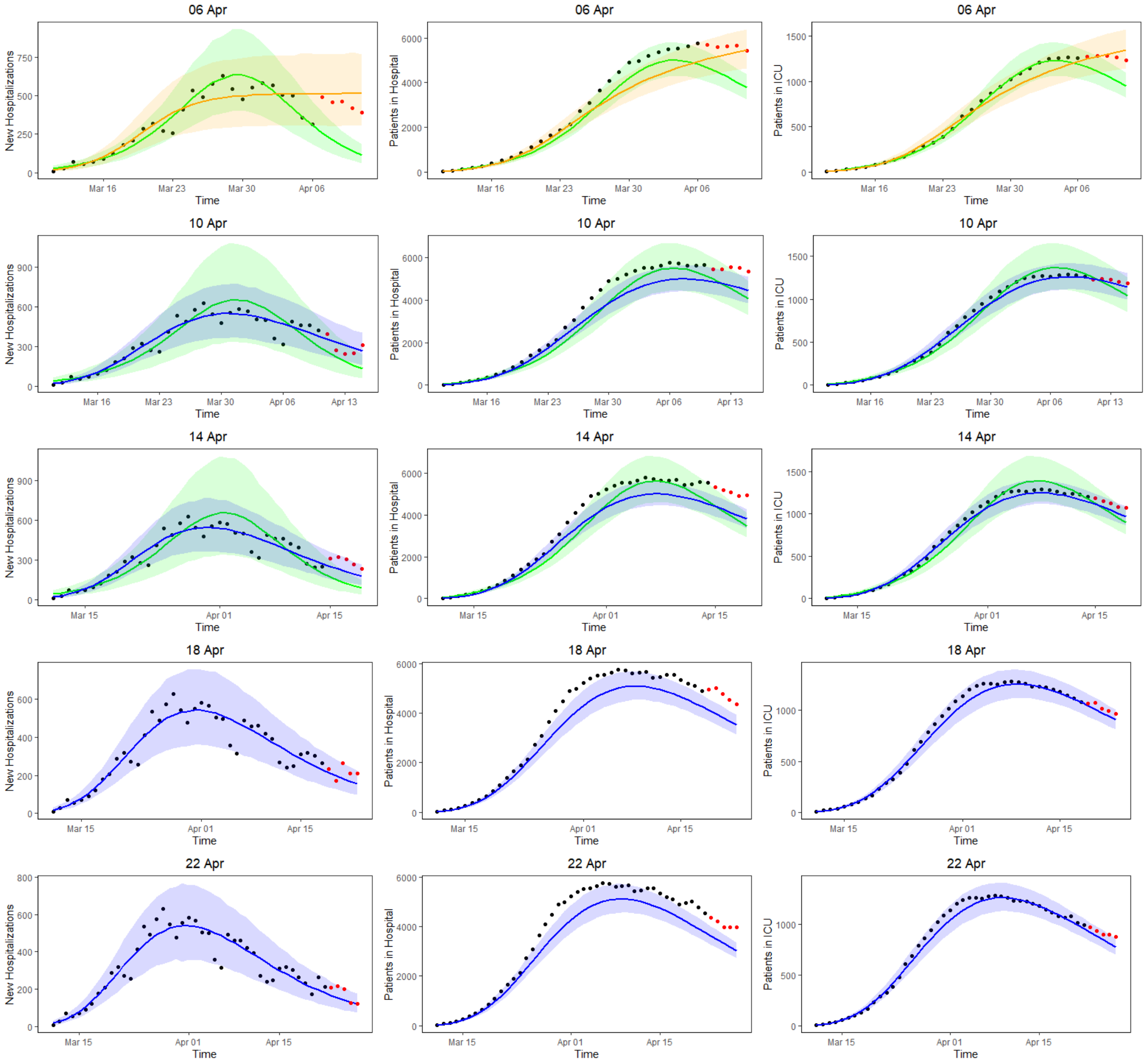

Figure 1: *5-day ahead prediction for number of new covid-19 hospitalization, patients in hospital and patients in ICU. The dots are observed data, where black and red ones correspond to calibration and prediction period, respectively. The line and envelope are posterior mean and 95% CI for models from Phase 1 (purple), Phase 2 (orange), Phase 3 (green) and Phase 4 (blue). Column correspond to new hospitalizations (left), total number of patients in hospital (middle) and number of patients in ICU (right). Rows correspond to different prediction dates during the epidemic.* ***(cont'd)***

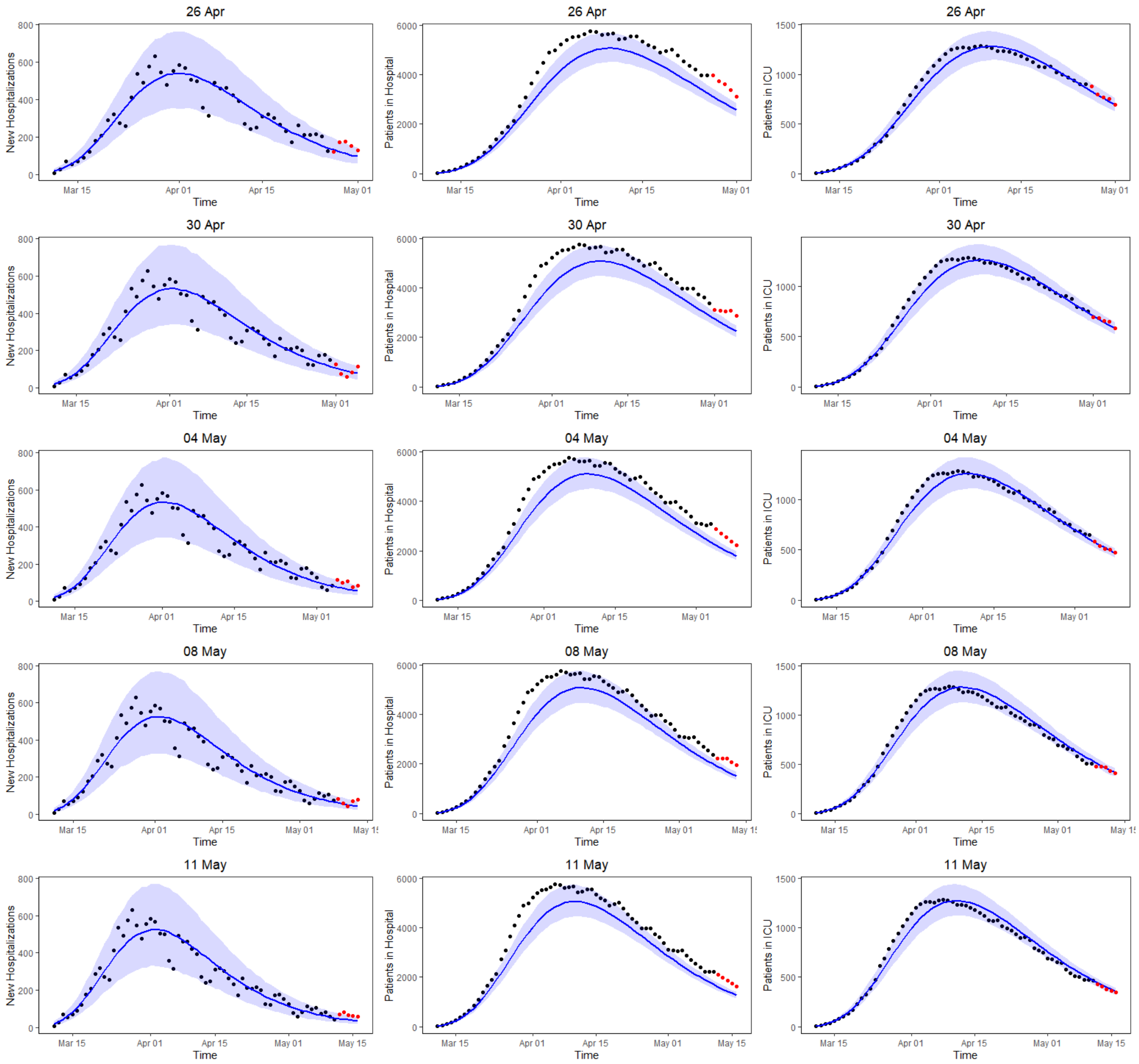

Figure 1: *5-day ahead prediction for number of new covid-19 hospitalization, patients in hospital and patients in ICU. The dots are observed data, where black and red ones correspond to calibration and prediction period, respectively. The line and envelope are posterior mean and 95% CI for models from Phase 1 (purple), Phase 2 (orange), Phase 3 (green) and Phase 4 (blue). Column correspond to new hospitalizations (left), total number of patients in hospital (middle) and number of patients in ICU (right). Rows correspond to different prediction dates during the epidemic.* ***(cont'd)***

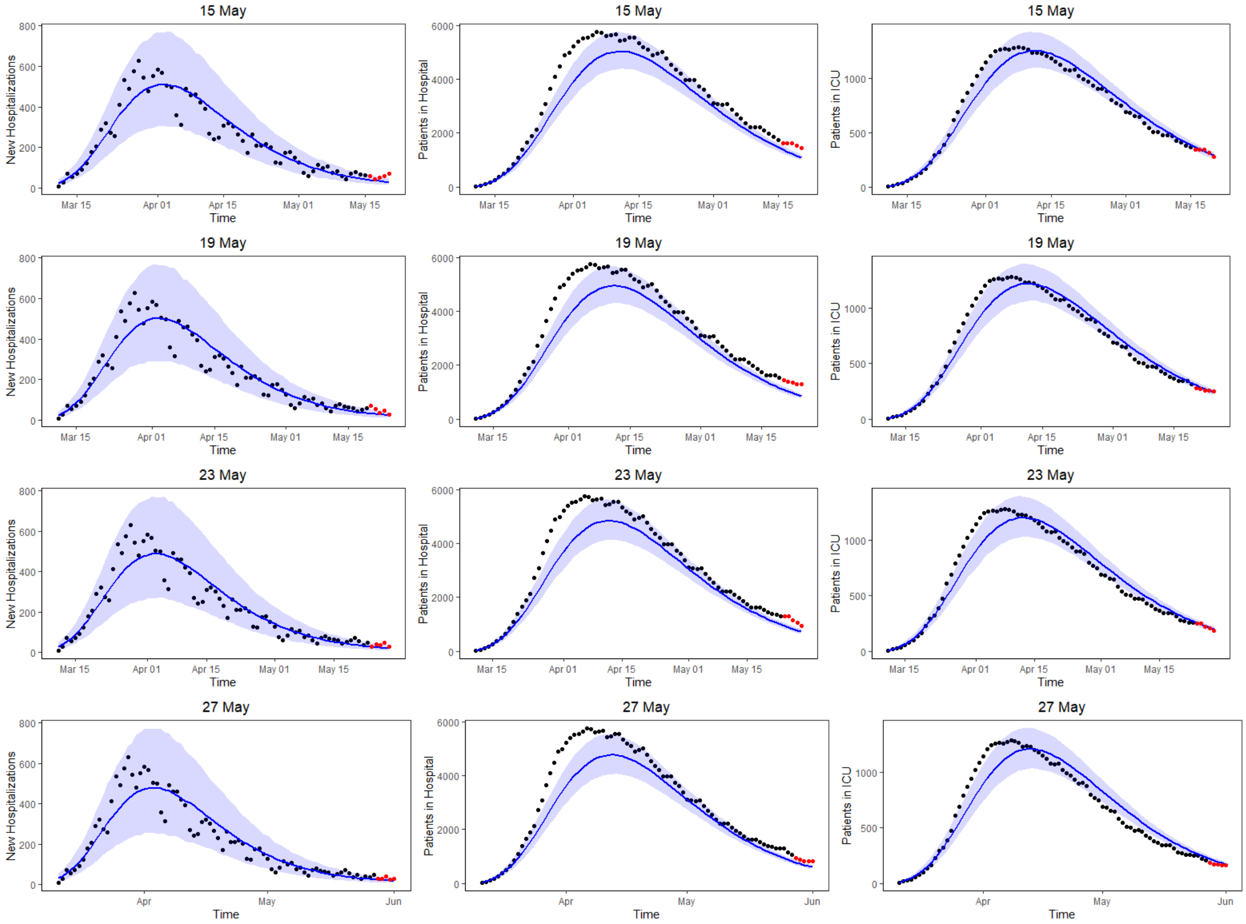

Figure 1: *5-day ahead prediction for number of new covid-19 hospitalization, patients in hospital and patients in ICU. The dots are observed data, where black and red ones correspond to calibration and prediction period, respectively. The line and envelope are posterior mean and 95% CI for models from Phase 1 (purple), Phase 2 (orange), Phase 3 (green) and Phase 4 (blue). Column correspond to new hospitalizations (left), total number of patients in hospital (middle) and number of patients in ICU (right). Rows correspond to different prediction dates during the epidemic.* ***(cont'd)***

Table 3: *Model estimates for common parameters*

End date	Initial growth	Max daily new hospitalisation	Turning point	Final size ($\times 10^4$)
16 Mar$^{(P_1)}$	0.387(0.162-0.647)	-	-	-
20 Mar$^{(P_1)}$	0.295(0.229-0.368)	-	-	-
20 Mar$^{(P_2)}$	0.527(0.285-1.272)	1014.525(143.292-3320.132)	-	-
24 Mar$^{(P_1)}$	0.225(0.175-0.279)	-	-	-
24 Mar$^{(P_2)}$	0.427(0.306-0.590)	386.510(266.561-620.788)	-	-
28 Mar$^{(P_2)}$	0.337(0.261-0.433)	613.878(440.671-917.754)	-	-
02 Apr$^{(P_2)}$	0.339(0.282-0.408)	578.904(493.422-685.021)	-	-
02 Apr$^{(P_3)}$	0.251(0.221-0.281)	622.898(543.177-721.134)	17.835(16.615-19.410)	0.995(0.858-1.175)
06 Apr$^{(P_2)}$	0.371(0.306-0.452)	512.616(448.493-585.083)	-	-
06 Apr$^{(P_3)}$	0.240(0.220-0.262)	637.131(564.921-721.846)	18.477(17.758-19.299)	1.062(0.968-1.163)
10 Apr$^{(P_3)}$	0.204(0.181-0.227)	657.550(559.660-769.993)	20.778(19.770-21.992)	1.289(1.150-1.451)
10 Apr$^{(P_4)}$	54.221(0.530-233.831)	549.986(503.489-604.092)	20.844(19.581-22.299)	1.536(1.363-1.728)
14 Apr$^{(P_3)}$	0.193(0.175-0.213)	655.496(564.755-764.144)	21.520(20.703-22.433)	1.356(1.223-1.501)
14 Apr$^{(P_4)}$	33.878(0.486-210.810)	547.226(501.300-601.177)	20.507(19.530-21.536)	1.487(1.366-1.616)
18 Apr$^{(P_4)}$	76.739(0.853-340.240)	542.956(501.616-590.343)	21.224(20.340-22.179)	1.587(1.483-1.699)
22 Apr$^{(P_4)}$	86.502(1.130-453.494)	540.987(501.181-586.203)	21.540(20.780-22.404)	1.622(1.527-1.726)
26 Apr$^{(P_4)}$	83.357(1.435-486.863)	537.982(496.781-582.722)	21.937(21.203-22.703)	1.662(1.568-1.764)
30 Apr$^{(P_4)}$	110.549(1.769-533.328)	533.027(491.709-579.049)	22.413(21.706-23.155)	1.702(1.605-1.807)
04 May$^{(P_4)}$	92.133(1.675-571.056)	534.730(491.705-580.649)	22.310(21.674-22.991)	1.695(1.596-1.796)
08 May$^{(P_4)}$	110.388(2.019-661.520)	525.886(482.214-573.296)	22.733(22.100-23.393)	1.721(1.618-1.831)
11 May$^{(P_4)}$	166.625(2.025-687.936)	525.002(482.738-569.809)	22.786(22.200-23.421)	1.724(1.624-1.830)
15 May$^{(P_4)}$	139.948(2.397-812.312)	512.199(468.845-560.132)	23.217(22.602-23.867)	1.741(1.636-1.856)
19 May$^{(P_4)}$	128.773(2.452-772.628)	503.339(459.295-551.291)	23.518(22.888-24.192)	1.752(1.641-1.870)
23 May$^{(P_4)}$	178.452(2.509-1079.439)	488.908(443.686-540.405)	23.950(23.261-24.674)	1.763(1.646-1.894)
27 May$^{(P_4)}$	125.427(2.407-760.414)	479.267(434.965-527.702)	24.220(23.504-25.008)	1.768(1.650-1.898)

Table 4: *Model estimates for specific parameters*

End date	Doubling time	Max growth	Time max increase	Fraction before turning point
16 Mar$^{(P_1)}$	1.967(1.042-4.068)	-	-	-
20 Mar$^{(P_1)}$	2.386(1.884-3.025)	-	-	-
20 Mar$^{(P_2)}$	-	89.955(24.069-244.488)	7.162(2.300-16.621)	-
24 Mar$^{(P_1)}$	3.123(2.483-3.951)	-	-	-
24 Mar$^{(P_2)}$	-	40.155(30.775-53.811)	7.234(5.077-10.360)	-
28 Mar$^{(P_2)}$	-	50.827(40.612-65.585)	9.992(7.617-13.132)	-
02 Apr$^{(P_2)}$	-	48.885(41.953-57.089)	9.706(8.293-11.295)	-
06 Apr$^{(P_2)}$	-	47.426(39.768-56.747)	8.798(7.536-10.142)	-
10 Apr$^{(P_4)}$	-	-	-	0.378(0.368-0.406)
14 Apr$^{(P_4)}$	-	-	-	0.380(0.368-0.410)
18 Apr$^{(P_4)}$	-	-	-	0.373(0.368-0.389)
22 Apr$^{(P_4)}$	-	-	-	0.371(0.368-0.383)
26 Apr$^{(P_4)}$	-	-	-	0.370(0.368-0.379)
30 Apr$^{(P_4)}$	-	-	-	0.370(0.368-0.377)
04 May$^{(P_4)}$	-	-	-	0.370(0.368-0.378)
08 May$^{(P_4)}$	-	-	-	0.370(0.368-0.375)
11 May$^{(P_4)}$	-	-	-	0.369(0.368-0.375)
15 May$^{(P_4)}$	-	-	-	0.369(0.368-0.374)
19 May$^{(P_4)}$	-	-	-	0.369(0.368-0.374)
23 May$^{(P_4)}$	-	-	-	0.369(0.368-0.373)
27 May$^{(P_4)}$	-	-	-	0.369(0.368-0.373)

In the above analysis, we derive the hospital load based on the univariate growth models that were fitted. We assume that we know the distribution of length of stay in hospital and in intensive care unit, as well as the proportion of hospitalized patients that require intensive care.

In Belgium, a multicenter hospital clinical survey is being conducted to collect individual information on hospital admission related to covid-19 infection. This database contains information on the length of stay for patients in hospital, and in intensive care unit if requiring intensive care (Van Goethem et al, 2020). Based on this survey, Faes et al. (2020) describe the length of stay in hospital during the first wave. On June 2, 2011, the survey contained information on the length of stay from 12,423 hospitalized covid-19 patients, which have a mean and median LoS of 11.3 and 8 days, respectively. Using the methodology presented by Faes et al. (2020), we described this distribution of length of stay in hospital by a lognormal distribution with parameters (2.069,1.002) and in ICU by a lognormal distribution with parameters (2.101,1.075).

We used this information, in retrospect, to estimate the hospital capacity, based on modeling the new hospitalizations. Results are compared with the actual number of patients in hospital during both the calibration and forecasting period. Note that the information on the number of patients in hospital is not used anywhere in estimation of the hospital capacity. Overall, we find relative good performance of the method in estimation of the hospital capacity trend. However, especially at later phases, deviation from the observed curve are observed, and predictions are somewhat underestimating the actual hospital capacity.

Of major importance for health authorities is to know whether the required hospital capacity is above the available hospital capacity. In Belgium, initial interest was whether or not the number of patients in hospital would exceed 5000. Such threshold probabilities can be easily obtained from simulations from the predictive distributions.

Appendix C: Joint model

Results at additional time points

Table 5: *Model prediction performance via SMAPE for the COVID pandemic in Belgium from March to June 2020 from the joint process*

		SMAPE (new hosp)			SMAPE (patients in hosp)			SMAPE (patients in ICU)		
Date	Phase	5-day	7-day	10-day	5-day	7-day	10-day	5-day	7-day	10-day
18 Apr	P_4	0.25	0.29	0.29	0.23	0.25	0.31	0.17	0.19	0.23
22 Apr	P_4	0.25	0.31	0.31	0.23	0.26	0.30	0.21	0.22	0.25
26 Apr	P_4	0.31	0.31	0.32	0.26	0.28	0.34	0.20	0.23	0.25
30 Apr	P_4	0.31	0.32	0.30	0.27	0.29	0.32	0.21	0.22	0.25
04 May	P_4	0.34	0.31	0.37	0.28	0.31	0.36	0.20	0.24	0.27
08 May	P_4	0.32	0.35	0.36	0.30	0.32	0.36	0.26	0.27	0.31
15 May	P_4	0.40	0.40	0.41	0.32	0.36	0.41	0.28	0.29	0.34
19 May	P_4	0.41	0.41	0.43	0.37	0.40	0.41	0.27	0.31	0.32
23 May	P_4	0.37	0.40	0.40	0.37	0.35	0.38	0.29	0.29	0.32

Table 7: *Model estimates for specific parameters from the joint process*

End date	Doubling time	Max growth	Time max increase	Fraction before turning point
16 Mar$^{(P_1)}$	1.844(1.396-2.472)	-	-	-
20 Mar$^{(P_1)}$	2.518(2.232-2.846)	-	-	-
20 Mar$^{(P_2)}$	-	54.199(24.626-229.911)	7.747(3.729-17.313)	-
24 Mar$^{(P_1)}$	3.115(2.838-3.427)	-	-	-
24 Mar$^{(P_2)}$	-	36.637(29.853-47.449)	7.535(5.596-10.409)	-
28 Mar$^{(P_2)}$	-	46.260(38.867-56.045)	10.263(8.528-12.477)	-
02 Apr$^{(P_2)}$	-	43.739(38.762-49.006)	9.619(8.458-10.915)	-
06 Apr$^{(P_2)}$	-	41.710(36.582-47.167)	8.591(7.565-9.612)	-
10 Apr$^{(P_4)}$	-	-	-	0.383(0.368-0.412)
14 Apr$^{(P_4)}$	-	-	-	0.383(0.368-0.406)
18 Apr$^{(P_4)}$	-	-	-	0.373(0.368-0.386)
22 Apr$^{(P_4)}$	-	-	-	0.370(0.368-0.379)
26 Apr$^{(P_4)}$	-	-	-	0.370(0.368-0.376)
30 Apr$^{(P_4)}$	-	-	-	0.369(0.368-0.373)
04 May$^{(P_4)}$	-	-	-	0.369(0.368-0.372)
08 May$^{(P_4)}$	-	-	-	0.369(0.368-0.371)
11 May$^{(P_4)}$	-	-	-	0.369(0.368-0.370)
15 May$^{(P_4)}$	-	-	-	0.3684(0.3679-0.3702)
19 May$^{(P_4)}$	-	-	-	0.3683(0.3679-0.3698)
23 May$^{(P_4)}$	-	-	-	0.3683(0.3679-0.3696)
27 May$^{(P_4)}$	-	-	-	0.3682(0.3679-0.3695)

Table 6: *Model estimates for common parameters from the joint process*

End date	Initial growth	Max daily new hospitalisation	Turning point	Final size($\times 10^4$)
16 Mar$^{(P_1)}$	0.383(0.280-0.496)	-	-	-
20 Mar$^{(P_1)}$	0.276(0.243-0.310)	-	-	-
20 Mar$^{(P_2)}$	0.401(0.274-0.594)	635.882(189.562-3347.200)	-	-
24 Mar$^{(P_1)}$	0.223(0.202-0.244)	-	-	-
24 Mar$^{(P_2)}$	0.374(0.294-0.466)	399.843(285.288-615.476)	-	-
28 Mar$^{(P_2)}$	0.305(0.263-0.351)	611.469(472.917-812.823)	-	-
02 Apr$^{(P_2)}$	0.315(0.279-0.354)	556.709(481.804-644.059)	-	-
02 Apr$^{(P_3)}$	0.246(0.229-0.263)	577.696(511.926-645.165)	17.439(16.435-18.498)	0.940(0.822-1.065)
06 Apr$^{(P_2)}$	0.340(0.304-0.382)	491.010(430.313-553.406)	-	-
06 Apr$^{(P_3)}$	0.239(0.225-0.253)	616.069(553.101-681.425)	18.116(17.419-18.789)	1.031(0.944-1.122)
10 Apr$^{(P_3)}$	0.219(0.207-0.231)	672.851(592.640-762.603)	19.731(18.916-20.452)	1.227(1.097-1.361)
10 Apr$^{(P_4)}$	13.331(0.469-70.839)	538.582(493.249-590.354)	20.935(19.851-22.187)	1.522(1.341-1.717)
14 Apr$^{(P_3)}$	0.205(0.195-0.215)	677.515(599.129-775.959)	20.631(19.696-21.405)	1.322(1.192-1.471)
14 Apr$^{(P_4)}$	23.753(0.519-51.889)	530.616(487.818-579.044)	20.677(19.826-21.561)	1.481(1.360-1.617)
18 Apr$^{(P_4)}$	93.976(0.983-301.340)	526.513(491.073-565.023)	21.351(20.476-22.150)	1.589(1.480-1.704)
22 Apr$^{(P_4)}$	101.597(1.523-500.548)	520.726(487.823-556.465)	21.648(20.896-22.427)	1.621(1.524-1.721)
26 Apr$^{(P_4)}$	103.024(1.980-641.304)	517.917(484.912-552.582)	22.056(21.332-22.792)	1.659(1.567-1.758)
30 Apr$^{(P_4)}$	187.026(3.228-930.097)	510.942(477.282-546.007)	22.543(21.845-23.289)	1.694(1.598-1.797)
04 May$^{(P_4)}$	183.203(3.669-970.022)	503.153(468.070-539.374)	22.438(21.768-23.183)	1.674(1.577-1.775)
08 May$^{(P_4)}$	197.552(4.671-1112.019)	493.739(459.958-528.489)	22.964(22.298-23.674)	1.697(1.600-1.797)
11 May$^{(P_4)}$	203.990(5.309-1231.143)	485.163(451.239-520.140)	23.049(22.392-23.737)	1.688(1.594-1.787)
15 May$^{(P_4)}$	258.347(5.900-1326.769)	469.667(436.348-505.004)	23.660(22.964-24.381)	1.698(1.602-1.804)
19 May$^{(P_4)}$	301.383(6.882-1347.297)	454.960(421.486-491.039)	24.128(23.419-24.827)	1.697(1.598-1.807)
23 May$^{(P_4)}$	306.823(7.547-1604.807)	442.049(411.079-475.827)	24.769(24.106-25.405)	1.711(1.607-1.826)
27 May$^{(P_4)}$	294.311(7.703-1490.747)	425.602(396.886-457.230)	25.197(24.548-25.810)	1.702(1.597-1.814)

Table 8: *Model estimates for length of stay from the joint process*

	Patients in hospital		Patients in ICU		
End date	α_1	β_1	α_2	β_2	Fraction to ICU
16 Mar$^{(P_1)}$	0.249(-1.078-1.953)	1.191(0.022-7.838)	2.250(0.375-4.090)	5.568(1.240-9.749)	0.506(0.238-0.878)
20 Mar$^{(P_1)}$	0.537(-0.772-1.410)	0.487(0.017-1.593)	2.161(0.253-4.041)	5.204(0.455-9.749)	0.454(0.245-0.772)
20 Mar$^{(P_2)}$	0.537(-0.771-1.426)	0.550(0.018-1.799)	2.293(0.344-4.098)	4.942(0.688-9.710)	0.477(0.250-0.853)
24 Mar$^{(P_1)}$	0.799(-0.626-1.735)	0.528(0.018-1.778)	2.156(0.263-3.981)	5.469(0.733-9.767)	0.409(0.247-0.588)
24 Mar$^{(P_2)}$	0.863(-0.571-1.801)	0.557(0.021-1.713)	2.254(0.351-3.992)	5.196(1.012-9.739)	0.422(0.252-0.708)
28 Mar$^{(P_2)}$	1.090(-0.479-2.107)	0.653(0.026-2.027)	2.323(0.354-4.073)	4.915(0.385-9.722)	0.401(0.209-0.657)
02 Apr$^{(P_2)}$	1.556(-0.231-2.491)	0.811(0.030-2.429)	2.351(0.348-4.147)	4.689(0.729-9.659)	0.371(0.208-0.614)
02 Apr$^{(P_3)}$	1.768(0.134-2.555)	0.689(0.022-2.202)	2.413(0.424-4.182)	5.120(0.967-9.725)	0.370(0.256-0.534)
06 Apr$^{(P_2)}$	2.135(0.479-2.714)	0.704(0.030-2.538)	2.488(0.716-4.010)	3.750(0.244-9.554)	0.333(0.195-0.518)
06 Apr$^{(P_3)}$	2.539(1.014-2.830)	0.368(0.009-2.122)	3.226(1.514-4.635)	1.908(0.429-8.111)	0.463(0.229-0.914)
10 Apr$^{(P_3)}$	2.722(2.517-2.840)	0.188(0.007-0.586)	3.559(2.745-4.456)	1.009(0.398-1.973)	0.611(0.241-0.971)
10 Apr$^{(P_4)}$	2.649(2.422-2.783)	0.225(0.011-0.602)	3.247(2.383-4.424)	1.033(0.177-2.183)	0.461(0.199-0.903)
14 Apr$^{(P_3)}$	2.656(2.488-2.769)	0.303(0.017-0.721)	3.318(2.587-4.119)	0.885(0.171-1.537)	0.503(0.206-0.950)
14 Apr$^{(P_4)}$	2.595(2.413-2.718)	0.290(0.024-0.616)	3.110(2.388-4.144)	0.894(0.138-1.647)	0.416(0.195-0.869)
18 Apr$^{(P_4)}$	2.592(2.459-2.693)	0.282(0.023-0.562)	3.086(2.427-3.984)	0.817(0.099-1.496)	0.409(0.193-0.833)
22 Apr$^{(P_4)}$	2.538(2.412-2.638)	0.347(0.046-0.632)	3.201(2.393-4.157)	0.927(0.196-1.487)	0.461(0.195-0.922)
26 Apr$^{(P_4)}$	2.515(2.400-2.611)	0.373(0.061-0.654)	3.183(2.402-4.193)	0.955(0.239-1.542)	0.449(0.200-0.899)
30 Apr$^{(P_4)}$	2.486(2.373-2.583)	0.394(0.072-0.684)	3.104(2.367-4.077)	0.920(0.226-1.536)	0.426(0.199-0.889)
04 May$^{(P_4)}$	2.403(2.281-2.508)	0.530(0.207-0.848)	3.284(2.343-4.478)	1.184(0.464-1.888)	0.465(0.210-0.906)
08 May$^{(P_4)}$	2.392(2.274-2.495)	0.499(0.154-0.817)	3.221(2.369-4.274)	1.084(0.406-1.651)	0.466(0.212-0.929)
11 May$^{(P_4)}$	2.347(2.223-2.455)	0.554(0.200-0.902)	3.193(2.280-4.490)	1.119(0.291-1.893)	0.451(0.201-0.934)
15 May$^{(P_4)}$	2.354(2.230-2.465)	0.466(0.075-0.826)	2.745(2.263-3.611)	0.763(0.084-1.408)	0.318(0.197-0.613)
19 May$^{(P_4)}$	2.352(2.229-2.461)	0.396(0.036-0.769)	2.806(2.249-3.941)	0.709(0.040-1.498)	0.351(0.196-0.792)
23 May$^{(P_4)}$	2.377(2.256-2.480)	0.279(0.017-0.629)	2.780(2.312-3.744)	0.544(0.017-1.249)	0.347(0.200-0.783)
27 May$^{(P_4)}$	2.369(2.247-2.471)	0.220(0.011-0.562)	2.523(2.307-3.191)	0.263(0.007-0.947)	0.251(0.202-0.491)

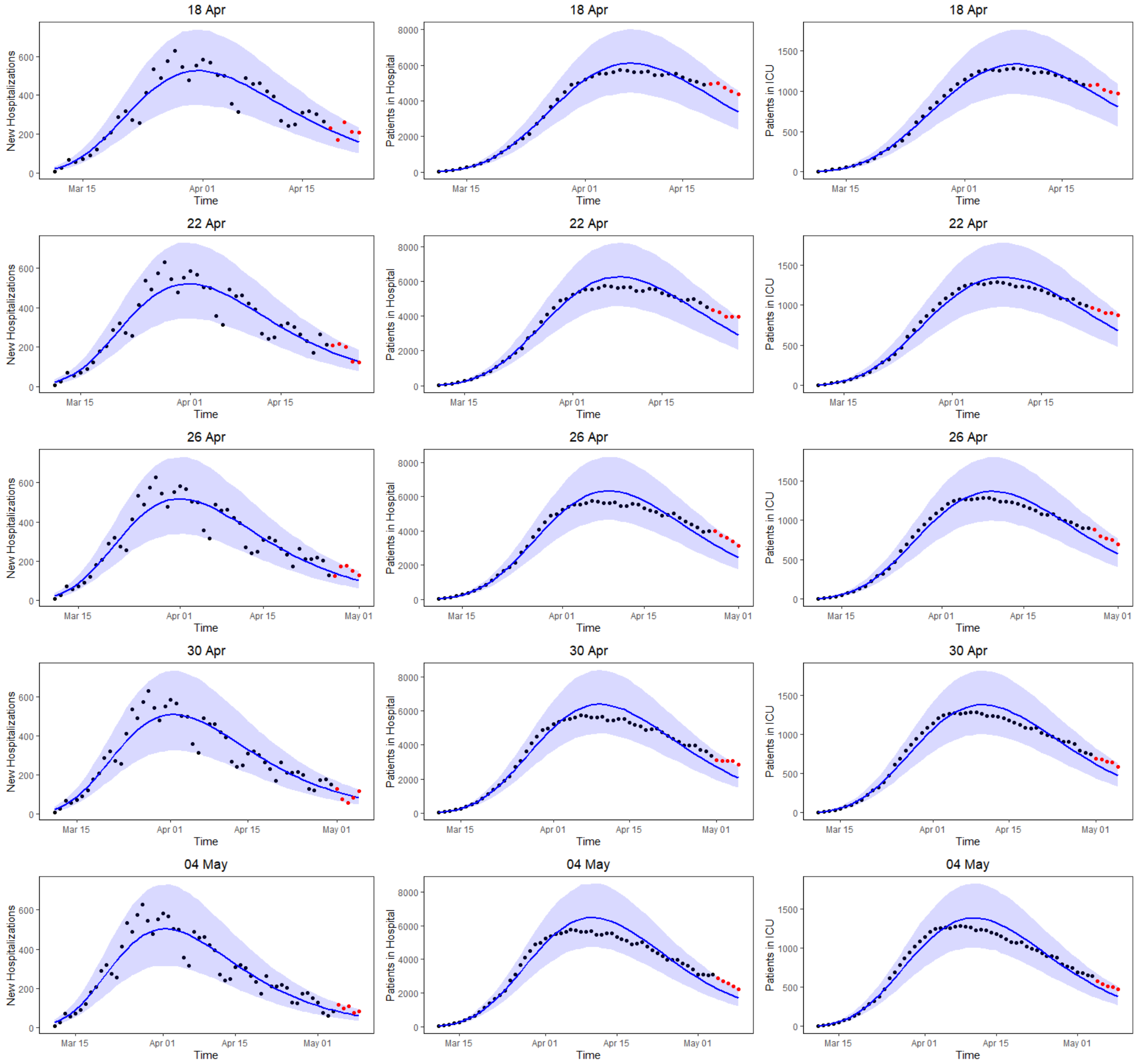

Figure 2: *5-day ahead prediction for number of new covid-19 hospitalization, patients in hospital and patients in ICU from the joint process. The dots are observed data, where black and red ones are corresponding to calibration and prediction period, respectively. The line and envelope are model fitted line and 95%CI from Phase 1 (purple), Phase 2 (orange), Phase 3 (green) and Phase 4 (blue). Column correspond to new hospitalizations (left), total number of patients in hospital (middle) and number of patients in ICU (right). Rows correspond to different prediction dates during the epidemic.*

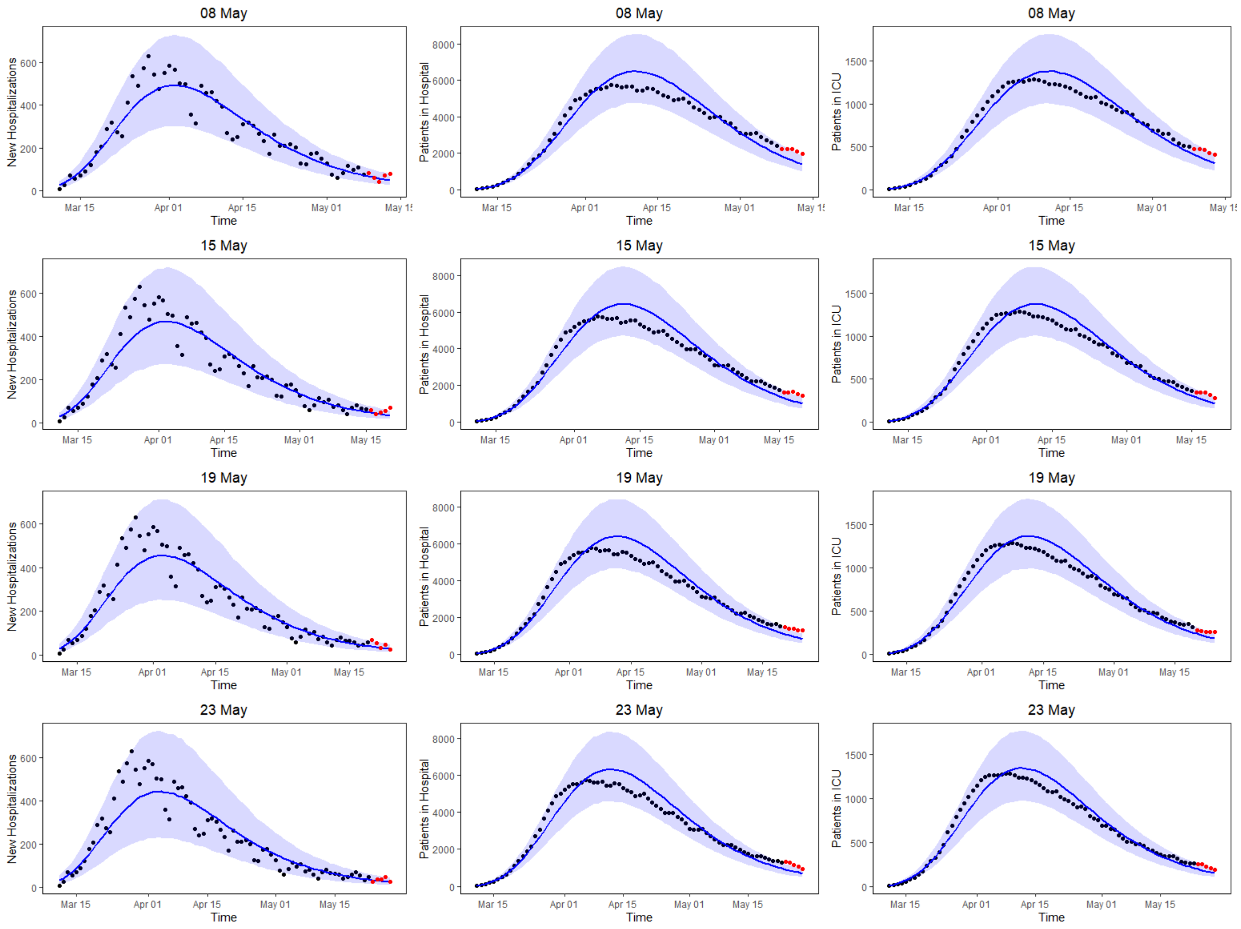

Figure 2: *5-day ahead prediction for number of new covid-19 hospitalization, patients in hospital and patients in ICU from the joint process. The dots are observed data, where black and red ones are corresponding to calibration and prediction period, respectively. The line and envelope are model fitted line and 95%CI from Phase 1 (purple), Phase 2 (orange), Phase 3 (green) and Phase 4 (blue). Column correspond to new hospitalizations (left), total number of patients in hospital (middle) and number of patients in ICU (right). Rows correspond to different prediction dates during the epidemic.* ***(cont'd)***

Sensitivity analysis: gamma distribution for length of stay

Table 9: *Model goodness of fit and prediction performance via RMSE for covid-19 pandemic in Belgium from March to June 2020 from the joint process with gamma distribution*

			RMSE (new hosp)			RMSE (patients in hosp)			RMSE (patients in ICU)		
Date	Phase	$WAIC$	5-day	7-day	10-day	5-day	7-day	10-day	5-day	7-day	10-day
16 Mar	P_1	151.44	370.24	913.67	2821.13	577.08	1619.33	6017.54	141.07	380.19	1336.69
20 Mar	P_1	273.46	480.01	871.32	2112.21	1074.48	2188.84	5980.77	199.20	423.40	1216.79
	P_2	272.78	75.44	78.99	82.57	199.37	411.22	738.56	91.06	150.56	223.73
24 Mar	P_1	423.86	713.10	1217.24	2399.53	1934.64	3722.62	8694.34	377.51	742.28	1738.46
	P_2	408.11	180.31	166.28	163.42	615.55	794.81	950.78	221.13	274.23	347.98
28 Mar	P_2	561.75	49.09	71.27	144.44	550.91	811.48	1162.81	25.06	50.45	111.72
02 Apr	P_2	754.17	155.53	144.27	170.21	1219.15	1450.28	1768.33	178.45	219.47	289.61
	P_3	766.6	172.95	226.21	245.73	491.17	424.55	571.94	52.89	111.65	202.93
06 Apr	P_2	921.67	74.91	150.50	174.22	1195.40	1308.56	1473.29	203.82	235.80	286.70
	P_3	923.38	279.37	255.02	254.39	559.11	964.29	1531.23	187.81	259.57	361.16
10 Apr	P_3	1116.86	166.76	193.74	192.15	1256.82	1584.59	2061.60	270.79	335.21	425.58
	P_4	1067.75	50.96	57.99	52.74	243.94	293.01	510.64	35.50	51.60	94.92
14 Apr	P_3	1291.84	194.66	190.46	188.85	1571.00	1923.82	2220.40	327.67	393.49	463.71
	P_4	1219.81	70.62	72.49	76.31	618.24	840.31	996.76	104.23	142.92	183.53
18 Apr	P_4	1373.47	46.74	53.61	49.90	799.21	838.39	978.82	144.35	163.09	194.92
22 Apr	P_4	1527.82	38.47	46.93	44.18	745.96	829.22	872.00	163.98	169.10	179.79
26 Apr	P_4	1681.57	40.09	36.12	34.29	736.23	769.11	856.09	136.11	145.04	152.51
30 Apr	P_4	1838.35	26.73	27.65	24.33	669.20	695.24	701.50	118.72	115.23	121.36
04 May	P_4	1991.22	26.10	22.38	24.42	589.30	626.35	651.77	90.00	105.17	111.56
08 May	P_4	2139.00	18.06	18.94	17.83	540.22	544.68	556.54	98.73	98.60	104.28
11 May	P_4	2249.50	20.56	18.27	23.09	468.61	491.50	511.06	85.58	93.74	93.56
15 May	P_4	2396.84	19.32	18.50	17.24	426.76	443.15	479.44	78.14	76.07	80.63
19 May	P_4	2543.72	18.30	16.55	16.27	409.25	431.88	405.34	59.55	65.16	62.67
23 May	P_4	2692.36	11.49	12.11	11.31	361.08	329.37	319.21	57.68	53.04	54.35
27 May	P_4	2842.26	7.62	7.79	8.83	188.22	207.98	203.77	33.87	39.23	36.99

Table 10: *Model estimates for common parameters from the joint process with gamma distribution*

End date	Initial growth	Max daily new hospitalisation	Turning point	Final size ($\times 10^4$)
16 Mar$^{(P_1)}$	0.374(0.284-0.469)	-	-	-
20 Mar$^{(P_1)}$	0.279(0.247-0.312)	-	-	-
20 Mar$^{(P_2)}$	0.410(0.277-0.634)	1223.379(181.573-5483.817)	-	-
24 Mar$^{(P_1)}$	0.225(0.203-0.247)	-	-	-
24 Mar$^{(P_2)}$	0.377(0.305-0.454)	402.977(288.803-603.354)	-	-
28 Mar$^{(P_2)}$	0.305(0.262-0.355)	623.611(470.535-860.483)	-	-
02 Apr$^{(P_2)}$	0.313(0.280-0.350)	572.314(488.802-665.131)	-	-
02 Apr$^{(P_3)}$	0.248(0.230-0.266)	590.033(517.903-664.527)	17.499(16.564-18.536)	0.952(0.837-1.085)
06 Apr$^{(P_2)}$	0.337(0.302-0.375)	511.946(447.773-584.301)	-	-
06 Apr$^{(P_3)}$	0.240(0.227-0.253)	628.820(568.414-693.256)	18.122(17.497-18.764)	1.048(0.961-1.138)
10 Apr$^{(P_3)}$	0.221(0.209-0.234)	693.172(605.022-791.551)	19.531(18.824-20.237)	1.253(1.112-1.398)
10 Apr$^{(P_4)}$	22.932(0.559-127.710)	539.018(495.775-587.830)	20.829(19.810-21.965)	1.539(1.368-1.722)
14 Apr$^{(P_3)}$	0.205(0.195-0.215)	686.397(607.443-770.067)	20.630(20.033-21.218)	1.336(1.206-1.472)
14 Apr$^{(P_4)}$	47.600(0.520-102.790)	534.362(493.712-582.470)	20.602(19.831-21.424)	1.494(1.373-1.631)
18 Apr$^{(P_4)}$	65.408(1.002-274.770)	530.981(494.941-569.902)	21.240(20.507-21.978)	1.593(1.486-1.707)
22 Apr$^{(P_4)}$	97.257(1.493-495.017)	524.565(491.439-559.733)	21.633(21.002-22.279)	1.629(1.532-1.731)
26 Apr$^{(P_4)}$	113.077(2.101-633.156)	520.835(488.698-554.320)	22.056(21.417-22.671)	1.667(1.575-1.765)
30 Apr$^{(P_4)}$	167.299(3.695-927.471)	514.378(483.081-547.855)	22.526(21.925-23.132)	1.701(1.609-1.799)
04 May$^{(P_4)}$	152.045(3.630-965.829)	503.855(471.679-537.756)	22.501(21.880-23.106)	1.680(1.588-1.777)
08 May$^{(P_4)}$	211.568(4.674-1085.373)	493.802(462.065-526.143)	23.019(22.434-23.599)	1.699(1.606-1.797)
11 May$^{(P_4)}$	245.709(5.132-1258.294)	486.172(455.075-519.797)	23.109(22.535-23.708)	1.695(1.605-1.795)
15 May$^{(P_4)}$	255.531(6.011-1363.693)	470.225(440.240-502.518)	23.774(23.281-24.296)	1.702(1.605-1.806)
19 May$^{(P_4)}$	261.101(6.857-1517.889)	457.439(428.615-489.397)	24.126(23.615-24.660)	1.702(1.606-1.807)
23 May$^{(P_4)}$	276.654(7.547-1597.209)	451.017(420.746-483.181)	24.539(23.977-25.092)	1.724(1.620-1.835)
27 May$^{(P_4)}$	324.947(8.098-1635.819)	437.171(406.862-469.727)	24.920(24.341-25.488)	1.720(1.614-1.836)

Table 11: *Model estimates for specific parameters from the joint process with gamma distribution*

End date	Doubling time	Max growth	Time max increase	Fraction before turning point
16 Mar$^{(P_1)}$	1.887(1.478-2.444)	-	-	-
20 Mar$^{(P_1)}$	2.495(2.224-2.811)	-	-	-
20 Mar$^{(P_2)}$	-	92.080(24.831-384.441)	7.915(3.571-19.247)	-
24 Mar$^{(P_1)}$	3.084(2.801-3.410)	-	-	-
24 Mar$^{(P_2)}$	-	37.350(29.931-48.578)	7.583(5.774-10.177)	-
28 Mar$^{(P_2)}$	-	47.094(38.759-58.854)	10.388(8.475-12.716)	-
02 Apr$^{(P_2)}$	-	44.728(39.337-50.422)	9.823(8.678-11.059)	-
06 Apr$^{(P_2)}$	-	43.070(37.983-48.638)	8.908(7.914-9.925)	-
10 Apr$^{(P_4)}$	-	-	-	0.379(0.368-0.404)
14 Apr$^{(P_4)}$	-	-	-	0.381(0.368-0.407)
18 Apr$^{(P_4)}$	-	-	-	0.373(0.368-0.385)
22 Apr$^{(P_4)}$	-	-	-	0.371(0.368-0.379)
26 Apr$^{(P_4)}$	-	-	-	0.370(0.368-0.375)
30 Apr$^{(P_4)}$	-	-	-	0.369(0.368-0.372)
04 May$^{(P_4)}$	-	-	-	0.369(0.368-0.372)
08 May$^{(P_4)}$	-	-	-	0.369(0.368-0.371)
11 May$^{(P_4)}$	-	-	-	0.3685(0.3679-0.3707)
15 May$^{(P_4)}$	-	-	-	0.3684(0.3679-0.3702)
19 May$^{(P_4)}$	-	-	-	0.3683(0.3679-0.3698)
23 May$^{(P_4)}$	-	-	-	0.3683(0.3679-0.3696)
27 May$^{(P_4)}$	-	-	-	0.3683(0.3679-0.3694)

Table 12: *Model estimates for length of stay from the joint process with gamma distribution*

	Patients in hospital		Patients in ICU		
End date	α_1	β_1	α_2	β_2	Fraction to ICU
16 Mar$^{(P_1)}$	2.226(0.015-9.043)	1.724(0.023-8.619)	2.046(0.087-7.765)	3.949(0.070-9.653)	0.446(0.184-0.959)
20 Mar$^{(P_1)}$	2.740(0.030-9.320)	1.565(0.031-8.038)	3.469(0.146-9.408)	2.895(0.171-9.171)	0.382(0.196-0.903)
20 Mar$^{(P_2)}$	2.678(0.040-9.198)	1.771(0.030-8.591)	2.244(0.150-8.046)	4.456(0.159-9.736)	0.395(0.191-0.887)
24 Mar$^{(P_1)}$	2.889(0.046-9.293)	1.836(0.050-8.334)	2.755(0.128-9.051)	3.974(0.140-9.576)	0.322(0.189-0.877)
24 Mar$^{(P_2)}$	2.967(0.054-9.347)	1.884(0.053-8.301)	2.649(0.168-8.249)	4.368(0.154-9.693)	0.367(0.188-0.909)
28 Mar$^{(P_2)}$	3.426(0.110-9.400)	2.180(0.085-8.292)	2.802(0.120-8.857)	3.704(0.144-9.566)	0.254(0.188-0.479)
02 Apr$^{(P_2)}$	4.382(0.456-9.602)	2.837(0.539-8.645)	3.243(0.265-9.202)	4.288(0.266-9.633)	0.246(0.182-0.431)
02 Apr$^{(P_3)}$	3.858(0.443-9.404)	3.238(0.622-8.892)	3.442(0.389-9.129)	4.382(0.410-9.646)	0.257(0.185-0.518)
06 Apr$^{(P_2)}$	5.425(1.334-9.720)	2.991(1.276-7.662)	3.394(0.846-8.740)	5.206(1.045-9.631)	0.264(0.182-0.544)
06 Apr$^{(P_3)}$	6.693(2.330-9.812)	2.378(1.427-5.602)	4.153(1.523-9.430)	5.549(1.347-9.835)	0.295(0.188-0.550)
10 Apr$^{(P_3)}$	7.605(3.880-9.897)	2.149(1.504-3.954)	4.153(2.047-9.025)	5.750(1.675-9.748)	0.305(0.192-0.540)
10 Apr$^{(P_4)}$	6.620(2.390-9.849)	2.453(1.419-5.458)	4.789(1.752-9.589)	4.392(1.343-9.612)	0.254(0.185-0.424)
14 Apr$^{(P_3)}$	7.782(4.651-9.889)	2.012(1.459-3.280)	4.937(2.230-9.570)	4.812(1.511-9.786)	0.289(0.190-0.554)
14 Apr$^{(P_4)}$	6.788(3.168-9.816)	2.270(1.415-4.278)	3.662(1.817-8.833)	5.644(1.479-9.808)	0.266(0.191-0.411)
18 Apr$^{(P_4)}$	7.160(3.505-9.855)	2.133(1.390-3.997)	4.315(1.966-9.265)	5.092(1.378-9.723)	0.264(0.191-0.410)
22 Apr$^{(P_4)}$	6.611(3.648-9.766)	2.215(1.376-3.752)	3.896(1.978-8.813)	5.093(1.450-9.713)	0.257(0.194-0.365)
26 Apr$^{(P_4)}$	6.200(2.835-9.731)	2.395(1.357-4.841)	3.374(1.849-7.882)	5.791(1.555-9.801)	0.262(0.196-0.370)
30 Apr$^{(P_4)}$	5.769(2.409-9.539)	2.600(1.350-5.588)	3.128(1.916-6.963)	6.019(1.816-9.817)	0.267(0.201-0.382)
04 May$^{(P_4)}$	4.036(1.673-8.187)	3.754(1.476-8.017)	2.873(1.657-6.027)	5.991(1.916-9.810)	0.246(0.189-0.327)
08 May$^{(P_4)}$	4.781(1.903-9.396)	3.121(1.282-6.873)	2.795(1.727-5.264)	6.142(2.197-9.790)	0.258(0.197-0.348)
11 May$^{(P_4)}$	3.622(1.625-7.921)	3.981(1.470-8.037)	3.408(1.644-8.384)	5.196(1.289-9.736)	0.239(0.188-0.322)
15 May$^{(P_4)}$	5.606(2.792-9.508)	2.339(1.206-4.327)	3.218(1.811-8.171)	5.726(1.438-9.765)	0.266(0.205-0.355)
19 May$^{(P_4)}$	5.676(2.458-9.703)	2.359(1.150-4.884)	4.367(1.953-9.483)	4.300(1.141-9.551)	0.251(0.196-0.357)
23 May$^{(P_4)}$	5.565(2.195-9.690)	2.525(1.152-5.499)	4.045(1.928-9.278)	4.990(1.234-9.705)	0.268(0.199-0.389)
27 May$^{(P_4)}$	5.740(2.132-9.726)	2.407(1.122-5.540)	5.359(2.128-9.683)	3.360(1.159-9.401)	0.250(0.203-0.381)

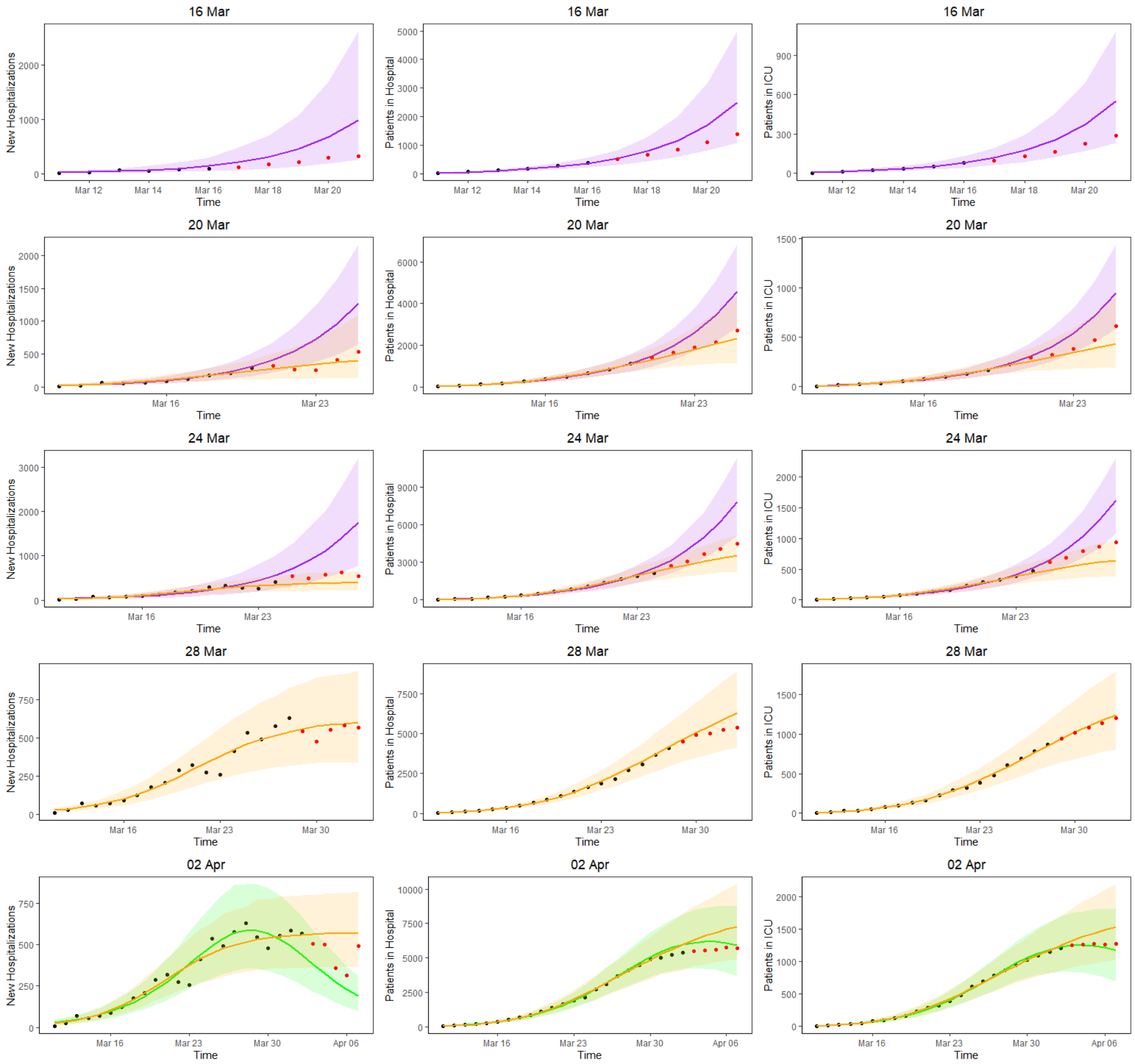

Figure 3: *5-day ahead prediction for number of new covid-19 hospitalization, patients in hospital and patients in ICU from the joint process with gamma distribution. The dots are observed data, where black and red ones correspond to calibration and prediction period, respectively. The line and envelope are posterior mean and 95% CI for models from Phase 1 (purple), Phase 2 (orange), Phase 3 (green) and Phase 4 (blue). Column correspond to new hospitalizations (left), total number of patients in hospital (middle) and number of patients in ICU (right). Rows correspond to different prediction dates during the epidemic.*

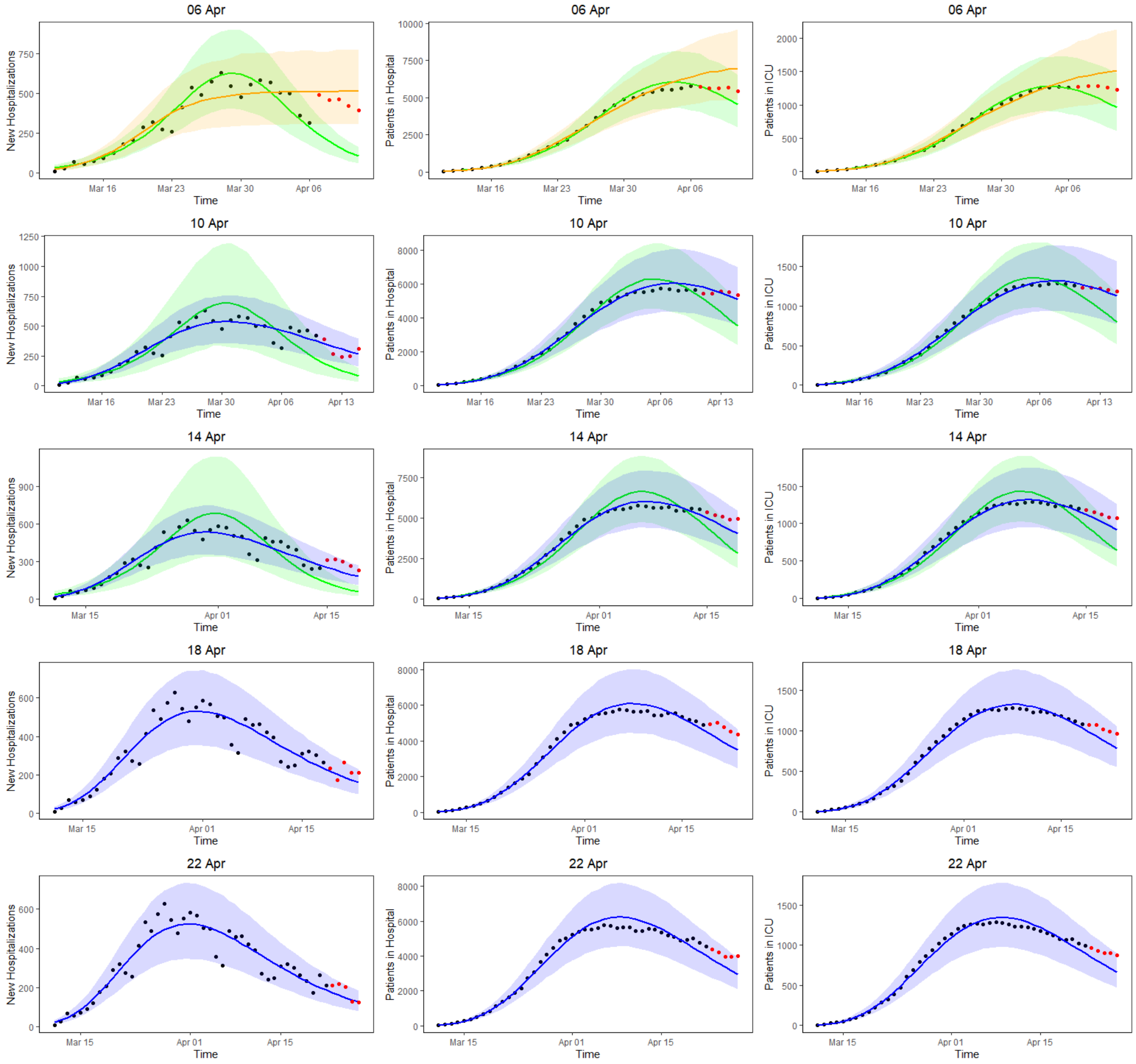

Figure 3: *5-day ahead prediction for number of new covid-19 hospitalization, patients in hospital and patients in ICU from the joint process with gamma distribution. The dots are observed data, where black and red ones correspond to calibration and prediction period, respectively. The line and envelope are posterior mean and 95% CI for models from Phase 1 (purple), Phase 2 (orange), Phase 3 (green) and Phase 4 (blue). Column correspond to new hospitalizations (left), total number of patients in hospital (middle) and number of patients in ICU (right). Rows correspond to different prediction dates during the epidemic.* ***(cont'd)***

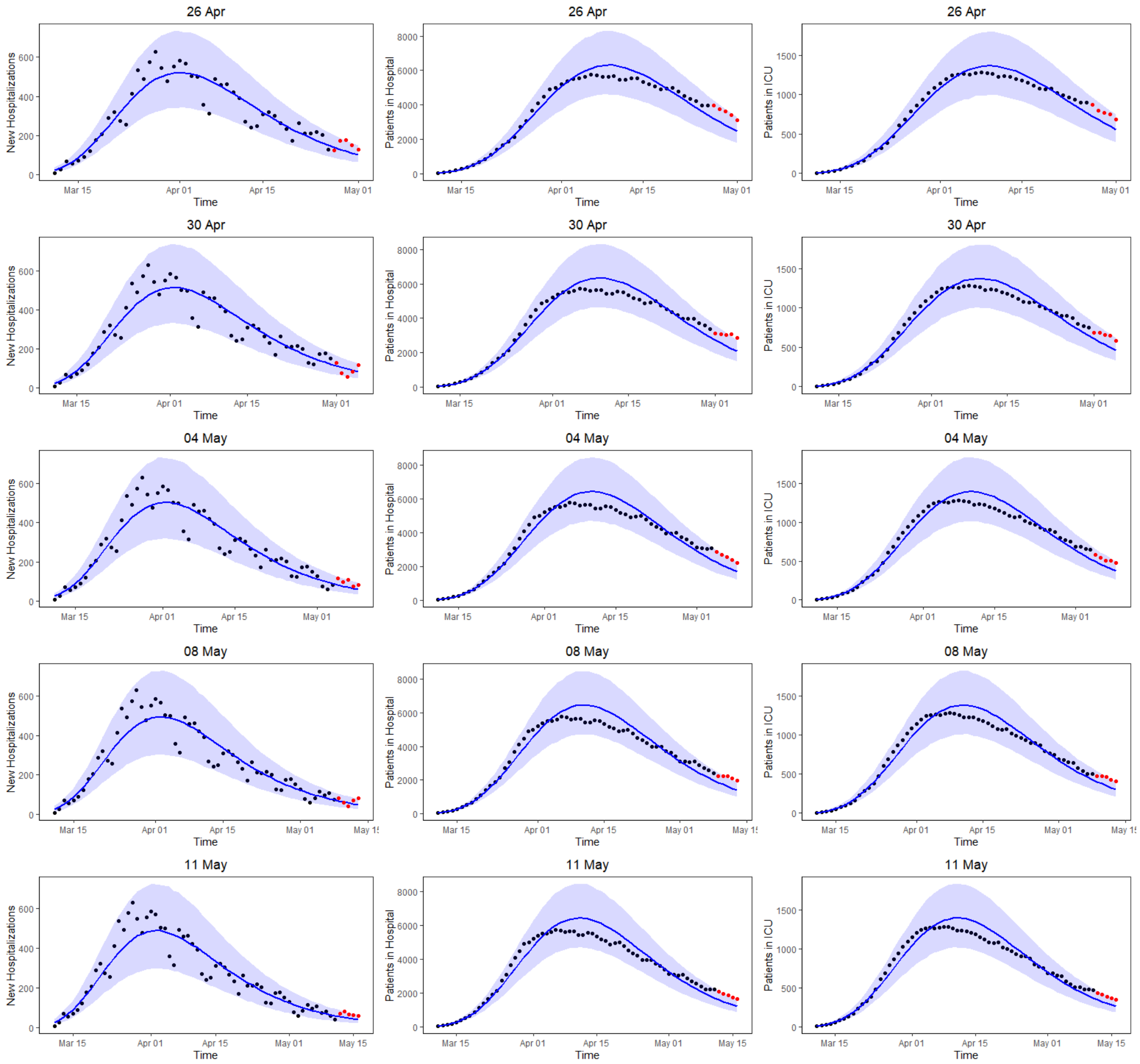

Figure 3: *5-day ahead prediction for number of new covid-19 hospitalization, patients in hospital and patients in ICU from the joint process with gamma distribution. The dots are observed data, where black and red ones correspond to calibration and prediction period, respectively. The line and envelope are posterior mean and 95% CI for models from Phase 1 (purple), Phase 2 (orange), Phase 3 (green) and Phase 4 (blue). Column correspond to new hospitalizations (left), total number of patients in hospital (middle) and number of patients in ICU (right). Rows correspond to different prediction dates during the epidemic.* ***(cont'd)***

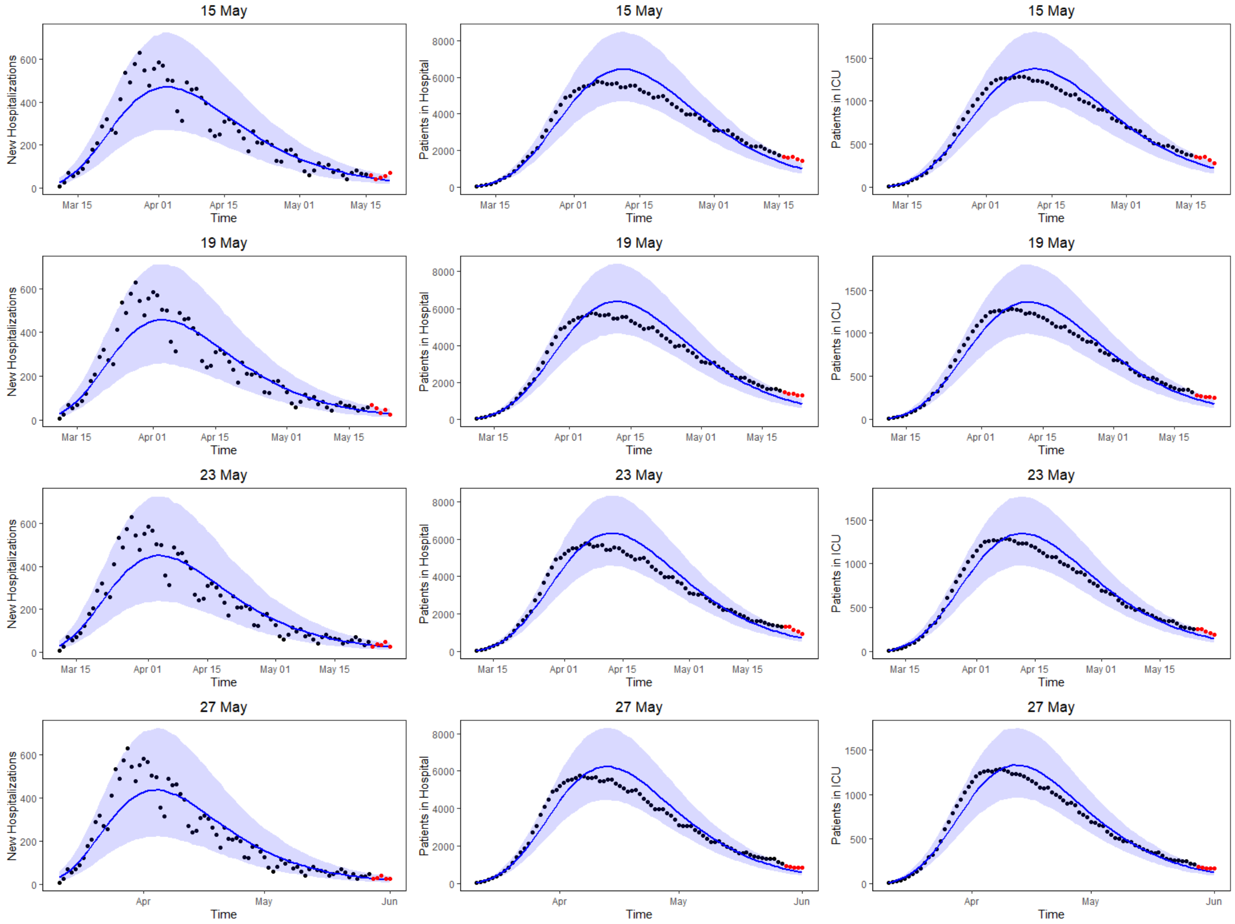

Figure 3: *5-day ahead prediction for number of new covid-19 hospitalization, patients in hospital and patients in ICU from the joint process with gamma distribution. The dots are observed data, where black and red ones correspond to calibration and prediction period, respectively. The line and envelope are posterior mean and 95% CI for models from Phase 1 (purple), Phase 2 (orange), Phase 3 (green) and Phase 4 (blue). Column correspond to new hospitalizations (left), total number of patients in hospital (middle) and number of patients in ICU (right). Rows correspond to different prediction dates during the epidemic.* ***(cont'd)***

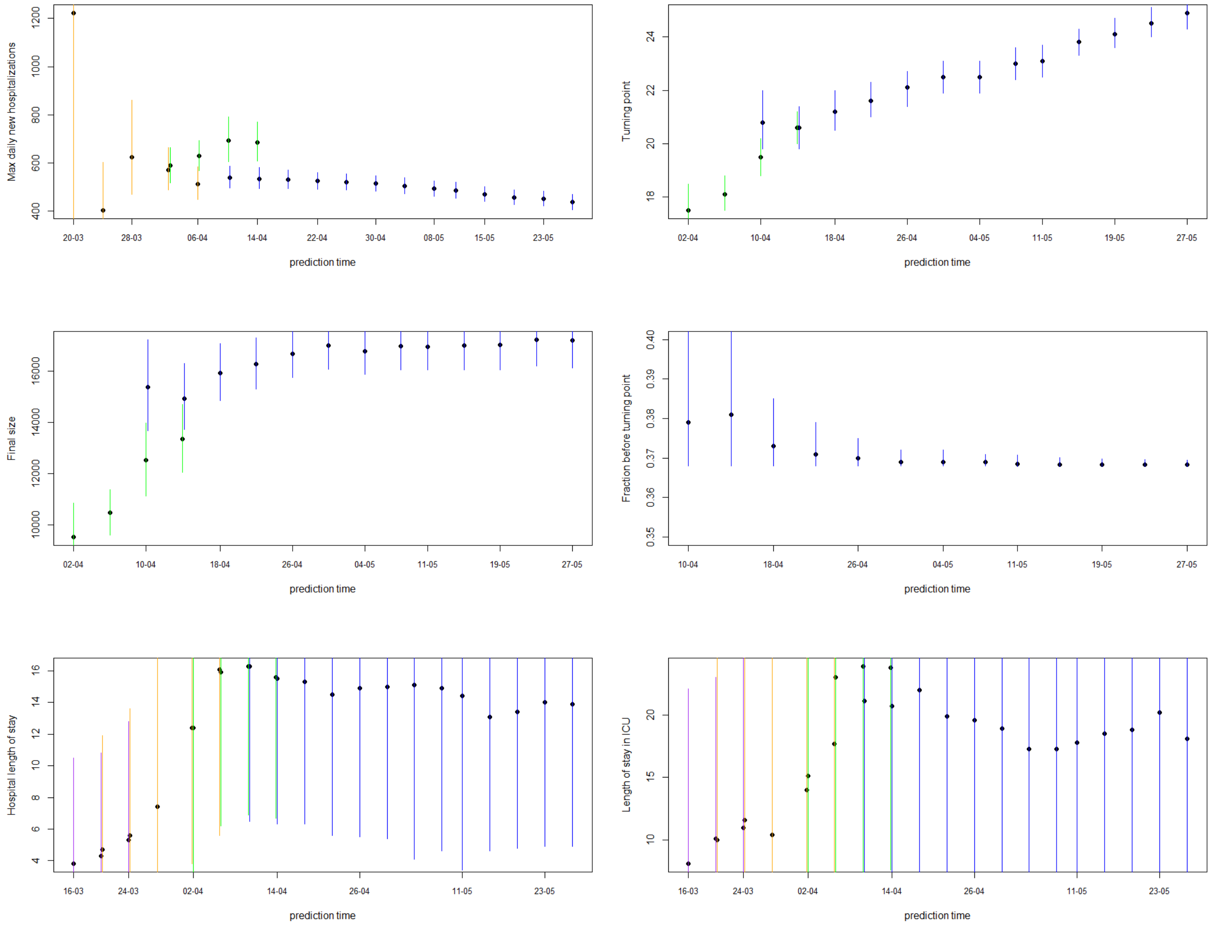

Figure 4: *Model estimates from the joint process with gamma distribution for maximum daily new hospitalizations, turning point, final size, fraction before turning point and length of stay in hospital and ICU. The dots and lines are posterior means and 95% CI for models from Phase 1 (purple), Phase 2 (orange), Phase 3 (green) and Phase 4 (blue), respectively.*

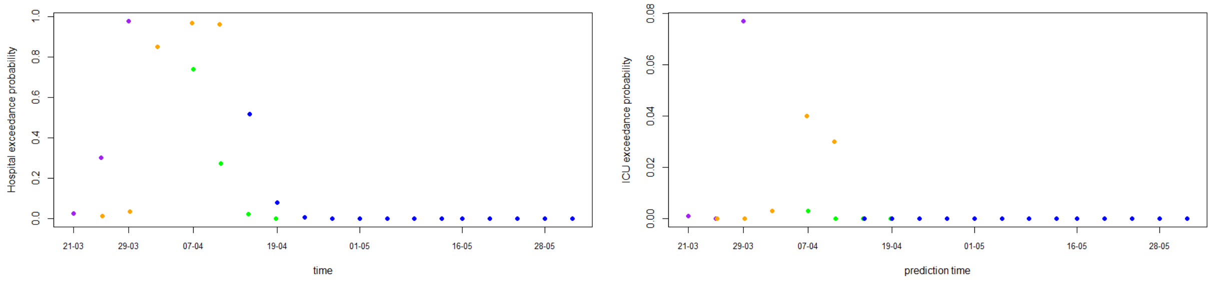

Figure 5: *Model prediction for hospital and ICU exceedance probability on Day 5 ahead from the joint process with gamma distribution. The dots are posterior means for models from Phase 1 (purple), Phase 2 (orange), Phase 3 (green) and Phase 4 (blue), respectively.*

Appendix D: Additional model prediction performance measures

Univariate model

Table 13: *Model goodness of fit and prediction performance via RMSE for covid-19 pandemic in Belgium from March to June 2020*

			RMSE (new hosp)			RMSE (patients in hosp)			RMSE (patients in ICU)		
Date	Phase	*WAIC*	5-day	7-day	10-day	5-day	7-day	10-day	5-day	7-day	10-day
16 Mar	P_1	60.1	619.9	1917.0	11824.5	1100.1	3675.2	23012.1	301.2	944.0	5661.5
20 Mar	P_1	101.5	558.5	1060.5	2730.3	1107.9	2446.6	7215.1	312.7	648.1	1842.1
	P_2	103.4	83.1	93.8	97.1	333.7	538.3	848.1	41.9	74.2	107.6
24 Mar	P_1	152.8	686.4	1198.0	2434.7	1227.1	2792.0	7292.1	395.3	779.8	1853.4
	P_2	145.2	192.5	179.0	176.9	1149.0	1378.2	1566.8	172.2	212.7	262.1
28 Mar	P_2	195.4	46.5	67.8	139.7	724.0	648.0	547.6	30.2	28.4	72.2
02 Apr	P_2	250.4	162.7	150.7	176.9	454.2	387.2	423.6	53.9	83.0	149.6
	P_3	258.3	150.0	207.4	230.2	1091.6	1281.0	1580.9	137.2	193.5	267.5
06 Apr	P_2	304.3	75.9	151.8	175.42	402.0	342.4	371.2	61.0	94.1	154.7
	P_3	301.5	264.6	241.9	243.8	1387.5	1615.7	1921.0	216.4	267.4	332.0
10 Apr	P_3	367.8	113.5	148.3	152.7	980.8	1133.1	1385.2	88.6	123.2	178.8
	P_4	341.2	50.7	58.4	53.4	804.3	832.1	916.8	28.1	32.3	50.0
14 Apr	P_3	413.6	163.7	163.0	165.7	1168.0	1363.6	1490.1	120.0	162.1	203.7
	P_4	383.6	75.6	77.3	81.3	991.8	1095.1	1122.9	80.8	100.7	117.5
18 Apr	P_4	427.92	49.32	57.11	53.21	902.76	872.04	904.14	51.59	55.71	70.35
22 Apr	P_4	470.04	43.46	52.43	49.44	774.12	798.97	775.83	69.59	65.78	64.96
26 Apr	P_4	512.35	45.57	39.62	38.10	738.45	724.55	749.65	25.66	26.38	27.93
30 Apr	P_4	557.06	26.30	29.09	26.28	561.69	559.28	532.64	19.23	17.62	17.96
04 May	P_4	592.71	32.99	28.17	30.06	547.52	558.16	555.66	8.25	22.34	24.82
08 May	P_4	634.66	21.74	23.23	22.25	450.09	442.31	441.15	17.81	15.63	18.01
11 May	P_4	658.7	27.2	24.5	28.6	428.8	440.8	449.0	14.2	16.7	15.0
15 May	P_4	702.40	24.64	23.68	22.18	335.28	347.80	382.65	14.01	12.00	18.06
19 May	P_4	739.45	23.22	21.45	20.93	391.20	410.47	381.67	17.20	17.30	14.79
23 May	P_4	785.38	14.91	15.63	14.81	333.50	301.44	290.45	7.27	8.63	11.87
27 May	P_4	823.1	11.4	11.5	12.4	174.8	193.9	189.7	24.7	21.5	18.7

Table 14: *Model prediction performance via MAPE for the COVID pandemic in Belgium from March to June 2020*

		MAPE (new hosp)			MAPE (patients in hosp)			MAPE (patients in ICU)		
Date	Phase	5-day	7-day	10-day	5-day	7-day	10-day	5-day	7-day	10-day
16 Mar	P_1	1.92	4.97	15.08	0.84	1.63	5.12	1.10	2.09	5.83
20 Mar	P_1	1.35	1.90	3.90	0.41	0.65	1.24	0.58	0.83	1.54
	P_2	0.42	0.45	0.51	0.23	0.27	0.33	0.21	0.25	0.30
24 Mar	P_1	1.11	1.86	3.49	0.30	0.49	1.02	0.41	0.64	1.21
	P_2	0.35	0.34	0.34	0.29	0.32	0.33	0.21	0.23	0.25
28 Mar	P_2	0.22	0.24	0.37	0.16	0.15	0.14	0.09	0.10	0.11
02 Apr	P_2	0.42	0.38	0.47	0.09	0.08	0.09	0.07	0.08	0.12
	P_3	0.33	0.42	0.52	0.19	0.22	0.27	0.12	0.15	0.20
06 Apr	P_2	0.25	0.47	0.56	0.08	0.08	0.08	0.07	0.09	0.12
	P_3	0.60	0.62	0.68	0.24	0.28	0.34	0.17	0.20	0.26
10 Apr	P_3	0.38	0.47	0.54	0.18	0.21	0.25	0.10	0.11	0.15
	P_4	0.23	0.24	0.24	0.14	0.15	0.17	0.05	0.06	0.06
14 Apr	P_3	0.58	0.61	0.67	0.23	0.27	0.31	0.11	0.15	0.19
	P_4	0.27	0.28	0.32	0.19	0.21	0.23	0.07	0.09	0.11
18 Apr	P_4	0.24	0.27	0.27	0.19	0.19	0.21	0.06	0.06	0.08
22 Apr	P_4	0.23	0.28	0.29	0.19	0.20	0.21	0.08	0.08	0.08
26 Apr	P_4	0.29	0.30	0.30	0.20	0.21	0.23	0.05	0.05	0.05
30 Apr	P_4	0.32	0.33	0.31	0.18	0.19	0.19	0.05	0.05	0.05
04 May	P_4	0.33	0.30	0.35	0.21	0.23	0.24	0.04	0.05	0.06
08 May	P_4	0.31	0.34	0.35	0.21	0.21	0.23	0.05	0.05	0.05
11 May	P_4	0.39	0.38	0.43	0.23	0.25	0.27	0.05	0.05	0.05
15 May	P_4	0.38	0.39	0.40	0.21	0.23	0.26	0.05	0.05	0.07
19 May	P_4	0.39	0.41	0.43	0.29	0.31	0.31	0.07	0.07	0.06
23 May	P_4	0.37	0.39	0.41	0.28	0.27	0.28	0.05	0.06	0.07
27 May	P_4	0.36	0.38	0.42	0.20	0.24	0.24	0.13	0.12	0.11

Table 15: *Model prediction performance via the coverage of the 95% prediction interval for the COVID pandemic in Belgium from March to June 2020*

		PI coverage (new hosp)			PI coverage (patients in hosp)			PI coverage (patients in ICU)		
Date	Phase	5-day	7-day	10-day	5-day	7-day	10-day	5-day	7-day	10-day
16 Mar	P_1	1.0	1.0	1.0	1.0	1.0	1.0	1.0	1.0	1.0
20 Mar	P_1	0.4	0.3	0.2	1.0	0.7	0.5	0.4	0.3	0.2
	P_2	1.0	1.0	1.0	1.0	1.0	1.0	1.0	1.0	1.0
24 Mar	P_1	0.8	0.6	0.4	1.0	0.9	0.6	0.8	0.6	0.4
	P_2	1.0	1.0	1.0	0.0	0.0	0.0	1.0	1.0	1.0
28 Mar	P_2	1.0	1.0	0.9	1.0	1.0	1.0	1.0	1.0	1.0
02 Apr	P_2	0.6	0.7	0.7	1.0	1.0	1.0	1.0	1.0	0.8
	P_3	0.8	0.6	0.4	0.6	0.4	0.3	1.0	0.7	0.5
06 Apr	P_2	1.0	0.7	0.7	1.0	1.0	1.0	1.0	1.0	0.8
	P_3	0.0	0.0	0.0	0.0	0.0	0.0	0.2	0.1	0.1
10 Apr	P_3	0.8	0.6	0.4	0.4	0.3	0.2	1.0	0.9	0.6
	P_4	1.0	1.0	1.0	0.2	0.1	0.1	1.0	1.0	1.0
14 Apr	P_3	0.0	0.0	0.0	0.0	0.0	0.0	0.8	0.6	0.4
	P_4	1.0	0.9	0.6	0.0	0.0	0.0	1.0	0.7	0.5
18 Apr	P_4	1.0	0.7	0.7	0.0	0.0	0.0	1.0	1.0	0.8
22 Apr	P_4	1.0	0.7	0.7	0.0	0.0	0.0	0.6	0.7	0.8
26 Apr	P_4	0.8	0.9	0.7	0.0	0.0	0.0	1.0	1.0	1.0
30 Apr	P_4	1.0	0.9	0.9	0.0	0.0	0.0	1.0	1.0	1.0
04 May	P_4	0.6	0.7	0.5	0.0	0.0	0.0	1.0	0.9	0.9
08 May	P_4	0.8	0.6	0.5	0.0	0.0	0.0	1.0	1.0	0.9
11 May	P_4	0.2	0.3	0.2	0.0	0.0	0.0	1.0	1.0	1.0
15 May	P_4	0.6	0.6	0.5	0.0	0.0	0.0	1.0	1.0	0.9
19 May	P_4	0.4	0.3	0.3	0.0	0.0	0.0	1.0	1.0	1.0
23 May	P_4	0.8	0.7	0.7	0.0	0.0	0.0	1.0	1.0	0.9
27 May	P_4	0.8	0.7	0.6	0.0	0.0	0.0	0.4	0.6	0.7

Table 16: *Model prediction performance via the mean interval score for the COVID pandemic in Belgium from March to June 2020*

		MIS (new hosp)			MIS (patients in hosp)			MIS (patients in ICU)		
Date	Phase	5-day	7-day	10-day	5-day	7-day	10-day	5-day	7-day	10-day
16 Mar	P_1	2790.5	7138.7	33809.7	5122.7	13900.1	68560.6	1253.0	3400.1	16758.9
20 Mar	P_1	5734.4	28266.0	146551.5	2873.4	40062.5	344809.8	3399.2	18583.6	110085.5
	P_2	701.6	883.0	1179.2	1839.1	2622.3	4154.7	448.5	639.6	1014.3
24 Mar	P_1	4210.8	27595.0	119522.3	4595.1	33330.5	379513.4	1499.4	18000.8	115237.1
	P_2	426.8	442.6	457.5	62505.3	111191.8	159581.6	336.4	383.1	452.3
28 Mar	P_2	619.0	637.0	1093.5	2032.6	2270.3	2608.9	494.9	553.3	636.7
02 Apr	P_2	3181.2	3181.1	7262.3	1655.3	1716.0	1797.8	402.9	418.4	3116.5
	P_3	4802.0	18162.3	39428.6	28482.8	78414.6	211635.3	447.5	3148.1	19706.6
06 Apr	P_2	467.0	4307.6	6545.8	1688.4	1720.7	1758.3	412.6	420.7	4346.5
	P_3	32898.0	42313.3	67485.2	143560.2	264941.7	505146.3	12117.1	30279.9	69345.7
10 Apr	P_3	3090.4	12940.4	24784.0	35133.5	87347.7	222974.3	439.2	494.1	10845.4
	P_4	260.2	248.9	231.3	32486.6	51552.0	101754.0	309.6	311.4	313.6
14 Apr	P_3	14436.6	22416.4	38633.5	89684.7	185463.0	337356.9	1126.1	8580.7	26903.7
	P_4	180.0	1569.6	4354.5	99918.7	166935.7	253555.9	234.1	1676.2	5720.9
18 Apr	P_4	149.2	780.1	1647.7	92118.1	122629.8	194026.1	209.3	203.6	1086.4
22 Apr	P_4	117.6	1431.0	1940.6	82146.6	125452.7	176622.9	773.9	768.6	760.8
26 Apr	P_4	497.8	491.1	1042.2	86363.4	120133.2	187518.8	149.8	144.4	137.3
30 Apr	P_4	82.8	357.7	351.0	57224.5	83151.6	114369.6	128.7	123.7	116.2
04 May	P_4	541.0	537.1	1852.2	68453.6	101422.0	149248.4	98.8	209.2	203.1
08 May	P_4	493.6	730.3	964.9	52908.7	74214.6	109767.6	90.4	86.5	158.6
11 May	P_4	803.6	841.1	3157.7	55655.4	83135.6	126180.0	76.4	72.9	68.0
15 May	P_4	1119.4	1557.0	2074.0	39448.7	60648.1	103029.9	66.1	62.9	585.9
19 May	P_4	1113.8	1191.9	1869.2	56065.9	84831.0	111247.8	55.9	53.4	49.9
23 May	P_4	431.4	749.7	947.4	43755.9	52515.9	74382.0	52.2	49.9	352.3
27 May	P_4	227.4	306.0	664.0	16696.5	29302.8	42485.7	914.9	912.8	909.9

Joint model

Table 17: *Model goodness of fit and prediction performance via RMSE for covid-19 pandemic in Belgium from March to June 2020 from the joint process*

			RMSE (new hosp)			RMSE (patients in hosp)			RMSE (patients in ICU)		
Date	Phase	$WAIC$	5-day	7-day	10-day	5-day	7-day	10-day	5-day	7-day	10-day
16 Mar	P_1	153.57	508.87	1224.45	3841.25	630.50	1789.65	6889.94	179.00	468.55	1676.15
20 Mar	P_1	273.50	473.15	858.43	2068.49	964.96	2011.93	5562.35	216.86	447.78	1256.78
	P_2	272.60	80.38	88.67	92.85	307.03	559.10	975.92	67.34	119.11	186.24
24 Mar	P_1	423.30	689.31	1182.69	2318.99	1754.39	3440.71	8114.75	388.17	749.30	1736.24
	P_2	407.81	185.70	171.39	167.99	772.15	990.34	1192.49	161.00	199.31	253.37
28 Mar	P_2	561.09	41.60	62.11	136.55	405.98	635.82	945.93	72.49	112.77	194.35
02 Apr	P_2	755.53	144.29	132.06	157.84	1278.22	1534.13	1868.94	296.95	362.80	463.43
	P_3	767.70	177.76	229.51	248.05	380.25	355.72	608.77	149.33	141.34	123.22
06 Apr	P_2	924.65	58.10	134.88	157.59	1304.15	1439.216	1634.64	404.45	462.10	544.13
	P_3	924.90	280.92	256.36	255.36	863.77	1282.17	1844.07	49.11	99.29	179.61
10 Apr	P_3	1115.37	163.00	190.47	189.32	1683.69	2027.10	2483.87	200.97	262.98	354.72
	P_4	1067.19	50.56	58.96	54.01	475.95	605.34	861.92	56.27	48.32	65.10
14 Apr	P_3	1293.40	194.89	190.59	188.97	1713.96	2065.20	2352.66	278.71	344.93	417.61
	P_4	1220.31	71.98	73.85	77.60	774.46	998.26	1155.26	78.41	118.12	160.00
18 Apr	P_4	1374.41	45.37	52.21	48.57	903.60	939.98	1074.59	122.33	141.16	174.04
22 Apr	P_4	1529.63	38.71	47.10	44.23	784.10	866.01	906.47	148.22	153.63	164.70
26 Apr	P_4	1683.22	40.56	36.43	34.54	767.92	800.19	885.98	124.83	134.27	142.31
30 Apr	P_4	1840.58	26.74	27.60	24.29	687.12	713.57	718.67	109.72	106.58	113.35
04 May	P_4	1993.09	26.87	23.01	25.00	602.62	639.70	664.95	81.53	97.22	104.15
08 May	P_4	2141.13	18.23	19.12	18.01	543.92	548.19	559.94	95.21	95.28	101.29
11 May	P_4	2251.76	21.14	18.79	23.55	477.99	500.85	519.91	81.26	89.96	90.00
15 May	P_4	2399.95	19.61	18.82	17.54	425.07	441.48	478.21	77.48	75.45	80.16
19 May	P_4	2546.30	18.21	16.46	16.18	410.81	433.29	406.57	58.83	64.24	61.75
23 May	P_4	2691.42	10.81	11.37	10.56	357.73	325.97	315.61	55.79	51.26	52.61
27 May	P_4	2837.38	6.88	7.00	8.01	182.07	201.58	197.36	31.98	37.32	35.11

Table 18: *Model prediction performance via MAPE for the COVID pandemic in Belgium from March to June 2020 from the joint process*

		MAPE (new hosp)			MAPE (patients in hosp)			MAPE (patients in ICU)		
Date	Phase	5-day	7-day	10-day	5-day	7-day	10-day	5-day	7-day	10-day
16 Mar	P_1	1.76	3.53	6.41	0.50	0.85	1.86	0.70	1.14	2.17
20 Mar	P_1	1.17	1.58	3.05	0.38	0.56	1.00	0.42	0.59	1.07
	P_2	0.37	0.40	0.44	0.22	0.26	0.32	0.22	0.26	0.32
24 Mar	P_1	1.10	1.84	3.36	0.39	0.61	1.17	0.40	0.62	1.15
	P_2	0.34	0.33	0.32	0.21	0.24	0.26	0.22	0.23	0.26
28 Mar	P_2	0.21	0.22	0.35	0.16	0.18	0.21	0.16	0.17	0.20
02 Apr	P_2	0.38	0.34	0.42	0.25	0.29	0.34	0.25	0.29	0.36
	P_3	0.37	0.46	0.56	0.17	0.19	0.21	0.18	0.18	0.18
06 Apr	P_2	0.23	0.43	0.51	0.26	0.28	0.31	0.33	0.37	0.44
	P_3	0.63	0.66	0.71	0.22	0.27	0.35	0.18	0.19	0.23
10 Apr	P_3	0.53	0.60	0.67	0.30	0.37	0.46	0.20	0.24	0.31
	P_4	0.23	0.24	0.24	0.15	0.16	0.19	0.15	0.15	0.16
14 Apr	P_3	0.68	0.71	0.76	0.33	0.40	0.48	0.26	0.31	0.39
	P_4	0.26	0.27	0.31	0.18	0.21	0.25	0.14	0.16	0.19
18 Apr	P_4	0.23	0.25	0.25	0.20	0.22	0.26	0.16	0.17	0.20
22 Apr	P_4	0.22	0.26	0.27	0.20	0.23	0.25	0.18	0.19	0.21
26 Apr	P_4	0.27	0.30	0.29	0.22	0.24	0.28	0.18	0.20	0.22
30 Apr	P_4	0.34	0.33	0.30	0.23	0.25	0.27	0.19	0.19	0.21
04 May	P_4	0.28	0.27	0.31	0.24	0.26	0.29	0.18	0.20	0.23
08 May	P_4	0.30	0.31	0.30	0.26	0.27	0.30	0.22	0.23	0.26
11 May	P_4	0.32	0.30	0.36	0.26	0.28	0.31	0.22	0.25	0.27
15 May	P_4	0.32	0.32	0.33	0.27	0.29	0.33	0.24	0.25	0.28
19 May	P_4	0.34	0.33	0.34	0.30	0.33	0.33	0.23	0.26	0.27
23 May	P_4	0.33	0.34	0.34	0.30	0.29	0.31	0.25	0.24	0.27
27 May	P_4	0.31	0.31	0.33	0.22	0.25	0.26	0.20	0.23	0.23

Table 19: *Model prediction performance via the coverage of the 95% prediction interval for the COVID pandemic in Belgium from March to June 2020 from the joint process*

		PI coverage (new hosp)			PI coverage (patients in hosp)			PI coverage (patients in ICU)		
Date	Phase	5-day	7-day	10-day	5-day	7-day	10-day	5-day	7-day	10-day
16 Mar	P_1	1.0	0.7	0.5	1.0	0.9	0.6	1.0	0.7	0.5
20 Mar	P_1	0.2	0.1	0.1	0.8	0.6	0.4	1.0	0.7	0.5
	P_2	1.0	1.0	1.0	1.0	1.0	1.0	1.0	1.0	1.0
24 Mar	P_1	0.8	0.6	0.4	0.8	0.6	0.4	0.6	0.4	0.3
	P_2	1.0	1.0	1.0	1.0	1.0	1.0	1.0	1.0	1.0
28 Mar	P_2	1.0	1.0	0.9	1.0	1.0	1.0	1.0	1.0	1.0
02 Apr	P_2	0.8	0.9	0.8	1.0	1.0	1.0	1.0	1.0	0.8
	P_3	0.6	0.4	0.3	1.0	1.0	1.0	1.0	1.0	1.0
06 Apr	P_2	1.0	0.7	0.7	1.0	1.0	1.0	1.0	1.0	0.7
	P_3	0.0	0.0	0.0	1.0	1.0	1.0	1.0	1.0	1.0
10 Apr	P_3	0.2	0.1	0.1	0.6	0.4	0.3	1.0	1.0	0.7
	P_4	1.0	1.0	1.0	1.0	1.0	1.0	1.0	1.0	1.0
14 Apr	P_3	0.0	0.0	0.0	0.4	0.3	0.2	0.8	0.6	0.4
	P_4	1.0	0.9	0.7	1.0	0.7	0.5	1.0	1.0	1.0
18 Apr	P_4	1.0	0.9	0.8	1.0	1.0	0.7	1.0	1.0	0.9
22 Apr	P_4	1.0	0.7	0.7	0.8	0.6	0.4	1.0	1.0	0.9
26 Apr	P_4	0.8	0.9	0.8	1.0	0.7	0.5	1.0	1.0	0.8
30 Apr	P_4	1.0	1.0	1.0	0.6	0.4	0.3	1.0	1.0	0.9
04 May	P_4	0.8	0.9	0.7	1.0	0.7	0.5	1.0	0.9	0.6
08 May	P_4	0.8	0.9	0.9	0.4	0.3	0.2	1.0	0.9	0.6
11 May	P_4	1.0	1.0	0.7	0.0	0.0	0.0	1.0	0.7	0.5
15 May	P_4	0.8	0.7	0.8	0.4	0.3	0.2	0.6	0.6	0.4
19 May	P_4	0.8	0.9	0.8	0.0	0.0	0.0	0.8	0.6	0.4
23 May	P_4	1.0	1.0	1.0	0.0	0.0	0.0	0.8	0.9	0.7
27 May	P_4	1.0	1.0	1.0	0.8	0.6	0.5	1.0	0.9	0.9

Table 20: *Model prediction performance via the mean interval score for the COVID pandemic in Belgium from March to June 2020 from the joint process*

		MIS (new hosp)			MIS (patients in hosp)			MIS (patients in ICU)		
Date	Phase	5-day	7-day	10-day	5-day	7-day	10-day	5-day	7-day	10-day
16 Mar	P_1	1854.7	14439.2	73476.2	2021.0	5227.5	108358.2	504.2	4470.6	34140.1
20 Mar	P_1	11866.2	44287.6	194558.5	2458.1	59539.8	445769.2	522.2	12743.9	103624.7
	P_2	593.6	745.0	1007.8	1818.9	2505.5	3912.7	414.6	562.6	860.9
24 Mar	P_1	9753.2	52128.1	202085.6	23882.0	175437.9	926290.5	5823.6	41241.9	202470.9
	P_2	414.4	425.9	443.7	2234.4	2507.6	2903.8	490.0	552.4	634.8
28 Mar	P_2	549.0	561.4	1734.8	3721.7	4105.0	4676.7	812.0	891.3	1006.3
02 Apr	P_2	2038.4	2039.4	5442.9	4838.0	5161.9	5570.9	1036.4	1097.3	3771.8
	P_3	7767.4	26091.4	52565.5	4644.3	4904.8	5156.1	956.2	986.0	1020.6
06 Apr	P_2	454.6	3053.9	4414.8	4955.9	5118.3	5351.8	1132.8	1181.9	7446.5
	P_3	39074.6	49972.7	76546.7	4637.3	4781.8	4880.3	1057.8	1091.9	1126.8
10 Apr	P_3	11657.4	27152.4	45243.7	42164.7	136943.3	380327.1	806.2	783.7	14822.6
	P_4	257.8	246.3	229.8	3459.0	3384.9	3267.1	894.2	890.6	875.2
14 Apr	P_3	25200.6	36064.3	55365.4	71934.5	197631.3	438546.9	3267.4	18550.3	58047.0
	P_4	182.2	1451.9	3397.7	2870.4	6869.7	32787.3	704.2	679.7	644.3
18 Apr	P_4	153.4	184.0	731.4	2405.0	2279.3	50943.9	580.0	556.0	1237.8
22 Apr	P_4	122.6	915.0	1103.6	4702.6	15745.9	43499.4	485.6	460.6	1424.6
26 Apr	P_4	261.4	255.0	446.6	1705.8	13241.6	66021.8	407.2	384.1	1232.7
30 Apr	P_4	87.8	82.6	75.5	12080.6	24991.4	50232.1	339.2	319.6	1412.8
04 May	P_4	106.6	102.6	617.3	1176.2	19222.9	55202.9	276.4	1100.1	3357.6
08 May	P_4	99.8	96.3	91.5	13850.0	25310.9	53349.8	222.6	409.6	4231.8
11 May	P_4	50.0	47.3	1523.3	6354.6	21782.4	53790.0	192.2	2300.6	4645.3
15 May	P_4	567.0	644.1	640.7	14582.2	29098.1	65358.4	798.6	1029.3	4776.9
19 May	P_4	241.4	239.3	516.3	19819.4	38545.6	53338.3	573.2	1966.0	2595.8
23 May	P_4	39.8	37.7	35.0	17573.6	18904.9	31945.3	312.0	305.9	1857.7
27 May	P_4	36.4	34.6	31.9	1467.2	6202.0	9449.6	94.8	649.9	643.1

Sensitivity analysis: gamma distribution for length of stay

Table 21: *Model prediction performance via MAPE for the COVID pandemic in Belgium from March to June 2020 from the joint process with gamma distribution*

		MAPE (new hosp)			MAPE (patients in hosp)			MAPE (patients in ICU)		
Date	Phase	5-day	7-day	10-day	5-day	7-day	10-day	5-day	7-day	10-day
16 Mar	P_1	1.27	2.61	4.73	0.46	0.78	1.65	0.57	0.93	1.75
20 Mar	P_1	1.18	1.60	3.10	0.42	0.61	1.08	0.39	0.55	1.02
	P_2	0.38	0.42	0.48	0.21	0.25	0.32	0.26	0.31	0.38
24 Mar	P_1	1.13	1.90	3.47	0.43	0.66	1.26	0.38	0.61	1.14
	P_2	0.33	0.32	0.31	0.19	0.20	0.22	0.28	0.30	0.33
28 Mar	P_2	0.21	0.24	0.37	0.17	0.20	0.24	0.15	0.16	0.19
02 Apr	P_2	0.40	0.37	0.45	0.24	0.27	0.32	0.19	0.22	0.26
	P_3	0.36	0.46	0.55	0.18	0.18	0.20	0.17	0.19	0.23
06 Apr	P_2	0.25	0.46	0.55	0.24	0.25	0.28	0.20	0.22	0.26
	P_3	0.63	0.65	0.71	0.16	0.20	0.27	0.19	0.23	0.30
10 Apr	P_3	0.54	0.62	0.68	0.23	0.29	0.38	0.23	0.28	0.36
	P_4	0.23	0.24	0.23	0.14	0.14	0.16	0.14	0.14	0.16
14 Apr	P_3	0.68	0.71	0.76	0.31	0.37	0.45	0.29	0.35	0.43
	P_4	0.26	0.27	0.31	0.16	0.19	0.22	0.15	0.17	0.20
18 Apr	P_4	0.23	0.26	0.25	0.19	0.20	0.24	0.17	0.19	0.22
22 Apr	P_4	0.22	0.26	0.27	0.19	0.22	0.24	0.19	0.21	0.23
26 Apr	P_4	0.26	0.30	0.29	0.21	0.23	0.27	0.19	0.21	0.23
30 Apr	P_4	0.34	0.33	0.30	0.22	0.24	0.26	0.20	0.20	0.22
04 May	P_4	0.27	0.26	0.30	0.24	0.26	0.29	0.19	0.22	0.24
08 May	P_4	0.30	0.31	0.30	0.25	0.27	0.30	0.23	0.24	0.27
11 May	P_4	0.31	0.30	0.36	0.25	0.28	0.31	0.23	0.26	0.28
15 May	P_4	0.32	0.32	0.33	0.27	0.29	0.33	0.24	0.25	0.28
19 May	P_4	0.34	0.33	0.34	0.30	0.33	0.33	0.23	0.26	0.27
23 May	P_4	0.33	0.34	0.34	0.30	0.30	0.31	0.26	0.25	0.28
27 May	P_4	0.31	0.31	0.34	0.23	0.25	0.27	0.21	0.24	0.24

Table 22: *Model prediction performance via SMAPE for the COVID pandemic in Belgium from March to June 2020 from the joint process with gamma distribution*

		SMAPE (new hosp)			SMAPE (patients in hosp)			SMAPE (patients in ICU)		
Date	Phase	5-day	7-day	10-day	5-day	7-day	10-day	5-day	7-day	10-day
16 Mar	P_1	0.65	0.84	1.03	0.34	0.46	0.66	0.39	0.52	0.70
20 Mar	P_1	0.67	0.79	0.98	0.32	0.42	0.59	0.30	0.39	0.57
	P_2	0.38	0.43	0.48	0.23	0.28	0.37	0.30	0.38	0.47
24 Mar	P_1	0.63	0.80	1.01	0.33	0.45	0.64	0.30	0.41	0.60
	P_2	0.42	0.39	0.39	0.21	0.24	0.26	0.34	0.37	0.42
28 Mar	P_2	0.20	0.21	0.29	0.16	0.18	0.21	0.15	0.16	0.17
02 Apr	P_2	0.31	0.29	0.33	0.21	0.23	0.26	0.17	0.19	0.22
	P_3	0.47	0.65	0.84	0.16	0.17	0.21	0.17	0.20	0.26
06 Apr	P_2	0.22	0.34	0.39	0.20	0.21	0.23	0.18	0.19	0.22
	P_3	0.94	0.99	1.14	0.17	0.22	0.34	0.22	0.27	0.37
10 Apr	P_3	0.79	0.95	1.09	0.28	0.36	0.50	0.28	0.35	0.47
	P_4	0.22	0.24	0.24	0.14	0.14	0.17	0.14	0.15	0.17
14 Apr	P_3	1.06	1.13	1.25	0.38	0.48	0.62	0.36	0.45	0.59
	P_4	0.31	0.32	0.38	0.17	0.21	0.26	0.16	0.19	0.23
18 Apr	P_4	0.26	0.30	0.30	0.21	0.23	0.28	0.19	0.21	0.26
22 Apr	P_4	0.25	0.31	0.31	0.22	0.25	0.29	0.22	0.24	0.27
26 Apr	P_4	0.31	0.30	0.32	0.25	0.27	0.32	0.22	0.24	0.27
30 Apr	P_4	0.31	0.32	0.30	0.26	0.29	0.31	0.22	0.23	0.26
04 May	P_4	0.33	0.30	0.36	0.28	0.31	0.35	0.22	0.25	0.29
08 May	P_4	0.32	0.34	0.35	0.30	0.32	0.36	0.26	0.28	0.32
11 May	P_4	0.39	0.37	0.46	0.30	0.33	0.38	0.27	0.31	0.33
15 May	P_4	0.39	0.40	0.41	0.33	0.36	0.41	0.29	0.30	0.34
19 May	P_4	0.41	0.41	0.43	0.37	0.40	0.41	0.27	0.31	0.32
23 May	P_4	0.38	0.41	0.42	0.37	0.36	0.38	0.30	0.30	0.33
27 May	P_4	0.36	0.38	0.43	0.26	0.30	0.32	0.24	0.28	0.29

Table 23: *Model prediction performance via the coverage of the 95% prediction interval for the COVID pandemic in Belgium from March to June 2020 from the joint process with gamma distribution*

		PI coverage (new hosp)			PI coverage (patients in hosp)			PI coverage (patients in ICU)		
Date	Phase	5-day	7-day	10-day	5-day	7-day	10-day	5-day	7-day	10-day
16 Mar	P_1	1.0	0.7	0.5	1.0	0.9	0.6	1.0	0.9	0.6
20 Mar	P_1	0.2	0.1	0.1	0.6	0.4	0.3	1.0	0.7	0.5
	P_2	1.0	1.0	1.0	1.0	1.0	1.0	1.0	1.0	1.0
24 Mar	P_1	0.8	0.6	0.4	0.6	0.4	0.3	0.8	0.6	0.4
	P_2	1.0	1.0	1.0	1.0	1.0	1.0	1.0	1.0	0.8
28 Mar	P_2	1.0	1.0	0.9	1.0	1.0	1.0	1.0	1.0	1.0
02 Apr	P_2	0.6	0.7	0.7	1.0	1.0	1.0	1.0	1.0	1.0
	P_3	0.8	0.6	0.4	1.0	1.0	1.0	1.0	1.0	1.0
06 Apr	P_2	1.0	0.7	0.7	1.0	1.0	1.0	1.0	1.0	1.0
	P_3	0.0	0.0	0.0	1.0	1.0	0.7	1.0	1.0	0.8
10 Apr	P_3	0.2	0.1	0.1	0.6	0.4	0.3	0.8	0.6	0.4
	P_4	1.0	1.0	1.0	1.0	1.0	1.0	1.0	1.0	1.0
14 Apr	P_3	0.0	0.0	0.0	0.4	0.3	0.2	0.4	0.3	0.2
	P_4	1.0	0.9	0.7	1.0	1.0	0.8	1.0	1.0	1.0
18 Apr	P_4	1.0	0.7	0.7	1.0	1.0	0.7	1.0	1.0	0.7
22 Apr	P_4	1.0	0.7	0.7	0.8	0.6	0.4	1.0	1.0	0.7
26 Apr	P_4	0.8	0.9	0.8	1.0	0.7	0.5	1.0	0.9	0.6
30 Apr	P_4	1.0	1.0	1.0	0.6	0.4	0.3	1.0	1.0	0.8
04 May	P_4	1.0	1.0	0.8	1.0	0.7	0.5	1.0	0.7	0.5
08 May	P_4	0.8	0.9	0.9	0.4	0.3	0.2	1.0	0.9	0.6
11 May	P_4	1.0	1.0	0.7	0.4	0.3	0.2	1.0	0.7	0.5
15 May	P_4	0.8	0.7	0.8	0.4	0.3	0.2	0.6	0.6	0.4
19 May	P_4	0.8	0.9	0.8	0.2	0.1	0.1	0.8	0.6	0.4
23 May	P_4	0.8	0.7	0.8	0.0	0.0	0.0	0.8	0.9	0.6
27 May	P_4	1.0	1.0	1.0	0.8	0.6	0.4	1.0	0.9	0.9

Table 24: *Model prediction performance via the mean interval score for the COVID pandemic in Belgium from March to June 2020 from the joint process with gamma distribution*

		MIS (new hosp)			MIS (patients in hosp)			MIS (patients in ICU)		
Date	Phase	5-day	7-day	10-day	5-day	7-day	10-day	5-day	7-day	10-day
16 Mar	P_1	1171.3	15394.7	76811.6	1811.5	8460.8	139218.5	407.6	2535.6	28919.8
20 Mar	P_1	14006.6	48983.3	210867.9	8831.8	80710.4	538169.0	500.0	11747.6	100433.2
	P_2	640.4	840.7	1223.1	1968.7	2784.1	4610.8	445.0	614.0	990.4
24 Mar	P_1	10693.9	55293.2	211894.6	32300.7	202462.4	1026278.0	6210.4	40636.0	201827.0
	P_2	411.0	421.6	433.4	2322.6	2602.6	3012.8	480.4	536.9	2687.8
28 Mar	P_2	569.6	584.6	2038.1	3918.3	4340.7	4973.4	828.4	915.3	1036.9
02 Apr	P_2	2763.6	2766.7	6607.4	4894.1	5168.5	5556.8	1038.0	1100.7	1185.1
	P_3	7489.0	25573.7	51928.9	4649.7	4827.6	5017.0	1001.4	1047.7	1096.1
06 Apr	P_2	466.2	4226.7	6426.5	4479.0	4570.4	4694.5	1040.0	1071.9	1111.5
	P_3	39152.8	50091.3	76705.7	3721.1	3597.5	84563.8	869.4	862.7	7555.9
10 Apr	P_3	12094.2	27710.1	46041.6	17925.9	88968.4	309492.6	1112.4	10716.6	46616.3
	P_4	256.8	244.4	226.4	3605.6	3532.5	3399.5	820.2	805.7	777.7
14 Apr	P_3	25477.4	36461.3	55882.9	68384.1	198088.8	447504.8	10208.8	33046.2	82821.8
	P_4	181.2	1370.9	3276.4	2874.0	2753.3	13942.2	676.4	650.1	612.2
18 Apr	P_4	151.4	342.0	1089.2	2434.4	2305.6	39047.0	564.8	537.2	4097.0
22 Apr	P_4	121.4	1033.6	1223.5	3771.0	12257.2	35656.9	478.2	452.1	3253.0
26 Apr	P_4	220.8	214.4	366.3	1712.6	9892.7	59630.6	397.2	534.7	2983.7
30 Apr	P_4	86.2	81.1	74.2	11629.4	22706.3	45909.8	333.2	314.3	1887.3
04 May	P_4	67.6	63.6	578.2	1182.2	17987.6	52726.6	270.4	2053.4	5311.8
08 May	P_4	99.4	95.9	91.3	13291.6	24912.2	53069.3	222.2	608.9	4870.6
11 May	P_4	50.6	47.9	1442.9	4646.6	19354.1	49722.0	190.4	2499.6	5363.9
15 May	P_4	566.6	604.1	600.7	15216.8	29854.2	66316.5	917.6	1189.0	4937.0
19 May	P_4	281.0	279.0	596.0	19700.8	37947.0	52659.5	572.8	2205.0	2995.0
23 May	P_4	117.4	155.4	152.8	18452.2	20024.1	33465.0	470.8	464.9	2216.6
27 May	P_4	33.8	32.1	29.8	1669.4	6964.1	10890.4	93.8	768.9	762.3
