# Supplementary figures and images for: Multivariate phenomenological models for real-time short-term forecasts of hospital capacity for COVID-19 in Belgium from March to June 2020
Source: Epidemiol Infect. 2021 Dec 17;150:e12. doi: 10.1017/S0950268821002491 (PMC8755551; doi:10.1017/S0950268821002491)

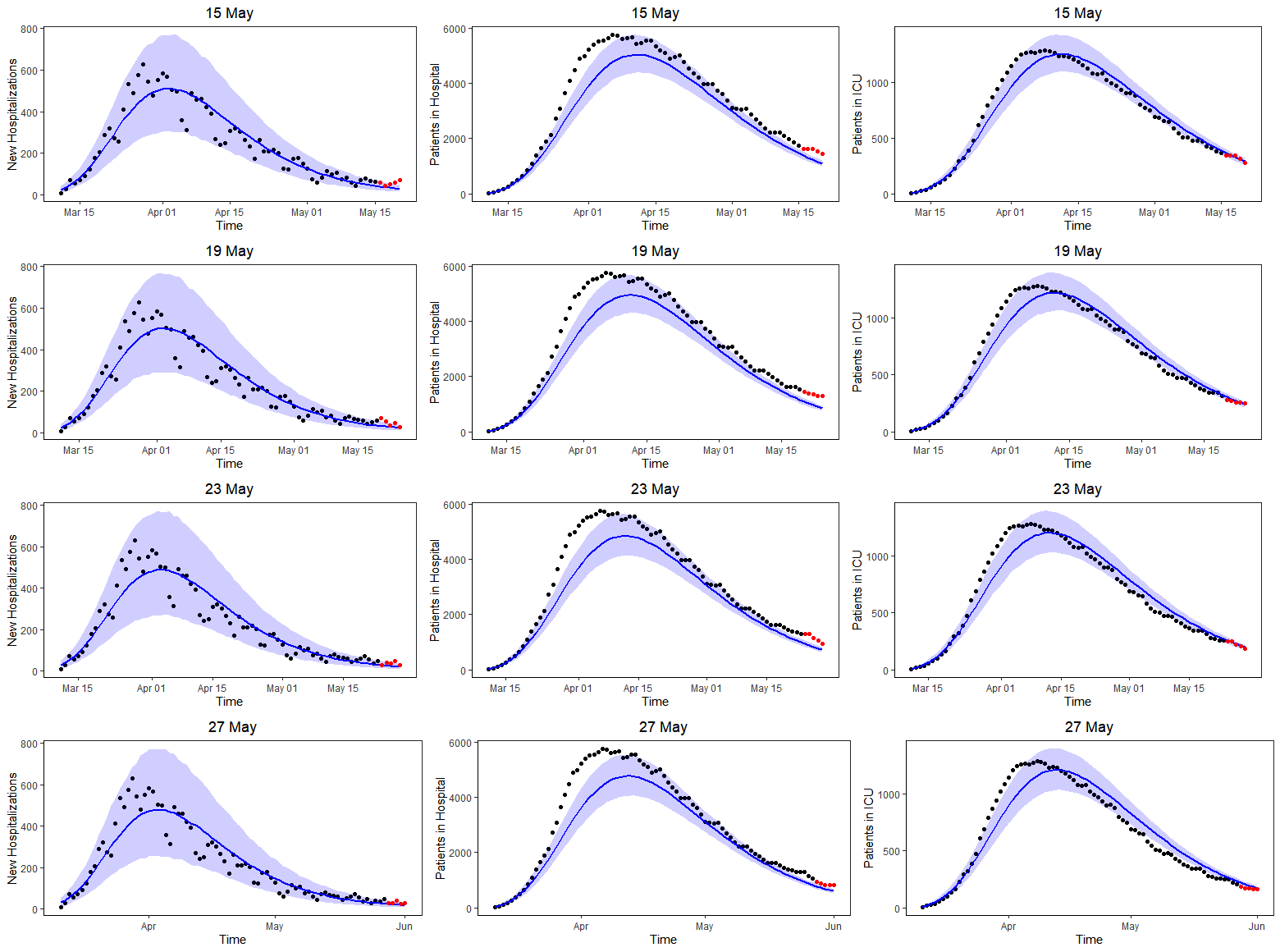

Supplement: Supplementary file 1 [file S0950268821002491sup001.zip › Final Version/figure/appx_Fig1_04.tiff]

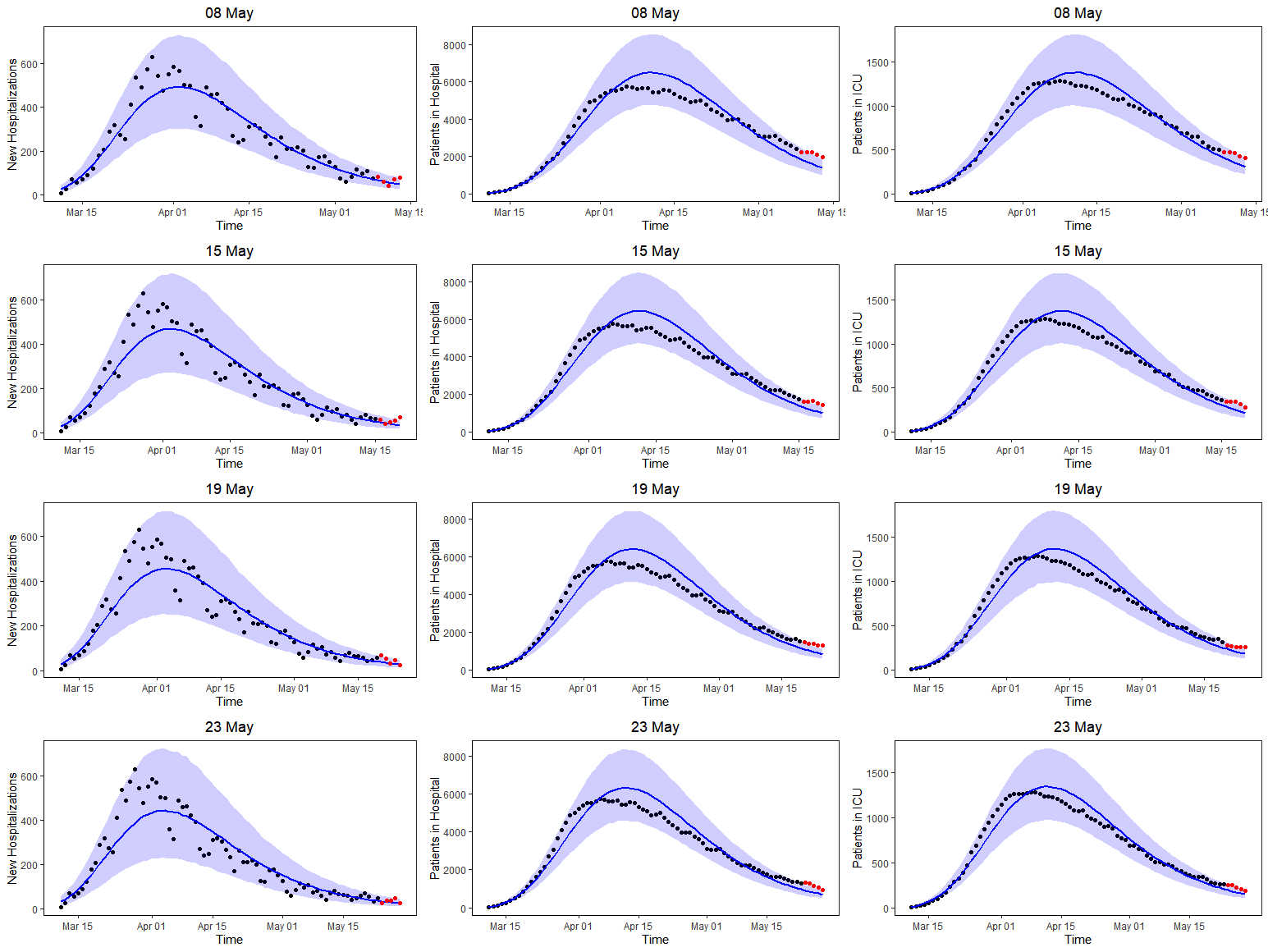

Supplement: Supplementary file 1 [file S0950268821002491sup001.zip › Final Version/figure/appx_Fig2_02.tiff]

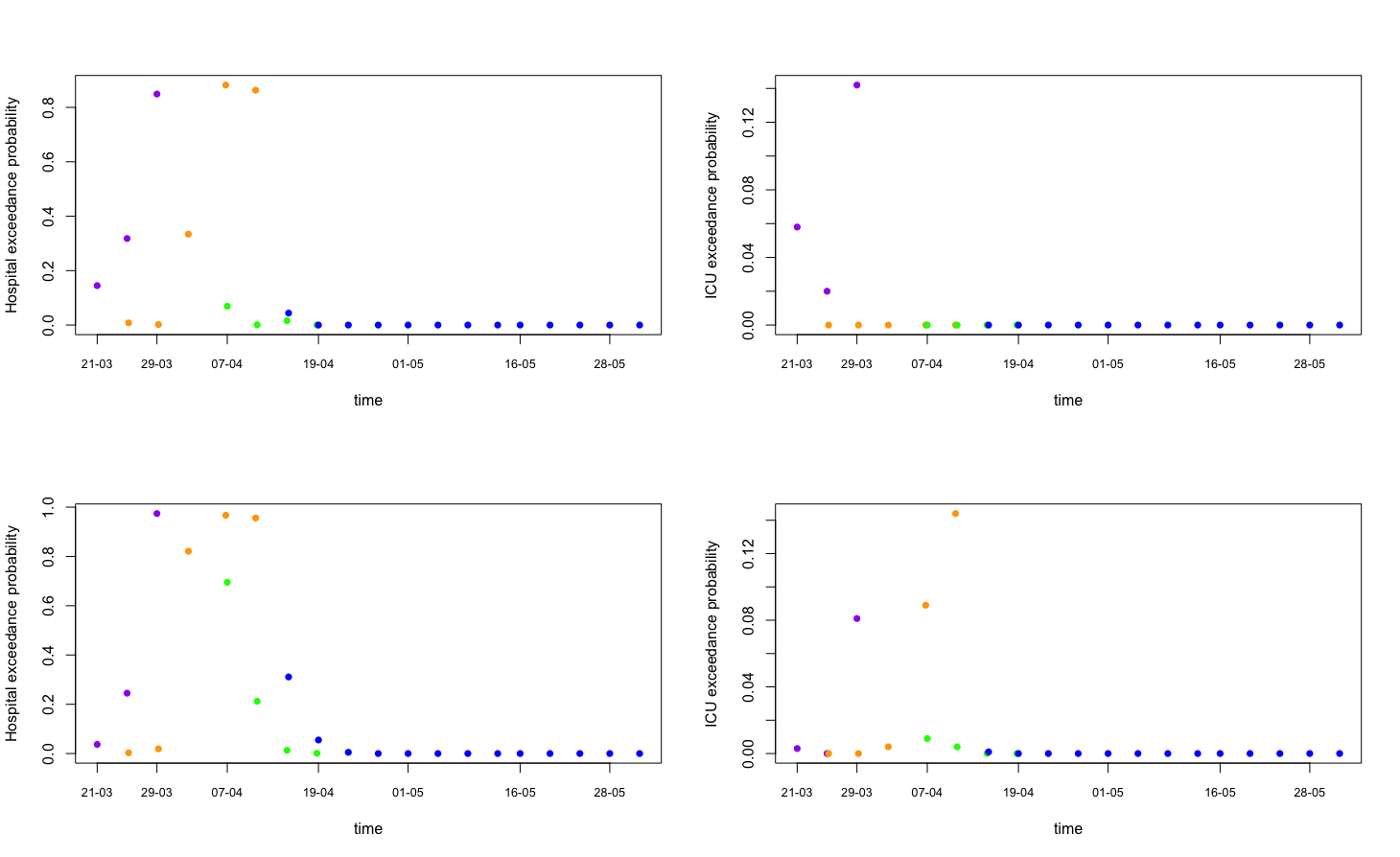

Supplement: Supplementary file 1 [file S0950268821002491sup001.zip › Final Version/figure/Fig7.tiff]

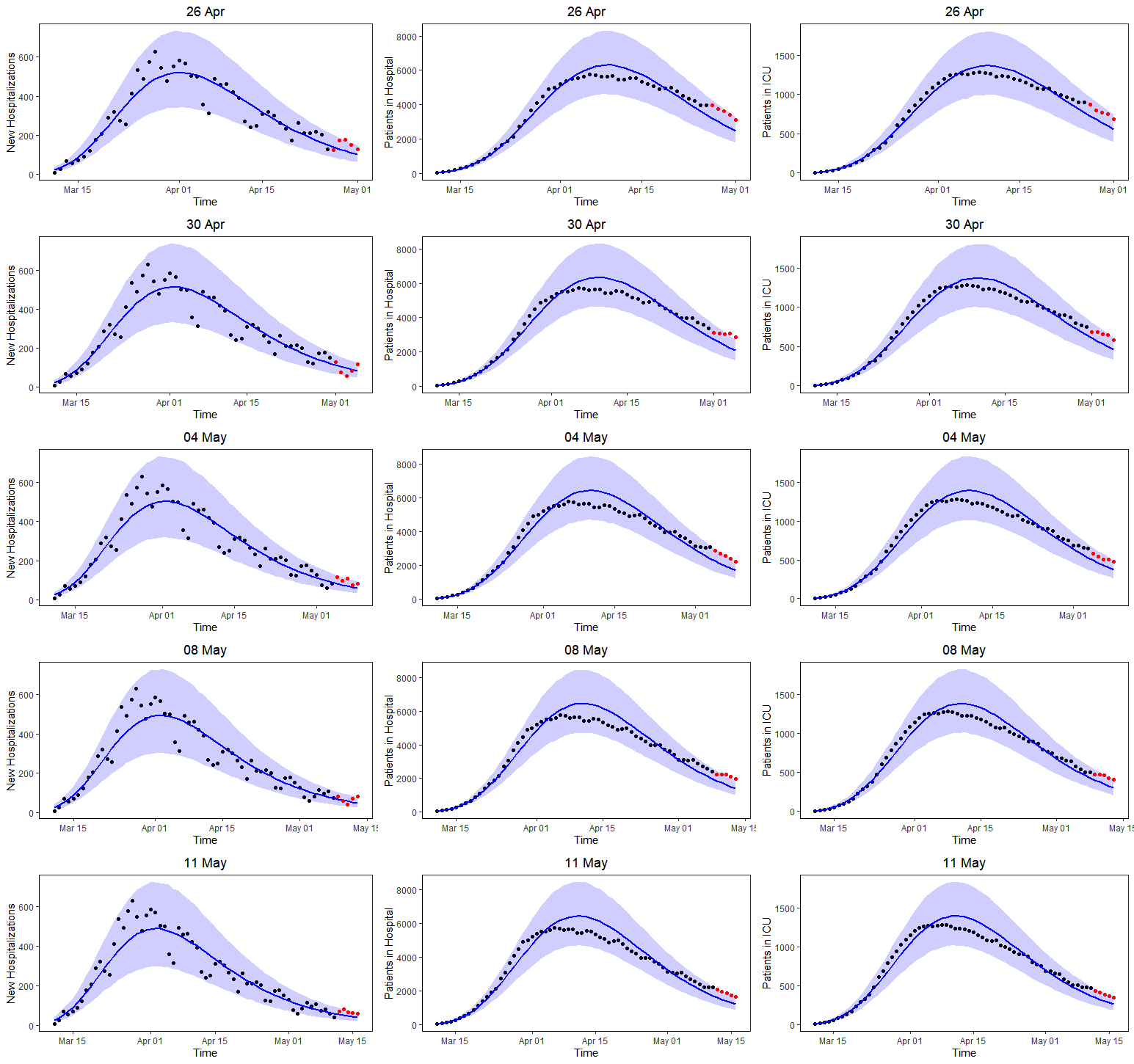

Supplement: Supplementary file 1 [file S0950268821002491sup001.zip › Final Version/figure/appx_Fig3_03.tiff]

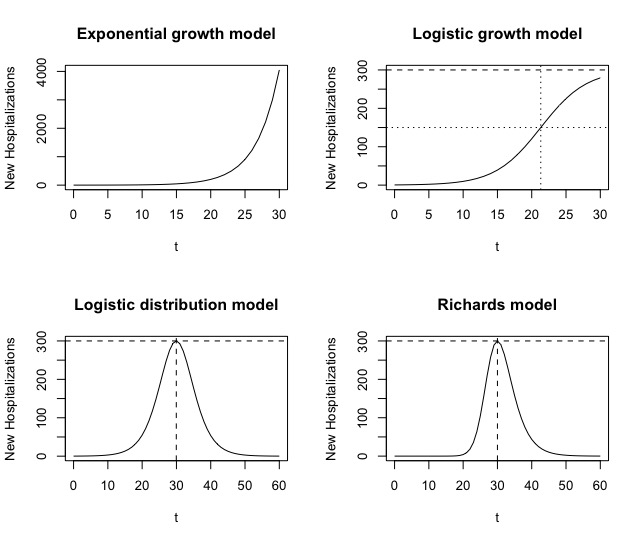

Supplement: Supplementary file 1 [file S0950268821002491sup001.zip › Final Version/figure/growthmodels.tiff]

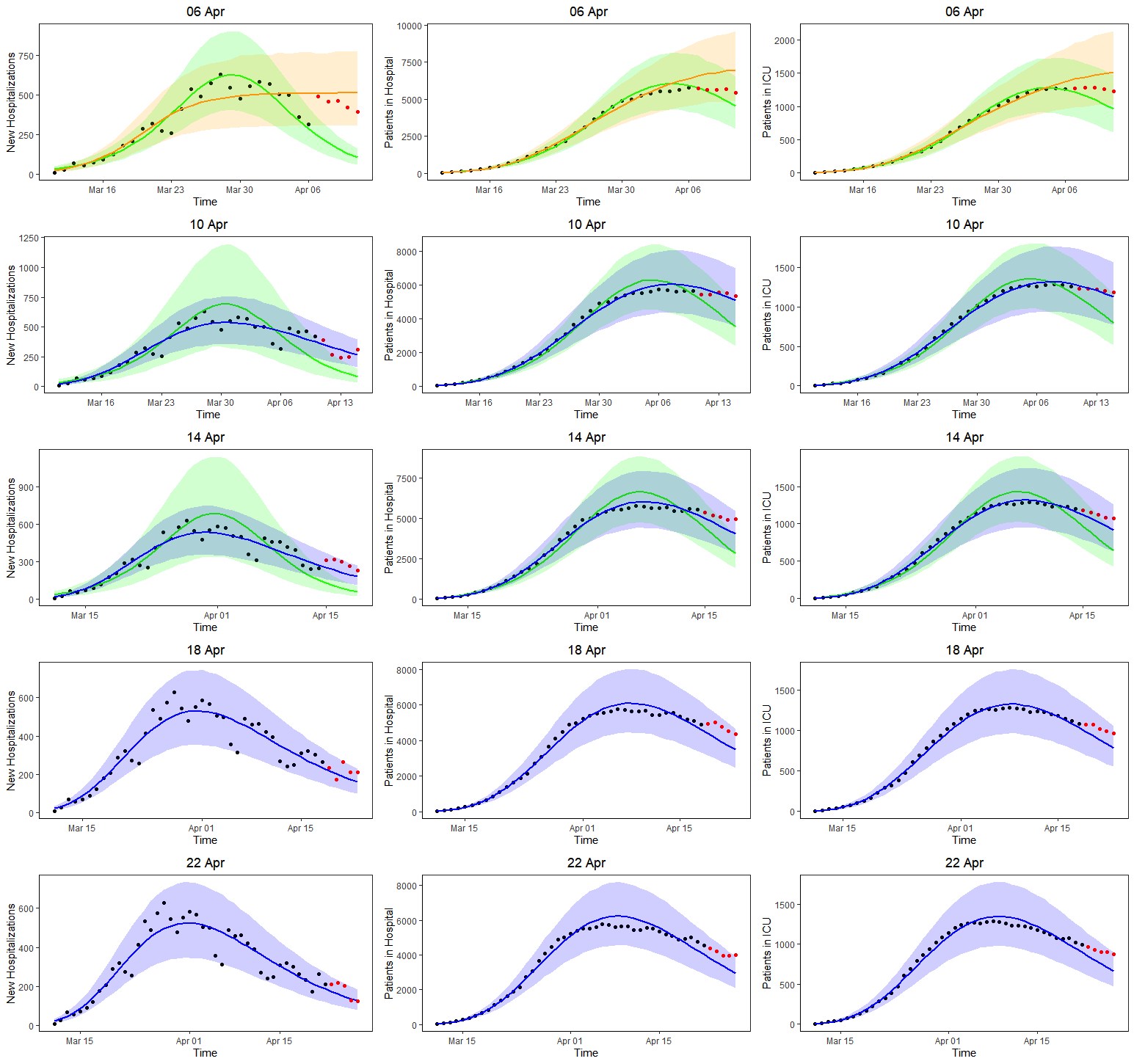

Supplement: Supplementary file 1 [file S0950268821002491sup001.zip › Final Version/figure/appx_Fig3_02.tiff]

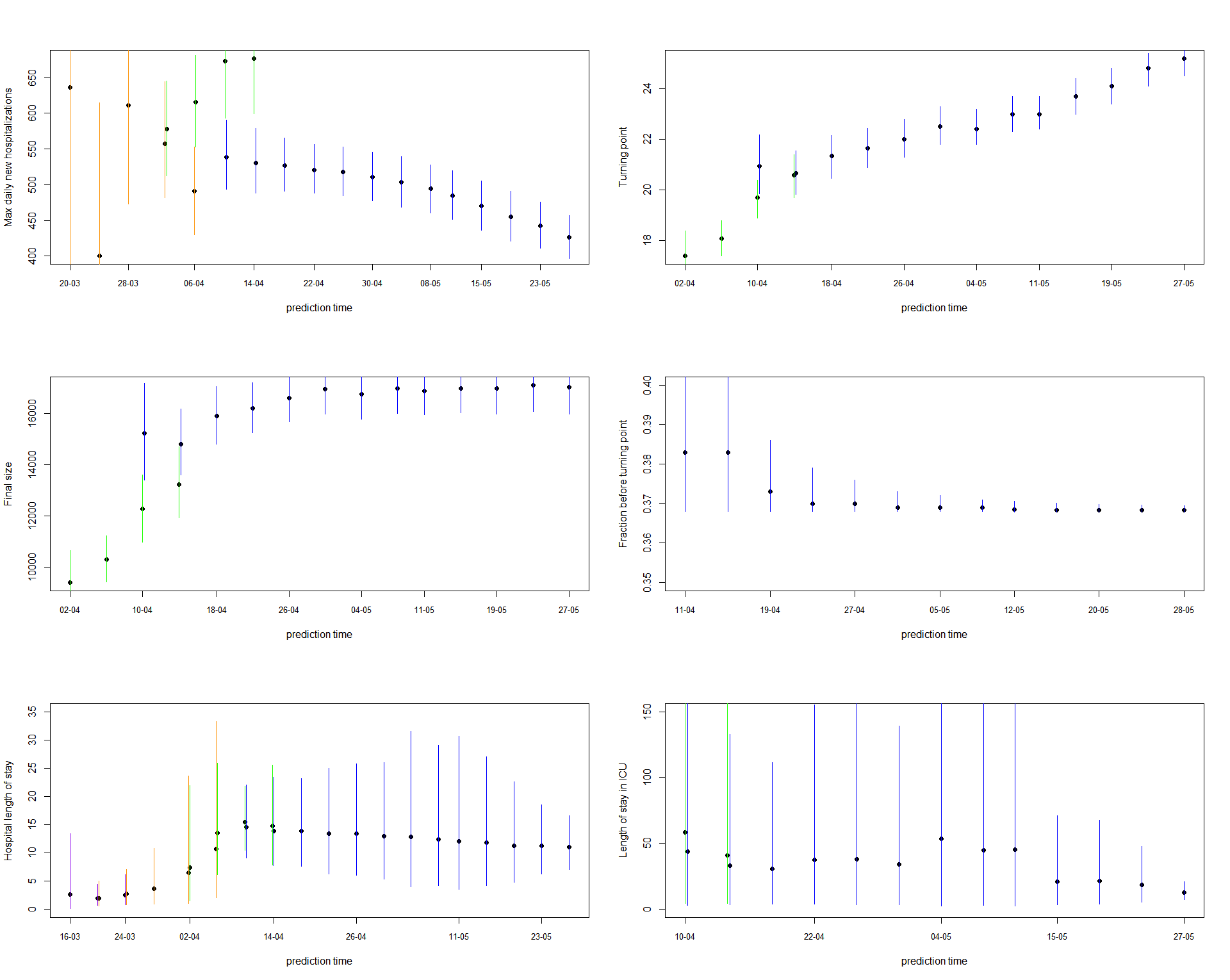

Supplement: Supplementary file 1 [file S0950268821002491sup001.zip › Final Version/figure/Fig6.tiff]

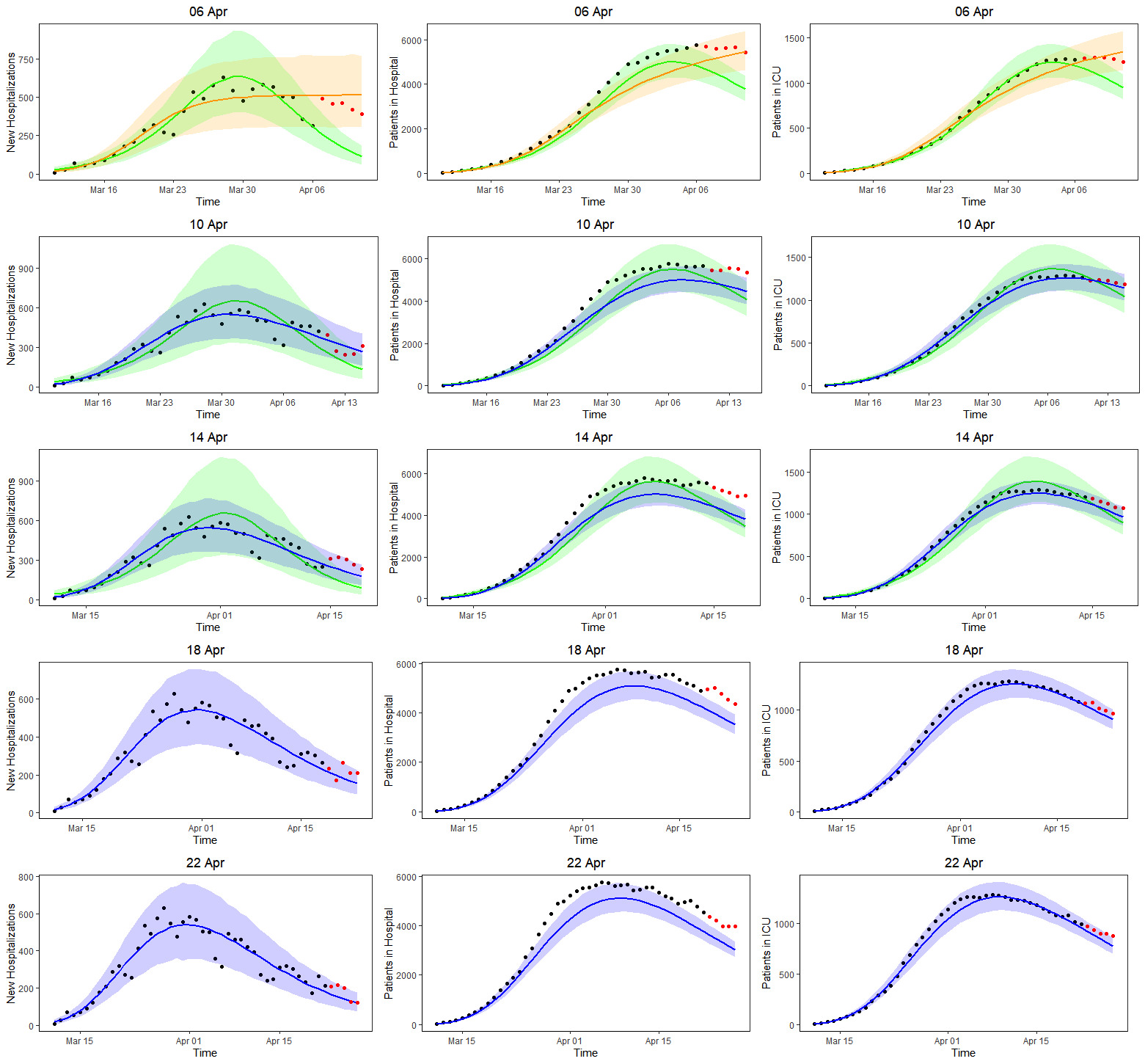

Supplement: Supplementary file 1 [file S0950268821002491sup001.zip › Final Version/figure/appx_Fig1_02.tiff]

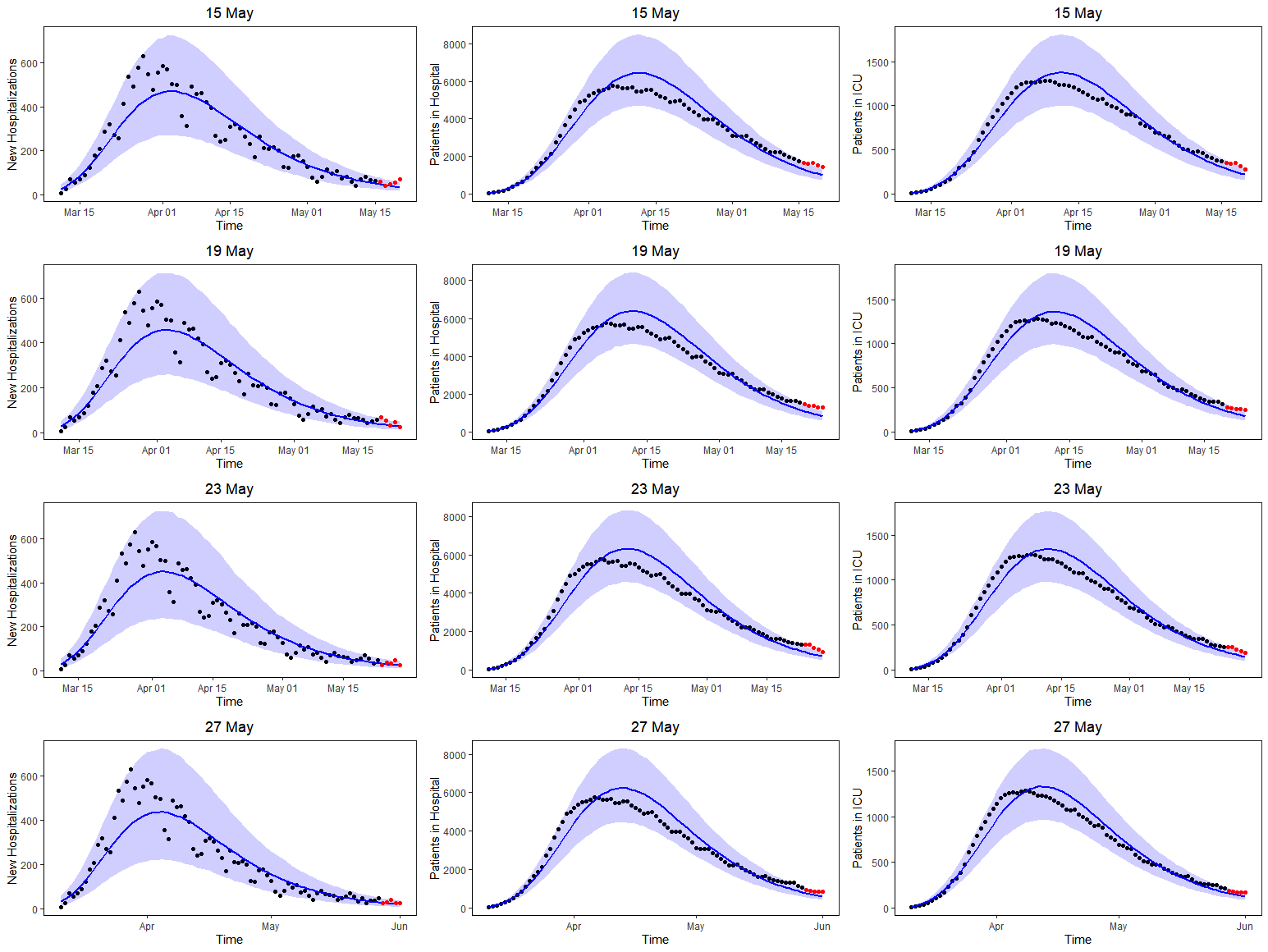

Supplement: Supplementary file 1 [file S0950268821002491sup001.zip › Final Version/figure/appx_Fig3_04.tiff]

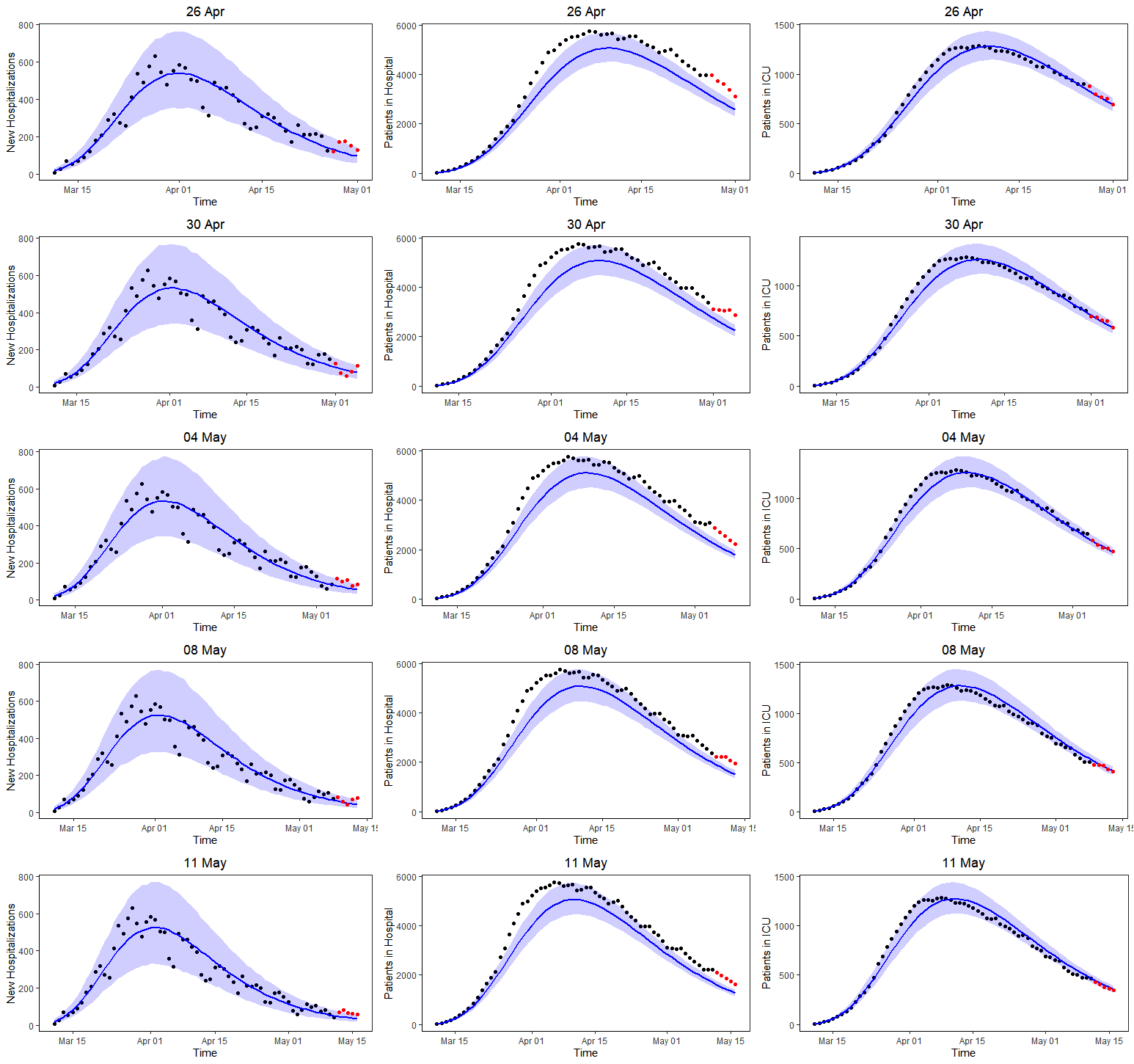

Supplement: Supplementary file 1 [file S0950268821002491sup001.zip › Final Version/figure/appx_Fig1_03.tiff]

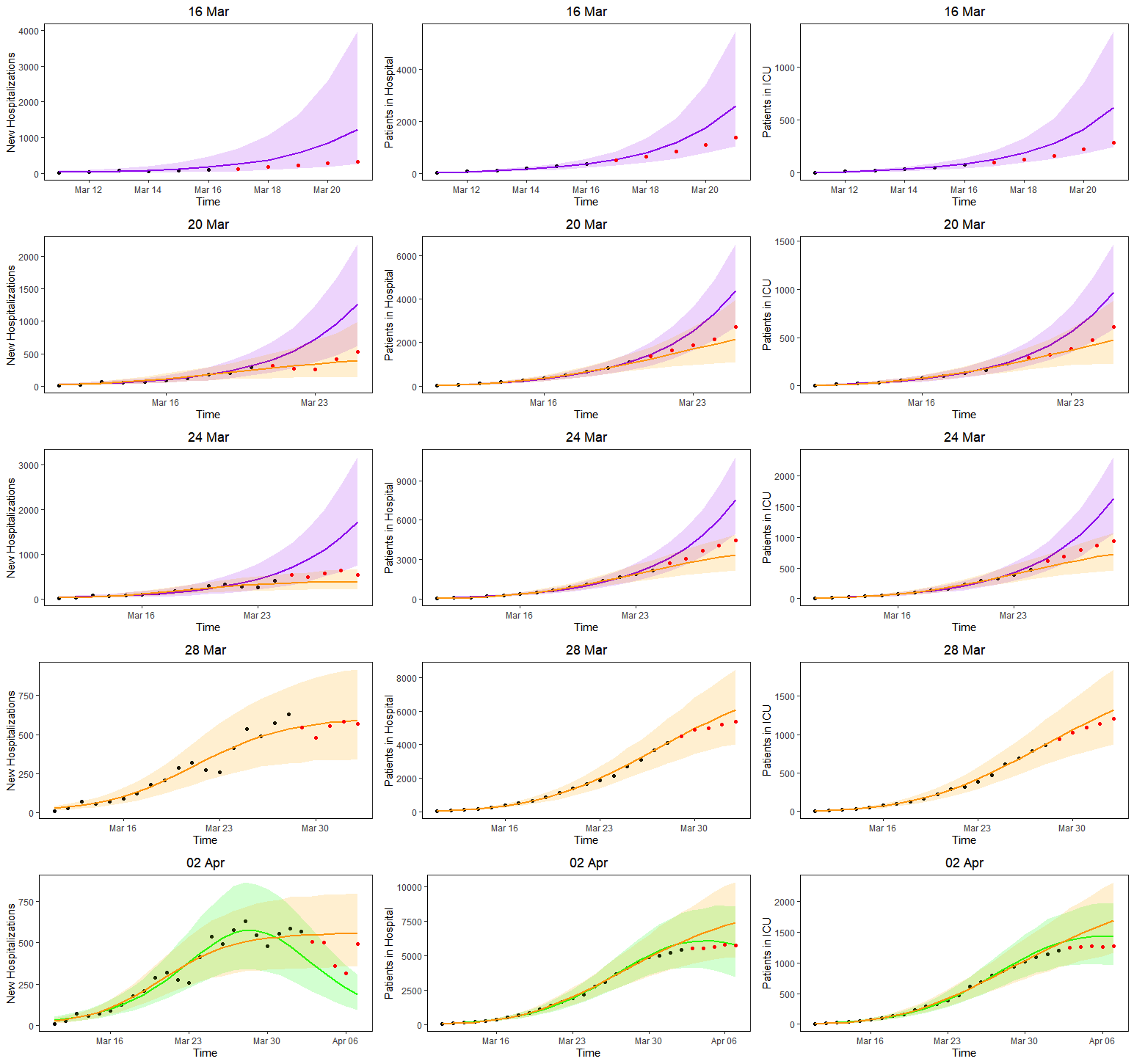

Supplement: Supplementary file 1 [file S0950268821002491sup001.zip › Final Version/figure/Fig5_01.tiff]

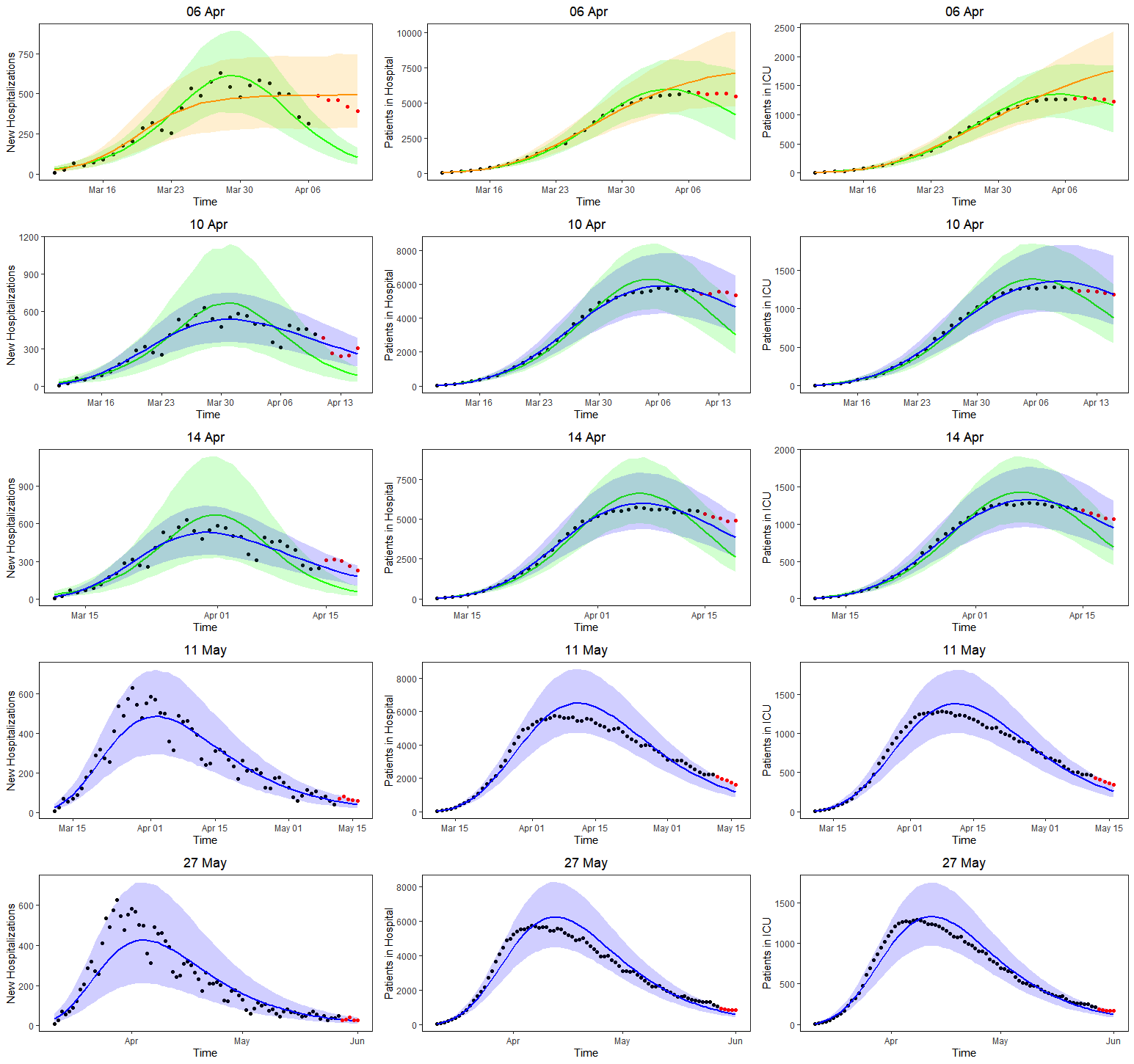

Supplement: Supplementary file 1 [file S0950268821002491sup001.zip › Final Version/figure/Fig5_02.tiff]

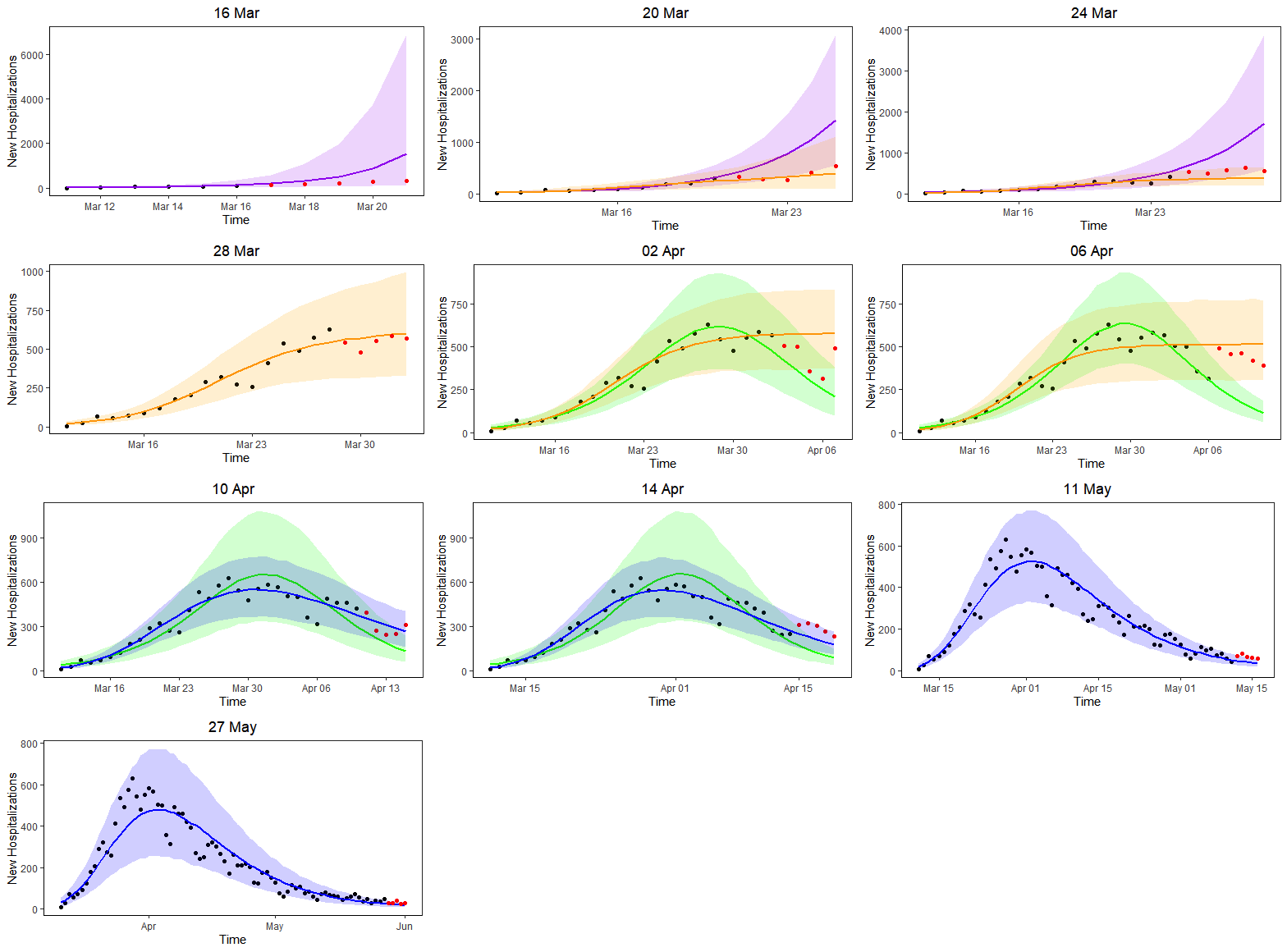

Supplement: Supplementary file 1 [file S0950268821002491sup001.zip › Final Version/figure/Fig3.tiff]

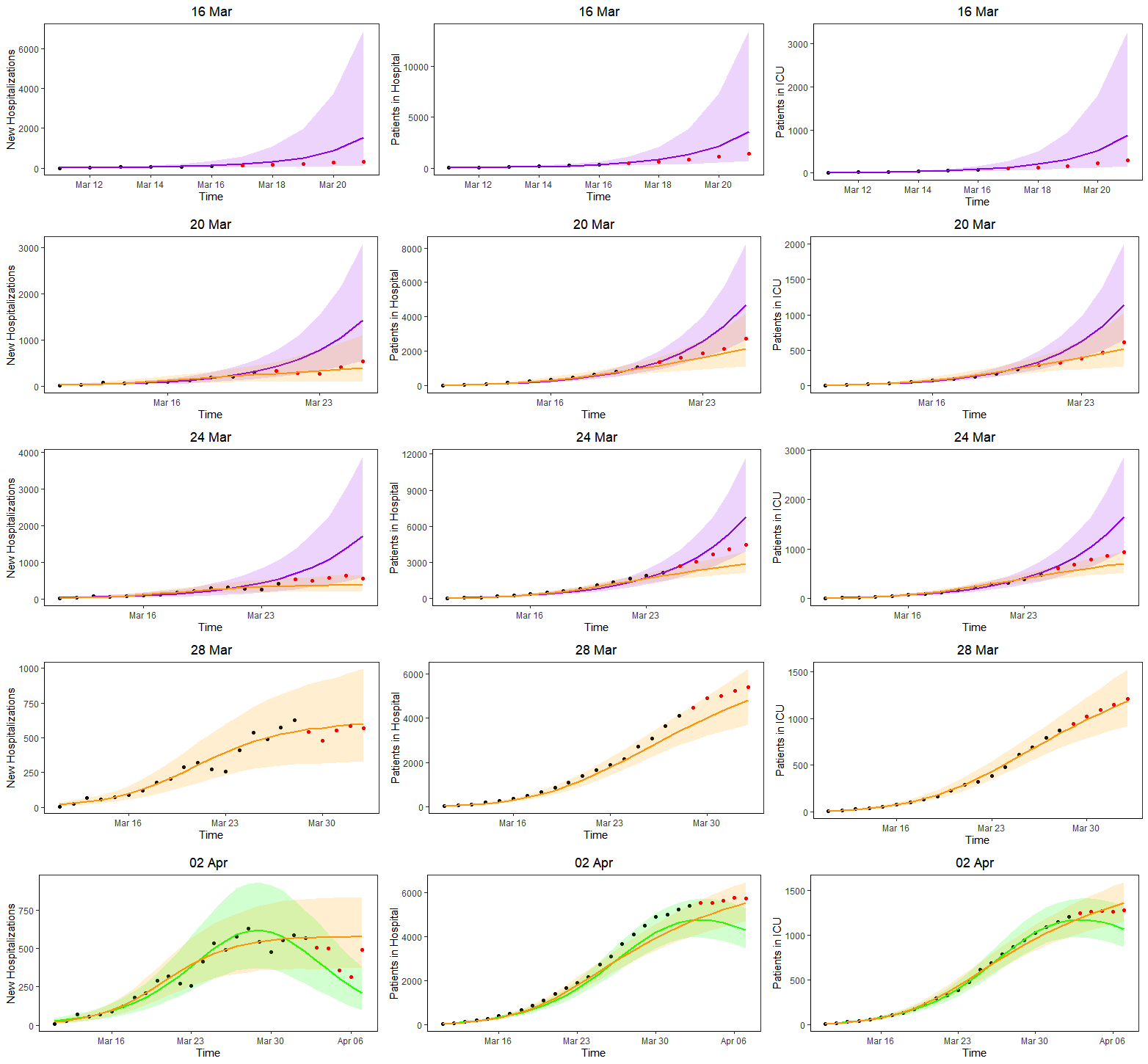

Supplement: Supplementary file 1 [file S0950268821002491sup001.zip › Final Version/figure/appx_Fig1_01.tiff]

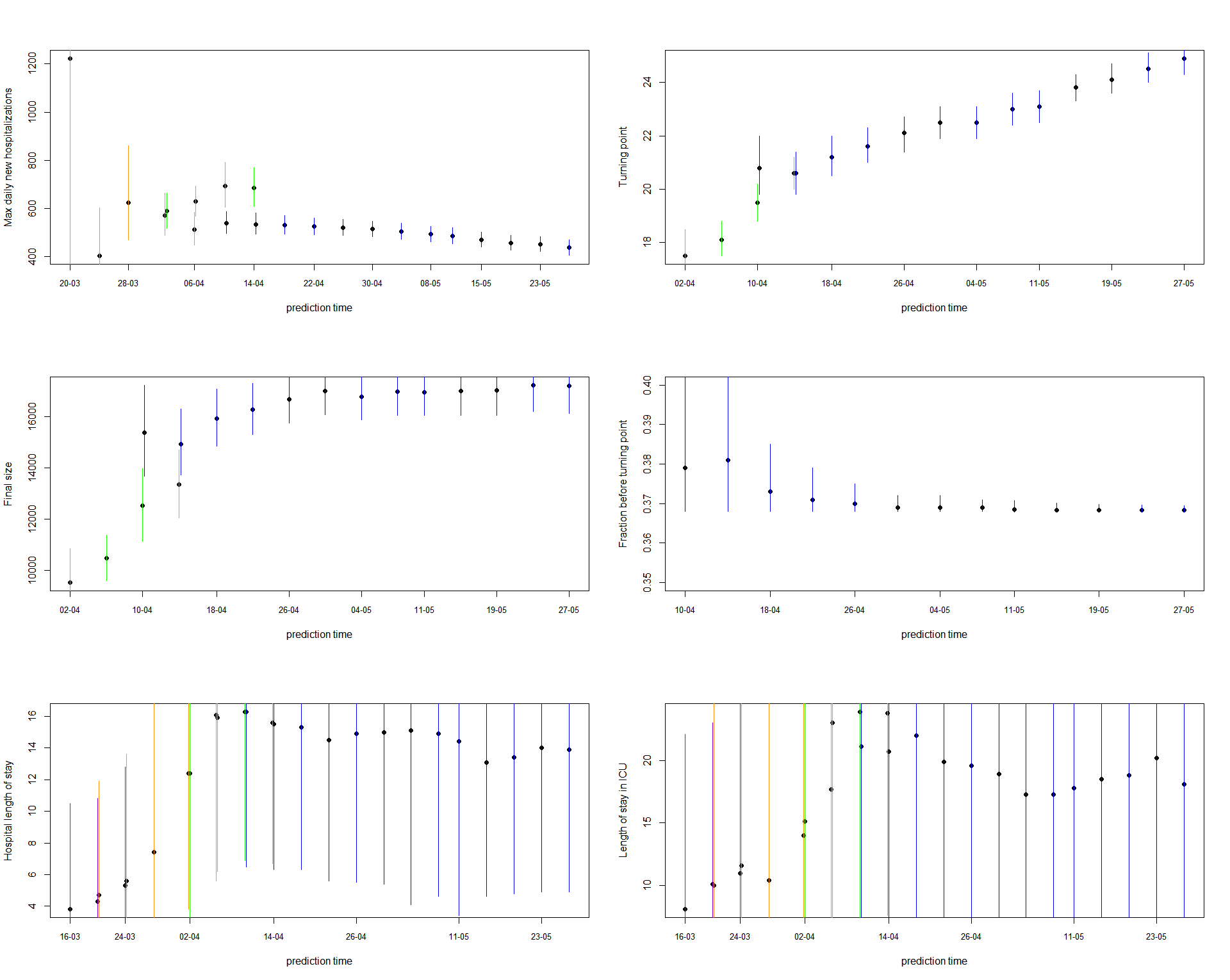

Supplement: Supplementary file 1 [file S0950268821002491sup001.zip › Final Version/figure/appx_Fig4.tiff]

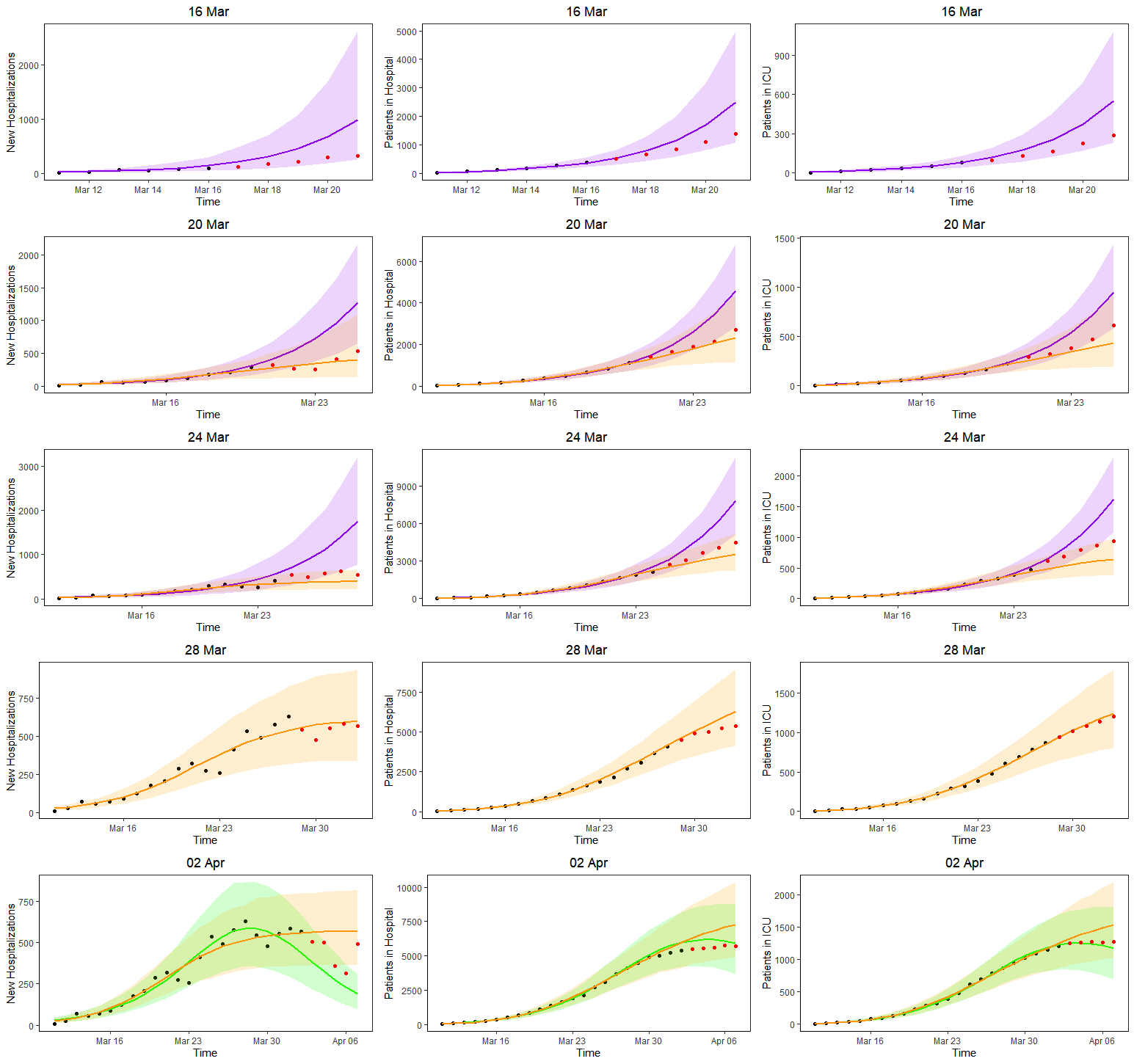

Supplement: Supplementary file 1 [file S0950268821002491sup001.zip › Final Version/figure/appx_Fig3_01.tiff]

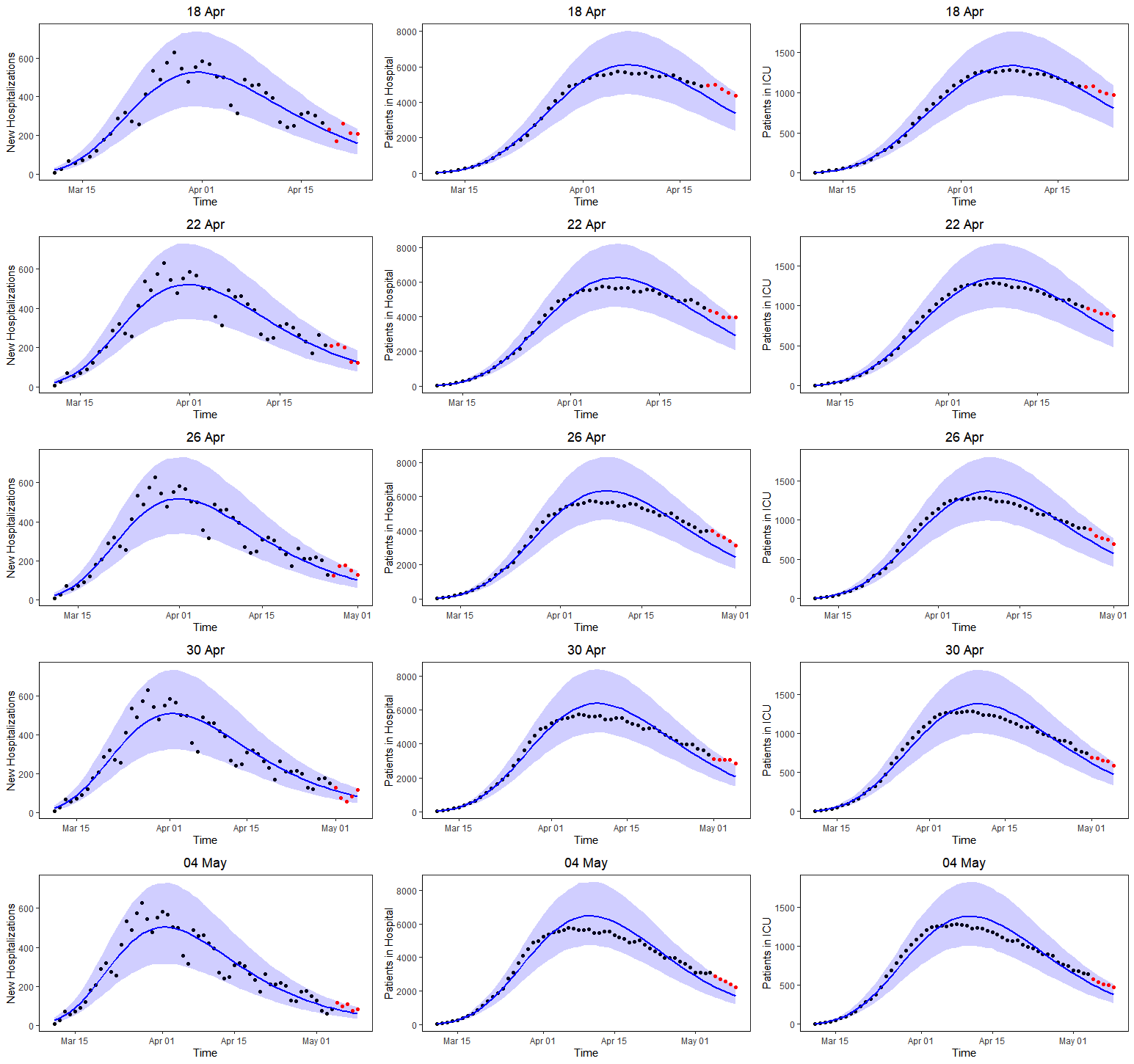

Supplement: Supplementary file 1 [file S0950268821002491sup001.zip › Final Version/figure/appx_Fig2_01.tiff]

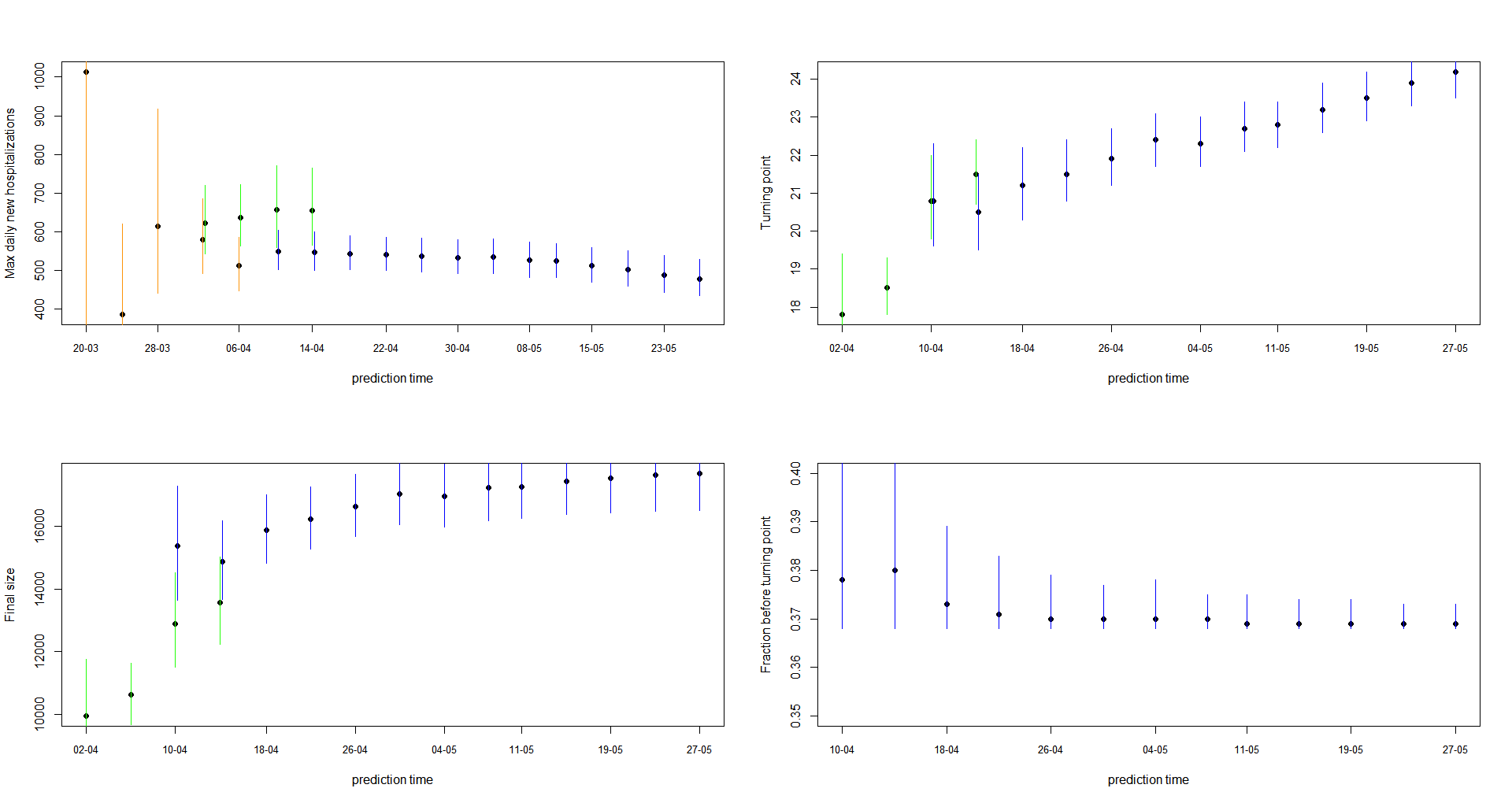

Supplement: Supplementary file 1 [file S0950268821002491sup001.zip › Final Version/figure/Fig4.tiff]

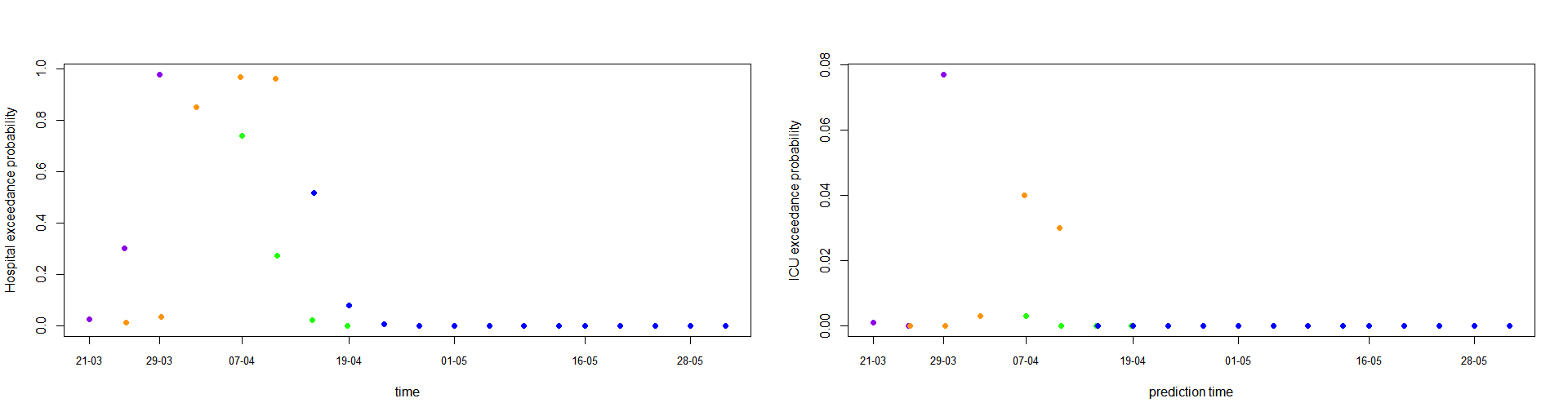

Supplement: Supplementary file 1 [file S0950268821002491sup001.zip › Final Version/figure/appx_Fig5.tiff]

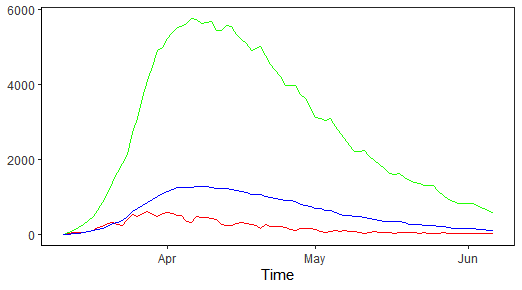

Supplement: Supplementary file 1 [file S0950268821002491sup001.zip › Final Version/figure/Figure_1_case number.tiff]
